# Fluoride Enhances Alcohol Binding Within a Trigonal‐Prismatic Metal‐Organic Capsule

**DOI:** 10.1002/anie.202505137

**Published:** 2025-05-19

**Authors:** Yuchong Yang, Tanya K. Ronson, Dingyu Hou, Kai H. Luo, Jonathan R. Nitschke

**Affiliations:** ^1^ Yusuf Hamied Department of Chemistry University of Cambridge Cambridge CB2 1EW U.K.; ^2^ Department of Mechanical Engineering University College London London WC1E 7JE U.K.; ^3^ Beijing National Laboratory for Molecular Sciences, Institute of Chemistry Chinese Academy of Sciences Beijing 100190 P.R. China

**Keywords:** Endo‐functionalization, Host–guest chemistry, Metal‐organic cages, Post‐assembly modification, Self‐assembly

## Abstract

Herein we utilize the binding of fluoride to boron atoms to functionalize the interior of a boron‐containing trigonal prismatic capsule that incorporates two triangular and three rectangular ligands, enabling the tuning of its guest binding properties. The methyl groups of the triangular ligands guide the rectangular ligands to adopt a “landscape” orientation to avoid steric hindrance. This small structural change gives rise to an enlarged interior cavity volume for guest encapsulation, as compared with a previously‐reported trigonal prismatic capsule, where the same rectangular ligand took a “portrait” orientation with a non‐methylated triangular ligand of similar size. The methylated triangular ligand contains a boron core, which can bind fluoride ions that point inward. These bound fluorides serve as hydrogen bond acceptors, which increases the affinity of the capsule for hydrogen‐bond‐donating alcohols, which are bound in preference to ketones of similar sizes. Moreover, this boron‐containing trigonal prism selectively binds perrhenate over perchlorate, while fluoride binding modulates the cavity charge, leading to perrhenate ejection. These and similar *endo*‐functionalized capsules may thus be of use in the fields of molecular recognition and separation.

## Graphical abstract

Steric hindrance from methyl groups orients rectangular ligands “landscape”‐wise, forming a trigonal prismatic capsule with a large cavity. Endohedral functionalization of this capsule via fluoride binding tunes its guest affinity, enhancing the binding of alcohols over ketones and enabling selective perrhenate encapsulation and release, offering an approach to molecular recognition and separation.

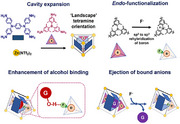

Biomolecular recognition and binding are fundamental processes in natural systems that enable various functions within living systems, including enzyme‐substrate binding, antibody–antigen recognition, and protein–protein interactions.^[^ ^1^, ^2^, ^3^ ^]^ The shape of an enzyme binding pocket and the surrounding functional moieties are critical factors that drive binding.^[^ ^4^, ^5^ ^]^ Synthetic molecular receptors draw inspiration from these specific binding behaviors for applications in catalysis,^[^ ^6^, ^7^ ^]^ molecular capture,^[^ ^8^, ^9^, ^10^ ^]^ and molecular recognition and sensing,^[^ ^11^, ^12^ ^]^ but designing capsules with specific inward‐facing functionality remains a significant challenge.^[^ ^13^ ^]^

The guest‐binding abilities of metal‐organic capsules render them useful for various applications,^[^ ^14^, ^15^, ^16^, ^17^, ^18^, ^19^, ^20^ ^]^ including molecular capture and purification,^[^ ^21^, ^22^, ^23^ ^]^ catalysis,^[^ ^24^, ^25^, ^26^ ^]^ drug delivery,^[^ ^27^, ^28^, ^29^, ^30^ ^]^ and stabilizing reactive species.^[^ ^31^, ^32^, ^33^, ^34^, ^35^ ^]^ The construction of metal‐organic capsules that feature internally‐directed functionalities represents a promising approach for mimicking the high binding selectivity of biomolecular cavities.

Current approaches to endohedrally‐functionalized metal‐organic cages involve the assembly of pre‐functionalized ligands^[^ ^36^, ^37^, ^38^, ^39^, ^40^ ^]^ or the encapsulation of guest molecules containing functional groups within a non‐functionalized cavity.^[^ ^41^, ^42^ ^]^ The synthesis and assembly of these functionally modified ligands and guest molecules can present challenges^[^ ^43^ ^]^ that complicate the preparation of the desired capsules.

Dynamic post‐assembly modification^[^ ^44^, ^45^ ^]^ of metal‐organic capsules has emerged as a promising approach for controlling structural transformations and conformations,^[^ ^34^, ^46^, ^47^, ^48^ ^]^ phase transfer,^[^ ^49^ ^]^ mimicking allosteric regulation,^[^ ^50^, ^51^, ^52^ ^]^ and tuning material properties.^[^ ^53^, ^54^ ^]^ In contrast with irreversible modification, dynamic post‐assembly modification mimics the reversible interactions observed among components within biomolecular systems. Dynamic post‐assembly modification of the interiors of metal‐organic capsules may thus enable the preparation of new adaptable and responsive host–guest systems.

Here we report metal‐organic trigonal prism **1** (Figure 1) that contains rectangular tetratopic **L^A^** ligands derived from subcomponent **A** paneling its three quadrilateral faces, and tritopic **L^C^** ligands, derived from subcomponent **C**, that cap its two triangular faces. This work builds upon a previous report of trigonal prismatic capsules,^[^ ^55^ ^]^ in which the shorter N^…^N distance of rectangular subcomponent **A** matches the N^…^N length of triangular subcomponent **B** (Figure 1, top), forming a cage with a small and narrow cavity. In the present case, the bulk of the six methyl groups of triamine **C** would obstruct this length‐matched arrangement, leading instead to an arrangement where the long side of **A** now matches with the tritopic subcomponent. This arrangement produces a trigonal prismatic capsule in which the **L^A^** ligands adopt a “landscape” orientation within trigonal‐prismatic capsule **1** instead of the previously‐observed “portrait” one,^[^ ^56^ ^]^ leading to a larger internal cavity, which binds a wider range of guests.

**Figure 1:**
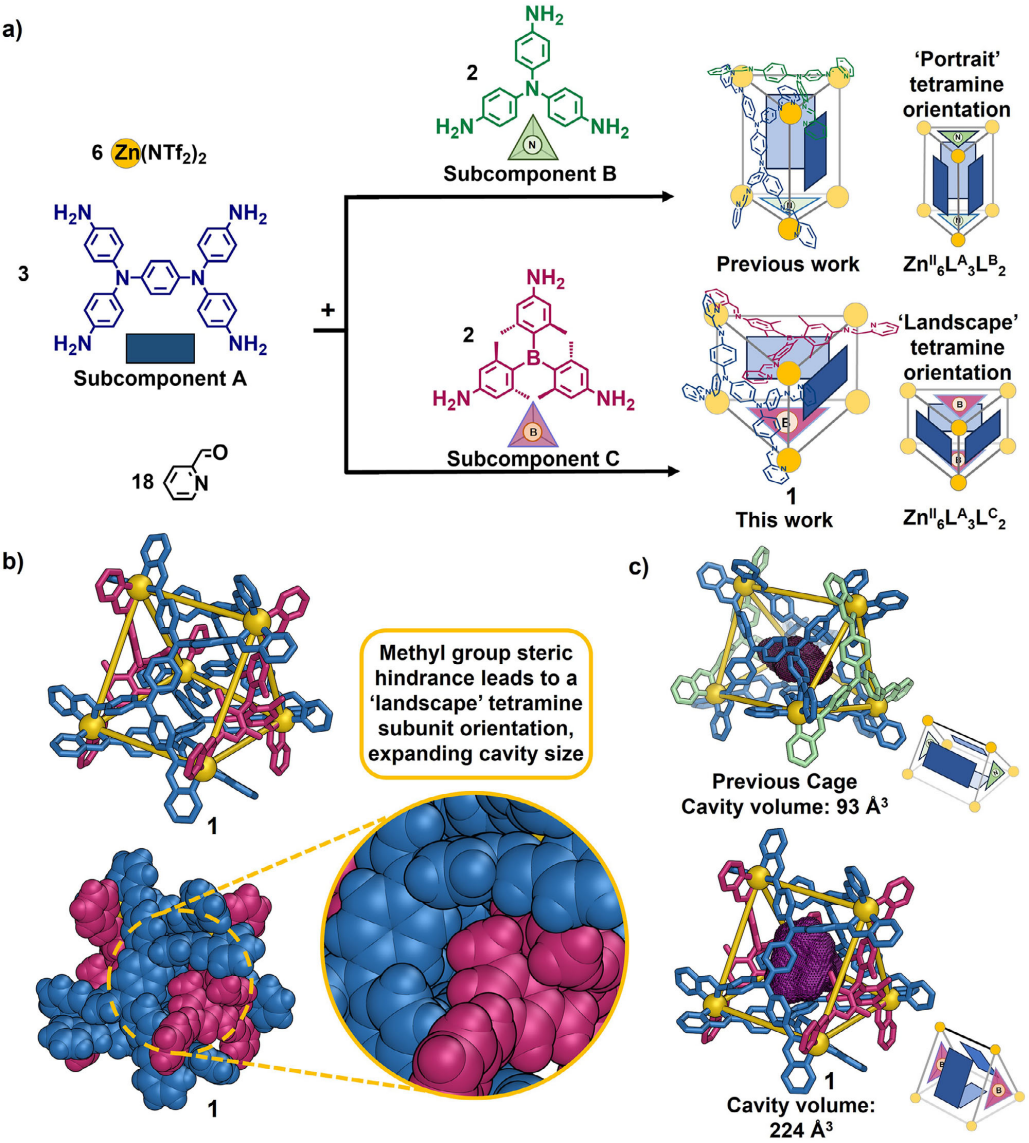
a) Self‐assembly of our previously‐reported Zn^II^_6_ **L^A^**_3_ **L^B^**_2_ trigonal prismatic cage and new Zn^II^_6_ **L^A^**_3_ **L^C^**_2_ trigonal prism **1**; b) X‐ray structure of **1** shown in stick (top, without hydrogen atoms) and space‐filling (bottom) modes (disorder and solvents are omitted for clarity). The yellow circle highlights how the steric bulk of the methyl groups in **L^C^** is accommodated within clefts formed by the longer edge of **L^A^** in a “landscape” orientation; c) comparison of the cavity volumes^[^ ^57^ ^]^ (in purple mesh) of the previously‐reported trigonal prismatic capsule (top) and capsule **1** (bottom).

The dynamic formation of B←F^−^ linkages has been previously observed only on the periphery of metal‐organic capsules.^[^ ^49^, ^50^, ^51^ ^]^ Complementing and building upon work using hydrogen bond acceptors embedded within capsules,^[^ ^58^, ^59^, ^60^ ^]^ here we hypothesized that negatively‐charged fluoride would coordinate endohedrally to the sp^2^‐hybridized boron atoms at the centers of **L^C^** within **1**, promoting the binding of hydrogen bond donors in the cavity, such as alcohols. This hypothesis was validated by the observation that **1**·2F^−^ binds alcohols more strongly than structurally similar ketones. Furthermore, the incorporation of fluoride anions within the cavity also modulated the electrostatic environment, leading to the ejection of negatively‐charged guests. This use of fluoride binding to promote guest ejection may enable new molecular recognition and purification applications.

A previously‐reported Zn^II^_6_ **L^A^**_3_ **L^B^**_2_ trigonal prismatic capsule (Figure 1a)^[^ ^55^ ^]^ incorporates rectangular ligand **L^A^** with its short edge towards size‐matched tritopic ligand **L^B^** in a “portrait” orientation (**L^X^** corresponds to a pyridyl‐imine ligand incorporating subcomponent **X**). Here, subcomponents **A** (3 equiv), **C** (2 equiv) and 2‐formylpyridine (18 equiv) were observed to react with zinc(II) bis‐(trifluoromethanesulfonyl)imide (Zn(NTf_2_)_2_, 6 equiv) in acetonitrile to produce Zn^II^_6_ **L^A^**_3_ **L^C^**_2_ trigonal prismatic capsule **1**, with **L^C^** panels in a “landscape” orientation (Figure 1a).

The formation of **1** was confirmed by 1D and 2D NMR spectroscopy and electrospray ionization mass spectrometry (ESI‐MS), shown in Figures S1–S9. The ^1^H NMR spectrum of **1** displayed 2‐fold desymmetrization of **L^A^**, with maintenance of 3‐fold rotational symmetry of tritopic **L^C^**, consistent with the *D*_3_ point symmetry of a trigonal prism in solution. The ^1^H NMR diffusion‐ordered spectroscopy (DOSY) spectrum of **1** gave a hydrodynamic radius of 16.6 Å (Figure S7).

The solid‐state structure of **1** was determined by single‐crystal X‐ray diffraction at the Diamond Light Source synchrotron^[^ ^61^ ^]^ (Figure 1b). All boron centers within **1** adopt a planar sp^2^ configuration. The Zn^II^ stereocenters within each cage all share the same Δ or Λ stereochemistry. Zn^II…^Zn^II^ distances on the edges of the triangular faces formed by **L^C^** ligands were 12.7–13.1 Å, longer than the Zn^II…^Zn^II^ distances separated by **L^A^** ligands (11.7–12.2 Å).

In contrast to the previously‐reported trigonal prismatic capsule Zn^II^_6_ **L^A^**_3_ **L^B^**_2_ containing “portrait” **L^A^** ligands,^[^ ^55^ ^]^ Zn^II^_6_ **L^A^**_3_ **L^C^**_2_ capsule **1** surrounds a larger internal cavity as a consequence of the “landscape” configurations adopted by its **L^A^** ligands. This ligand reconfiguration led to an increase in calculated cavity volume^[^ ^57^ ^]^ from 93 Å^3^, in the case of the previous cage,^[^ ^55^ ^]^ to 224 Å^3^ for **1**. The six methyl groups within tritopic **L^C^** fit well within clefts formed at the longer side of “landscape” **L^A^** within **1** (Figure 1b), whereas steric clash would be incurred if **L^A^** were to adopt a “portrait” orientation. We infer that this steric hindrance overrides the better length match between the shorter side of **L^A^** and **L^C^**.

This larger internal cavity facilitated the encapsulation of a range of guest molecules within **1**. These include alcohols and ketones such as 2‐butanol (**G1**), 2‐butanone (**G2**), 3‐pentanol (**G3**), 3‐pentanone (**G4**), cyclopentanol (**G5**), cyclopentanone (**G6**), cyclohexanol (**G7**), and cyclohexanone (**G8**). All encapsulated molecules were observed to undergo slow exchange with free ones on the ^1^H NMR timescale, as shown in Figures S29, S32, S35, S38, S41, S44, S47, and S50. The formation of eight distinct host–guest complexes, **G1**⊂**1**–**G8**⊂**1**, was confirmed by ESI‐MS, which indicated 1:1 host–guest binding stoichiometry (Figures S29–S52).

The binding of F^−^ to **1** was then investigated by NMR titration (Figures 2a, S10, and S11). Following the addition of tetrabutylammonium fluoride (TBAF) to **1** in CD_3_CN, the color of the solution changed from dark to light red, to first yield intermediate **1**·F^−^, and then **1**·2F^−^. A new ^1^H NMR spectrum emerged, showing only one set of ligand signals after 2.5 equiv TBAF had been added, indicating that the *D*_3_‐symmetric structure of **1** is maintained in **1**·2F^−^. A broad signal appeared at −171 ppm in the ^19^F NMR spectrum of **1**·2F^−^ (Figure S11), corresponding to boron‐bound F^−^. Two distinct ^1^H NMR signals were assigned to the inward‐ and outward‐pointing methyl groups on **L^C^** adjacent to boron, as confirmed by the ^1^H‐^1^H NOESY spectrum (Figures S13 and S14).

**Figure 2:**
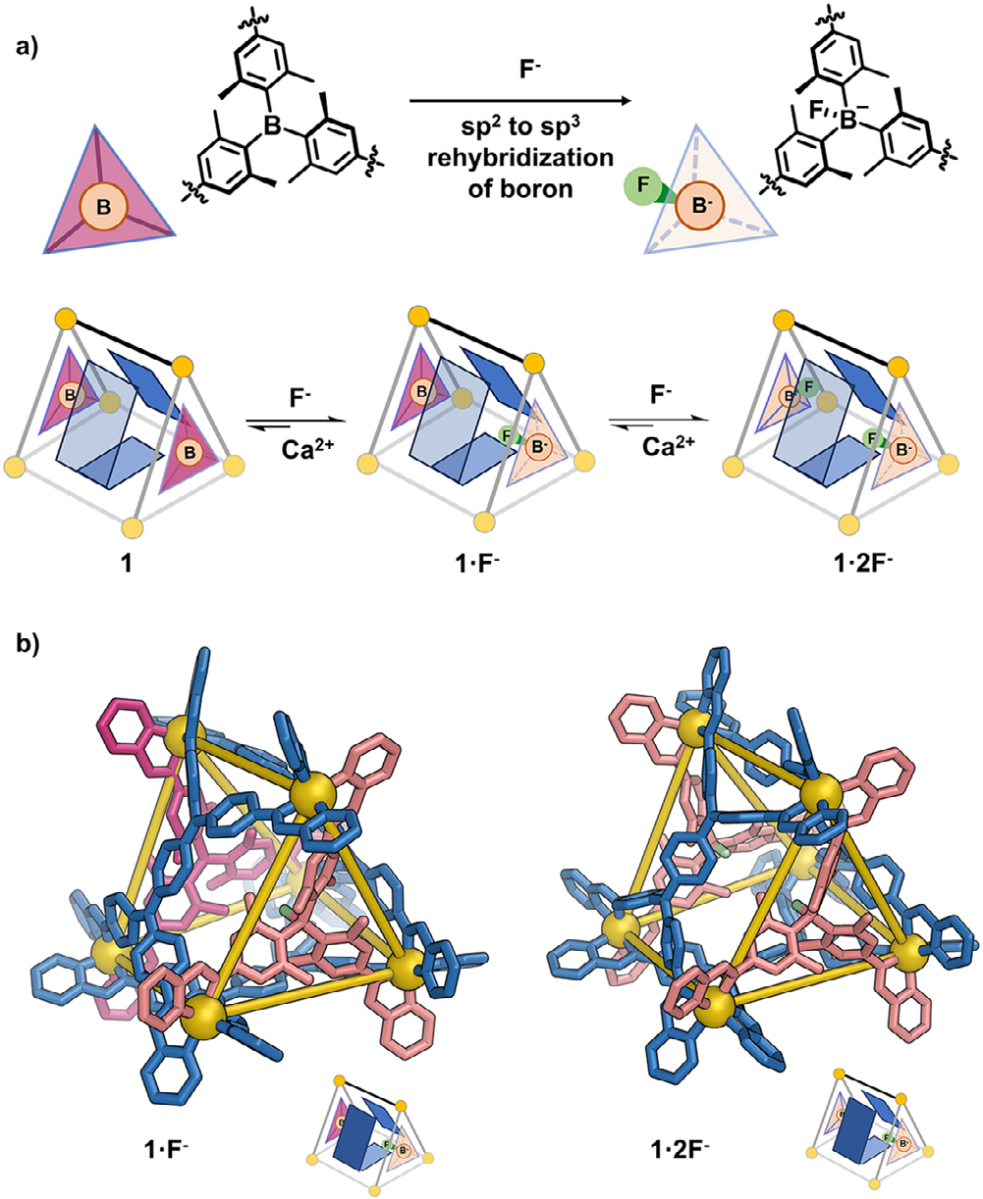
a) Illustration of fluoride binding to **1** and subsequent F^−^ removal upon treatment with Ca^2+^; b) DFT^[^ ^62^ ^]^‐minimized structures of **1**·F^−^ (left) and **1**·2F^−^ (right).

One‐dimensional ^1^H‐^19^F HOESY (heteronuclear overhauser effect spectroscopy) further clarified the relative positions of the boron‐bound fluorides and methyl groups (Figure S15). A correlation was only observed between the inward‐pointing methyl group resonance and the fluoride signal, demonstrating that the fluoride ions are positioned inside the cage.

DFT calculations^[^ ^62^ ^]^ were employed to model putative structures of **1**·2F^−^ with internally and externally bound F^−^. The structure with internal fluoride was favored energetically by 45 kJ mol^−1^, as shown in Figure S77. The heteroleptic ligand arrangement of the trigonal prismatic capsule spatially separates the methyl‐substituted boron‐based moieties, thereby reducing steric hindrance and favoring internal F^−^ binding. The optimized structure of **1**·2F^−^ is shown in Figure 2b. The internal cavity volume of **1**·2F^−^ was estimated^[^ ^57^ ^]^ to be 196 Å^3^ (Figure S78), slightly smaller than that of the parent capsule **1**.

The reversibility of B←F^−^ bonding within **1**·2F^−^ was demonstrated by its reversion to **1** upon Ca(NTf_2_)_2_ addition. The observation that 100 equiv of Ca(NTf_2_)_2_ was required to effect the removal of F^−^ from **1**·2F^−^ implies strong binding of F^−^ to boron, as confirmed by this process yielding a mixture of **1** and **1**·F^−^. This process was confirmed by ^1^H NMR spectroscopy, as shown in Figure S26. Owing to the incomplete nature of the transformation, a systematic evaluation of the reversibility of guest ejection was not undertaken.

The binding affinities of guests **G1**−**G8** to both **1** and **1**·2F^−^ were systematically investigated. Binding constants for the complexes **G1**⊂**1**−**G8**⊂**1** and **G1**⊂**1**·2F^−^−**G8**⊂**1**·2F^−^ were determined through NMR titration.^[^ ^63^ ^]^ The results are presented in Table 1 and Figures S29–S68. Some selectivity for binding alcohols over structurally similar ketones was observed for **1**, with selectivity values (selectivity = binding constant of alcohol/binding constant of ketone) ranging from 2.5 to 6.4.

**Table 1:** Binding constants^a)^ and corresponding selectivity values.

Binding constants (/M^−1^) of G1–G8 Top: for 1 *Bottom: for 1·2F* ^−^				
Alcohol	Binding constant	Ketone	Binding constant	Selectivity
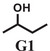	**32.1 ± 6.1**	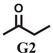	**5.15 ± 1.3**	**6.23**
	** *254 ± 56* **		** *<1* **	** *>254* **
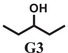	**26.1 ± 8.1**	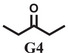	**4.05 ± 1.1**	**6.44**
	** *210 ± 35* **		** *<1* **	** *>210* **
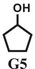	**37.8 ± 7.4**	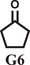	**10.1 ± 0.4**	**3.74**
	** *286 ± 48* **		** *52.5 ± 8.9* **	** *5.45* **
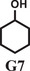	**26.9 ± 4.8**	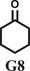	**10.6 ± 1.5**	**2.54**
	** *79.4 ± 8.8* **		** *19.5 ± 4.9* **	** *4.07* **

^a)^ For **G1–G8** to **1** (red) and **1**·2F^−^ (italic and blue).

Since the fluorides within **1**·2F^−^ might act as hydrogen bond acceptors, we hypothesized that the fluoride‐bound cage might display greater discrimination in binding alcohols over ketones. A 1:1 host–guest binding ratio was again observed for **1**·2F^−^ as with **1**, as indicated by ESI‐MS. As hypothesized, the presence of fluoride improved the selectivity for alcohols over ketones. As shown in Table 1, **1**·2F^−^ bound the alcohols **G1** and **G3** exclusively while showing no interactions with ketones **G2** and **G4**, as confirmed by ^1^H NMR and ESI‐MS (Figures S55–S56 and S59–S60). Similarly, the selectivity of binding for **1**·2F^−^ vs. **1** between alcohols and ketones was improved for **G5**/**G6** and **G7**/**G8**.

To gain deeper insight into the mechanism of enhanced alcohol binding, we performed DFT optimization^[^ ^62^ ^]^ of the structure of model complex **G1**⊂**1**·2F^−^. The F^−…^O distance in this model was 2.8 Å (Figure S80), within the typical hydrogen bonding distance range (2.5–3.2 Å).^[^ ^64^ ^]^ We infer that F^−…^H─O hydrogen bonding may play a key role in enhancing the binding affinity of alcohols within **1**·2F^−^, in contrast with ketones, which are poor hydrogen bond donors. Furthermore, the partial negative charge on the ketone carbonyl oxygen may repel F^−^, disfavoring the binding of **G2** and **G4** within **1**·2F^−^. These two effects may thus serve to drive selectivity of alcohol binding over ketones within **1**·2F^−^. Fluoride‐binding cages such as **1** may thus enable new solutions for the challenging industrial separation^[^ ^65^, ^66^, ^67^ ^]^ of structurally similar alcohols and ketones.

We further investigated the binding of diverse prospective guests within **1** and **1**·2F^−^, including the anions hexafluoroantimonate (**G9**), perrhenate (**G10**) and perchlorate (**G10**), perfluorinated hexafluorobenzene (**G12)** and octafluorocyclopentene (**G13**), and cationic tetramethylammonium (**G14**). Host **1** bound anions **G9** and **G10** (Figure 3a), with binding constants of (7.05 ± 0.72) × 10^3^ M^−1^ and (1.31 ± 0.14) × 10^4^, respectively (Figures S69 and S70). Competitive guest binding was investigated between **G9** and **G10** within **1**, as shown in Figure S76. Despite the physicochemical similarities between perrhenate (**G10**) and perchlorate (**G11**), which present challenges for their separation, **1** was observed to bind **G10** but not **G11** significantly. This observation suggests that the cavity of **1** distinguishes subtle differences in size and charge distribution between **G10** and **G11**.

**Figure 3:**
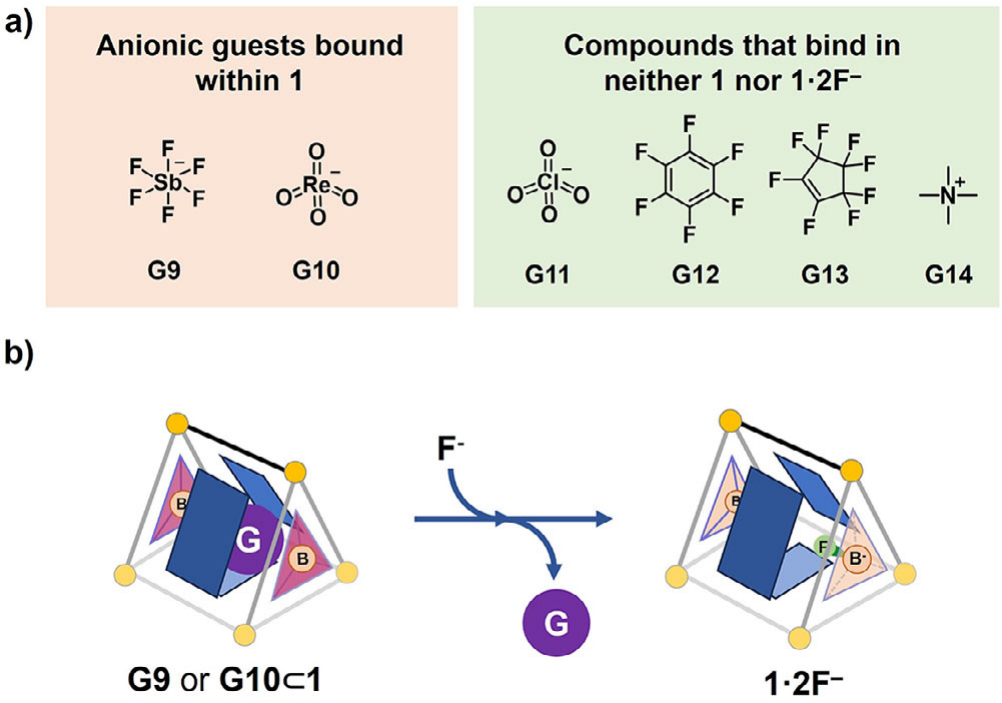
a) Prospective guests were tested for binding within **1** and **1**·2F^−^. **G9** and **G10** were observed to bind within **1**, but not **1**·2F^−^, and **G11–G14** bound within neither cage. b) **G9** and **G10** were released from **1** following the formation of **1**·2F^−^.

Although the framework of **1**·2F^−^ retains an overall positive charge, the incorporation of fluoride anions within the cavity modulates the local electrostatic environment, rendering it less suitable for negatively charged species. Consequently, anionic guests **G9** and **G10** were ejected from **1** following the formation of **1**·2F^−^, as shown in Figures 3b, S74, and S75. This process may hold potential applications in nuclear waste extraction, as **G10** is physicochemically very similar to ^99^TcO_4_^−^,^[^ ^68^ ^]^ a constituent of nuclear waste. Such findings underscore the importance of guest binding selectivity and controlled release^[^ ^69^, ^70^, ^71^, ^72^, ^73^, ^74^ ^]^ in the design of functional metal‐organic cages.

The steric hindrance of the methyl groups within subcomponent **A** thus favored the “landscape” orientation of **L^A^** within **1**, expanding its cavity volume. The ability of the central boron within **L^C^** to bind fluoride altered the ability of the cage to discriminate between alcohols and ketones, as well as governing the selective binding and controlled release of ReO_4_^−^ in ways that may enable the design of systems that use cages to effect chemical purification. Future work will explore the preparation of larger polyhedral cages that incorporate boron centers, to explore the use of these cages in selectively binding biomolecules with surface hydroxyl groups, among other prospective targets. Conditions will also be explored that lead to more cleanly reversible fluoride ligation to boron, so as to be able to gear fluoride and alcohol binding together in systems capable of alcohol uptake in one place, then release in purified and concentrated form elsewhere, potentially undergoing phase transfer^[^ ^75^ ^]^ along the way.

## Conflict of Interests

The authors declare no conflict of interest.

## Supporting information



Supporting Information

## Data Availability

The data that support the findings of this study are available in the supplementary material of this article.
