# Supplementary material for: Fluoride Enhances Alcohol Binding Within a Trigonal‐Prismatic Metal‐Organic Capsule
Source: Angew Chem Int Ed Engl. 2025 May 19;64(29):e202505137. doi: 10.1002/anie.202505137 (PMC12258696; doi:10.1002/anie.202505137)
Supplement: Supplementary file 1 — Supporting Information [file ANIE-64-e202505137-s001.pdf]

Supporting Information for

Fluoride Enhances Alcohol Binding within a Trigonal-Prismatic Metal-Organic Capsule

Yuchong Yang,[a] Tanya K. Ronson,[a] Dingyu Hou,[b,c] Kai H. Luo,[b] Jonathan R. Nitschke*[a]

[a] Yusuf Hamied Department of Chemistry, University of Cambridge, Cambridge CB2 1EW, United Kingdom.

[b] Department of Mechanical Engineering, University College London, London, WC1E 7JE, United Kingdom.

[c] Beijing National Laboratory for Molecular Sciences, Institute of Chemistry, Chinese Academy of Sciences, Beijing 100190, PRC.

* To whom correspondence should be addressed: jrn34@cam.ac.uk

1. General Information 2

2. Construction and characterization of coordination capsules 3

2.1 Construction of capsule 1 3

2.2 Construction of 1 • 2F⁻ and intermediate 1 • F⁻ 9

3. Host-guest studies 22

3.1 General procedures 22

3.2 Host-guest interactions of 1 with different guest molecules (G1-G8) 24

3.3 Host-guest interactions of 1 • 2F⁻ with different guest molecules (G1-G8) 48

3.4 Host-guest interactions of 1 and 1 • 2F⁻ with different guest molecules (G9-G14) 64

4 X-ray crystallography 69

5 DFT calculations 71

5.1 Calculated energies of 1 with outward and inward fluoride addition 71

5.2 Cavity volume calculation 72

5.3 DFT-minimized host-guest structure of G1⊂1 • 2F⁻ 73

5.4 Cartesian coordinates of 1 • 2F⁻, G1⊂1 • 2F⁻ and 1 • F⁻ 73

6 References 99

1. General Information

Unless otherwise specified, all starting materials were purchased from commercial sources and used as supplied. Subcomponent **A** and Subcomponent **B** are commercially available. Subcomponent **C** was prepared following literature procedures.[1] All experiments were carried out under an inert atmosphere of purified nitrogen or using standard Schlenk techniques. Reactions were stirred using Teflon-coated magnetic stir bars. Elevated temperatures were maintained using Thermostat-controlled silicone oil baths. Organic solutions were concentrated using a rotary evaporator with a diaphragm vacuum pump. Analytical TLC was performed on Merck silica gel 60 F_{254} plates. The TLC plates were visualized by either ultraviolet light. Purification of products was accomplished by flash column chromatography on silica gel 60 (Merck, particle diameter 40-63 μm). Unless otherwise specified, all reagents were purchased from commercial sources and used as received. A microwave reactor from Discover SP-D 80-CEM Corporation was used for the stereochemical induction experiments. Centrifugation of samples was carried out using a Grant-Bio LMC-3000 low speed benchtop centrifuge.

Nuclear Magnetic Resonance (NMR)

NMR experiments were measured on Bruker AVANCE III and NEO (400 and 500 MHz) spectrometers. Chemical shifts for ^{1}H and ^{13}C NMR are reported in ppm with residual solvent as reference: Acetonitrile (1.94 ppm for ^{1}H, 1.32 ppm for ^{13}C). Abbreviations for signal multiplicity of ^{1}H NMR spectra are shown as following: s: singlet, d: doublet, t: triplet, dd: doublet of doublets; dt: doublet of triplets; m: multiplet, br: broad.

Mass spectrometry (MS)

Low-resolution electrospray ionization mass spectrometry (LR ESI-MS) was undertaken on a Waters XevoTQD (cone voltage 5-20 eV; desolvation temperature 307 K; ionization temperature 325 K), infused from a Harvard syringe pump at a rate of 10 $\mu L \cdot min^{-1}$. High-resolution electrospray ionization mass spectrometry (HR ESI-MS) was undertaken on a Waters Synapt G2-Si mass spectrometer.

DFT calculations

Geometries of cages were first optimized using the semi-empirical method PM6 with Grimme-D3 type dispersion correction. To achieve higher accuracy, the geometries were further optimized using density functional theory (DFT). Density functional B3LYP[2] was employed. Los Alamos Effective Core Potential (LANL2DZ)[3] was used for the metal ion, Zn^{II}, while a Pople type basis set, 6-31G(d)[4,5] was adopted for all other atoms. All quantum mechanical calculations were carried out using the Gaussian16 program suite.[6] Geometry optimizations and the subsequent vibrational analyses were performed in the gas phase.

Cavity volume calculations

In order to determine the available void spaces within the cages, Molovol[7] calculations based on the crystal and modelled structures obtained in this study were performed. A virtual probe with a radius of 1.4 Å was employed to **1**, **1**·$2F^{-}$, and a previous reported[8] trigonal prism during the calculations.

2. Construction and characterization of coordination capsules

2.1 Construction of capsule 1

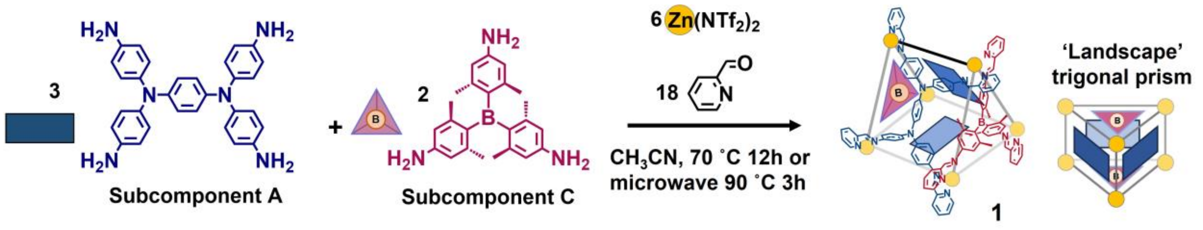

Scheme S1. Subcomponent self-assembly of **1**.

Subcomponent **A** (1.9 mg, 1.5 equiv) and Subcomponent **C** (1.0 mg, 1.0 equiv) were added to CD_3CN (0.6 mL) together with $Zn(NTf_2)_2$ (5.1 mg, 3.0 equiv) and 2-formylpyridine (2.6 mg, 9.0 equiv). The reaction mixture was stirred at 70 °C for 12h or in the microwave at 90 °C for 3h. After cooling to room temperature, the solvent was evaporated and diethyl ether was then added. The residue was resuspended and then centrifuged and the diethyl ether decanted. This was repeated three times with fresh diethyl ether. The residue was then dried in vacuo to afford the desired product (**1**) as a dark red solid (9.5 mg, 90 % yield).

Characterization of capsule **1:**

^{1}H NMR (500 MHz, CD_3CN, 298 K) δ (ppm) 8.82 (s, 6H), 8.76 (s, 6H), 8.57 – 8.47 (m, 12H), 8.42 – 8.28 (m, 24H), 8.24 (d, J = 7.8 Hz, 6H), 8.12 (d, J = 7.8 Hz, 6H), 7.98 (dd, J = 7.9, 5.0 Hz, 6H), 7.75 (ddd, J = 22.5, 11.4, 5.6 Hz, 18H), 7.50 (d, J = 5.1 Hz, 6H), 6.97 (s, 18H), 6.83 (s, 6H), 6.78 (d, J = 8.5 Hz, 12H), 6.68 (d, J = 8.1 Hz, 12H), 6.51 (d, J = 8.5 Hz, 12H), 6.37 (d, J = 8.0 Hz, 12H), 5.47 (s, 6H), 1.88 (s, 18H), 1.53 (s, 18H).

^{13}C NMR (126 MHz, CD_3CN, 298 K) δ (ppm) 165.7, 163.4, 159.1, 149.9, 149.7, 149.4, 148.9, 147.9, 147.1, 146.4, 146.1, 144.8, 144.7, 143.9, 143.1, 142.6, 142.3, 142.2, 142.1, 141.1, 139.3, 131.0, 130.9, 130.8, 130.6, 130.5, 130.0, 129.1, 124.3, 123.7, 123.1, 121.0, 120.7, 120.5, 119.9 (q, $^1J_{CF}$ = 320.8 Hz, NTf_2^-), 117.3, 22.6, 22.3.

ESI-MS: m/z = 793.8 [**1**$(NTf_2^-)_5]^{7+}$, 972.8 [**1**$(NTf_2^-)_6]^{6+}$, 1223.3 [**1**$(NTf_2^-)_7]^{5+}$, 1599.2 [**1**$(NTf_2^-)_8]^{4+}$. Calculated results: m/z = 793.9 [**1**$(NTf_2^-)_5]^{7+}$, 972.9 [**1**$(NTf_2^-)_6]^{6+}$, 1223.5 [**1**$(NTf_2^-)_7]^{5+}$, 1599.4 [**1**$(NTf_2^-)_8]^{4+}$.

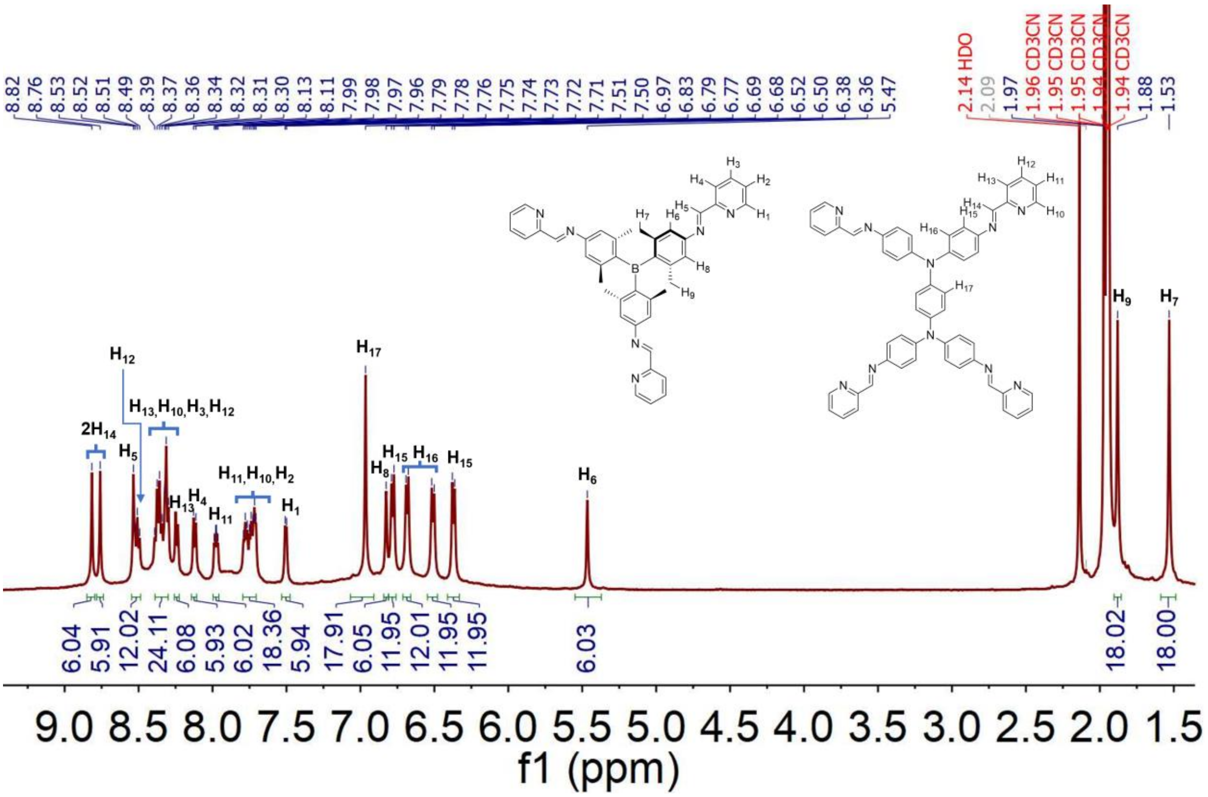

Figure S1. ^{1}H NMR spectrum of **1** (400 MHz, CD_3CN, 298 K).

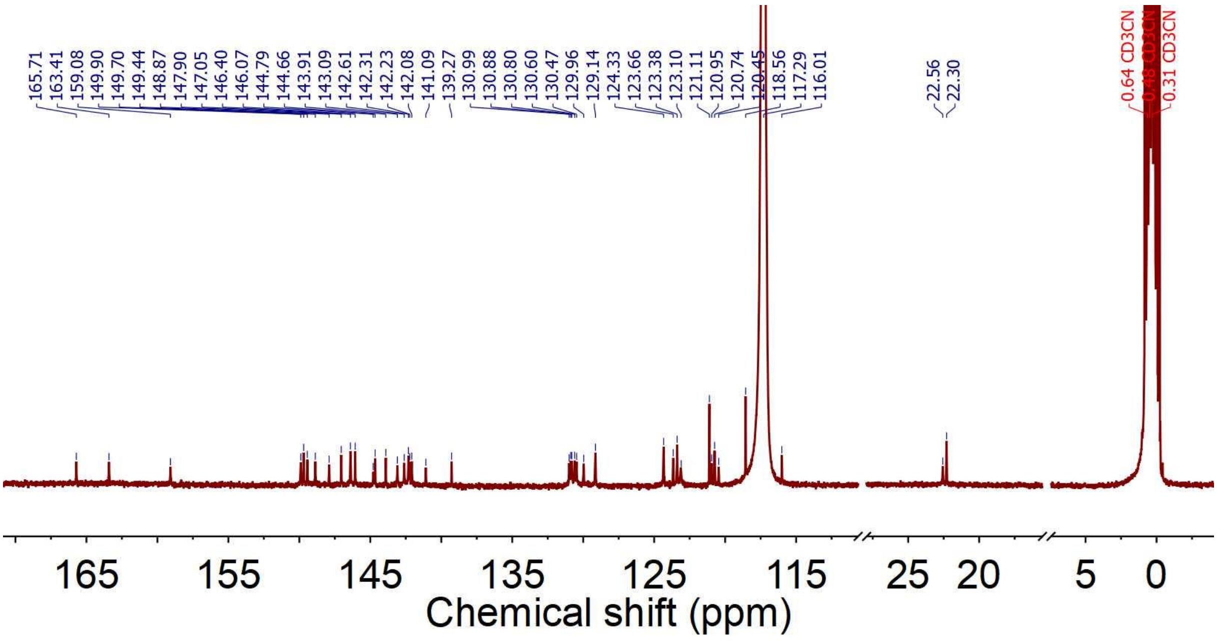

Figure S2. ^{13}C NMR spectrum of **1** (100 MHz, CD_3CN, 298 K).

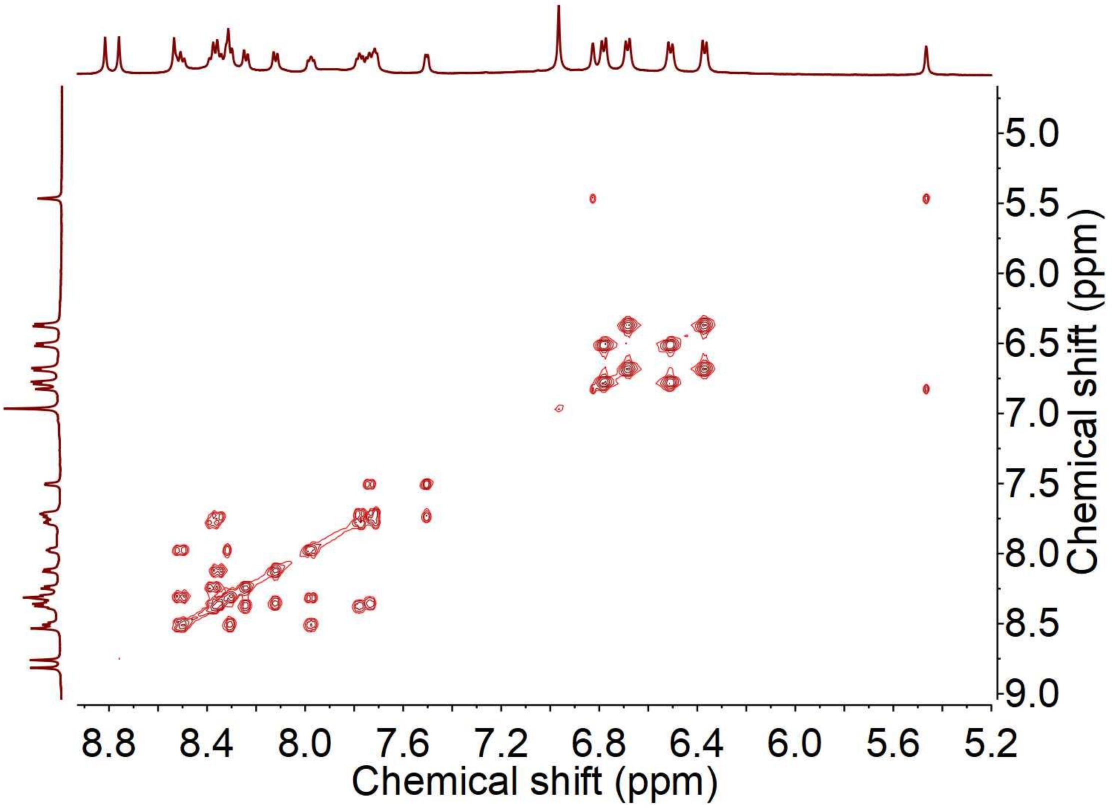

Figure S3. Aromatic region of the ^{1}H-^{1}H COSY NMR spectrum of **1** (500 MHz, CD_3CN, 298 K).

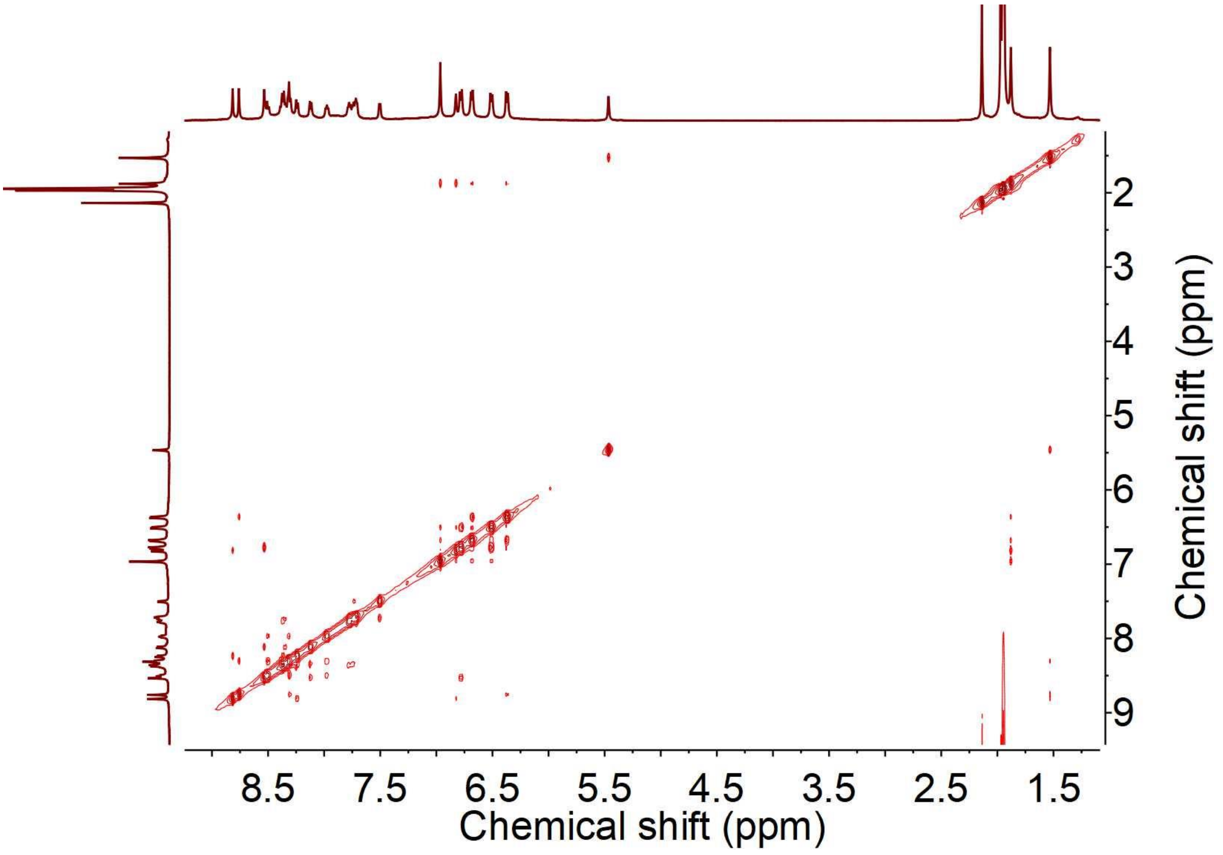

Figure S4. ^{1}H-^{1}H NOESY NMR spectrum of **1** (500 MHz, CD_3CN, 298 K).

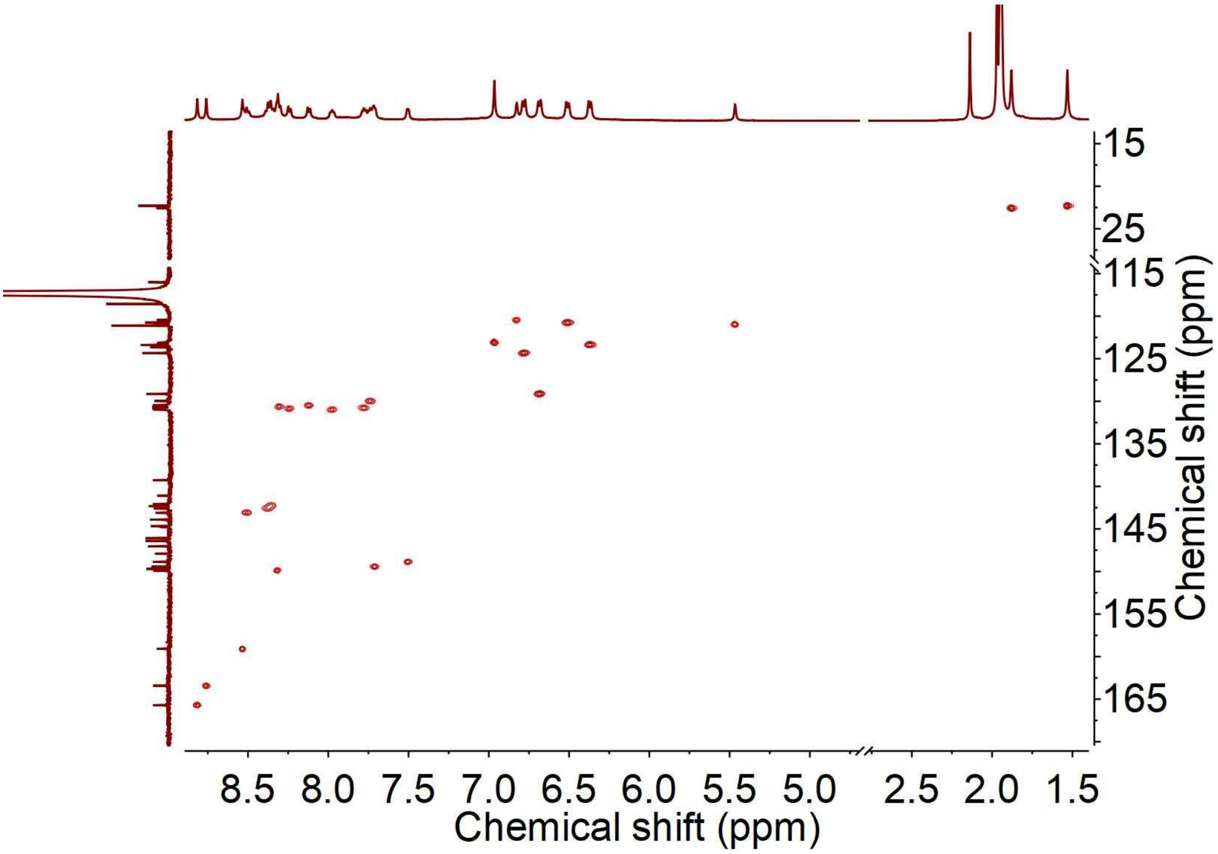

Figure S5. ^{1}H-^{13}C HSQC NMR spectrum of **1** (500 MHz, CD_3CN, 298 K).

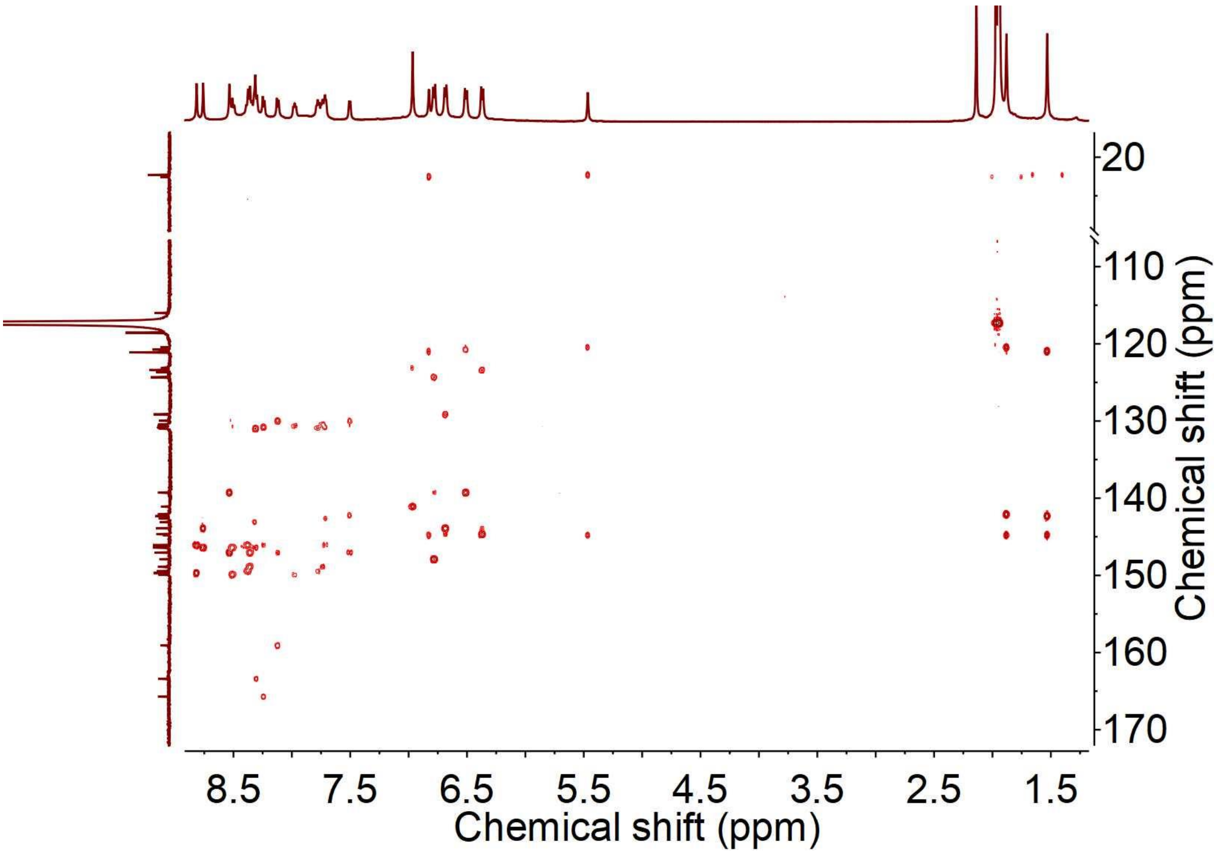

Figure S6. ^{1}H-^{13}C HMBC NMR spectrum of **1** (500 MHz, CD_3CN, 298 K).

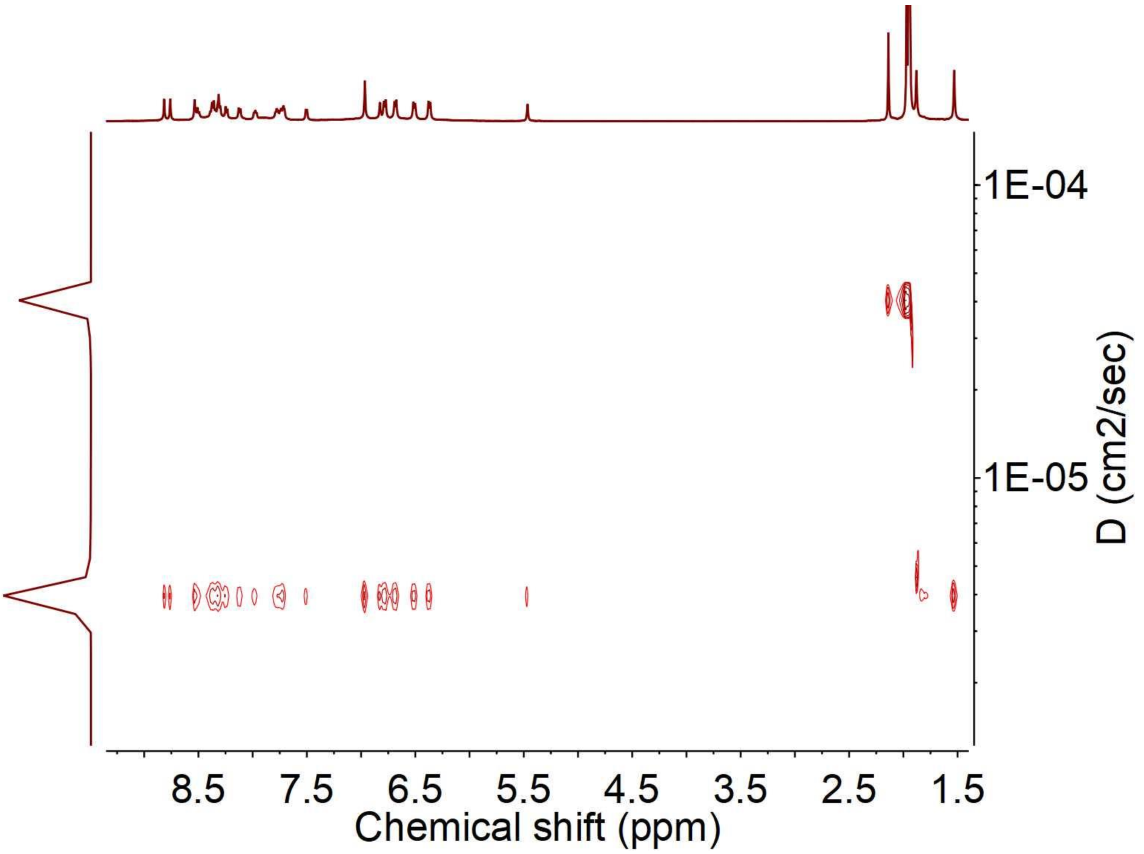

Figure S7. ^{1}H DOSY NMR spectrum of **1** (400 MHz, CD_3CN, 298 K). The diffusion coefficient for **1** in CD_3CN was measured to be 3.93×10^{-6} cm^2 s^{-1}, corresponding to a hydrodynamic radius of 16.6 Å.

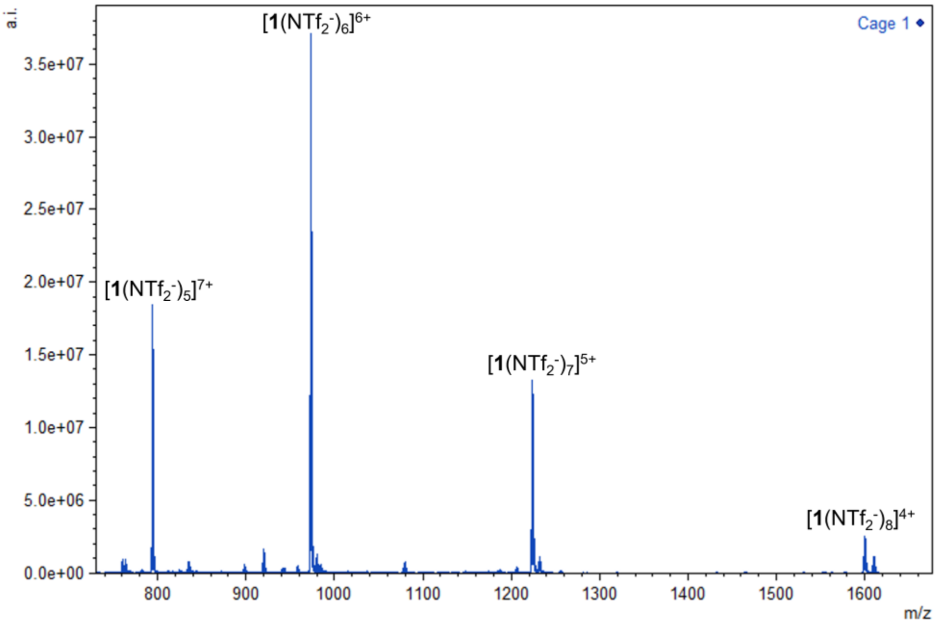

Figure S8. Low-resolution ESI-mass spectrum of **1**$(NTf_2^-)_{12}$. ESI-MS: Experimental results: m/z = 793.8 [**1**$(NTf_2^-)_5]^{7+}$, 972.8 [**1**$(NTf_2^-)_6]^{6+}$, 1223.3 [**1**$(NTf_2^-)_7]^{5+}$, 1599.2 [**1**$(NTf_2^-)_8]^{4+}$. Calculated results: m/z = 793.9 [**1**$(NTf_2^-)_5]^{7+}$, 972.9 [**1**$(NTf_2^-)_6]^{6+}$, 1223.5 [**1**$(NTf_2^-)_7]^{5+}$, 1599.4 [**1**$(NTf_2^-)_8]^{4+}$.

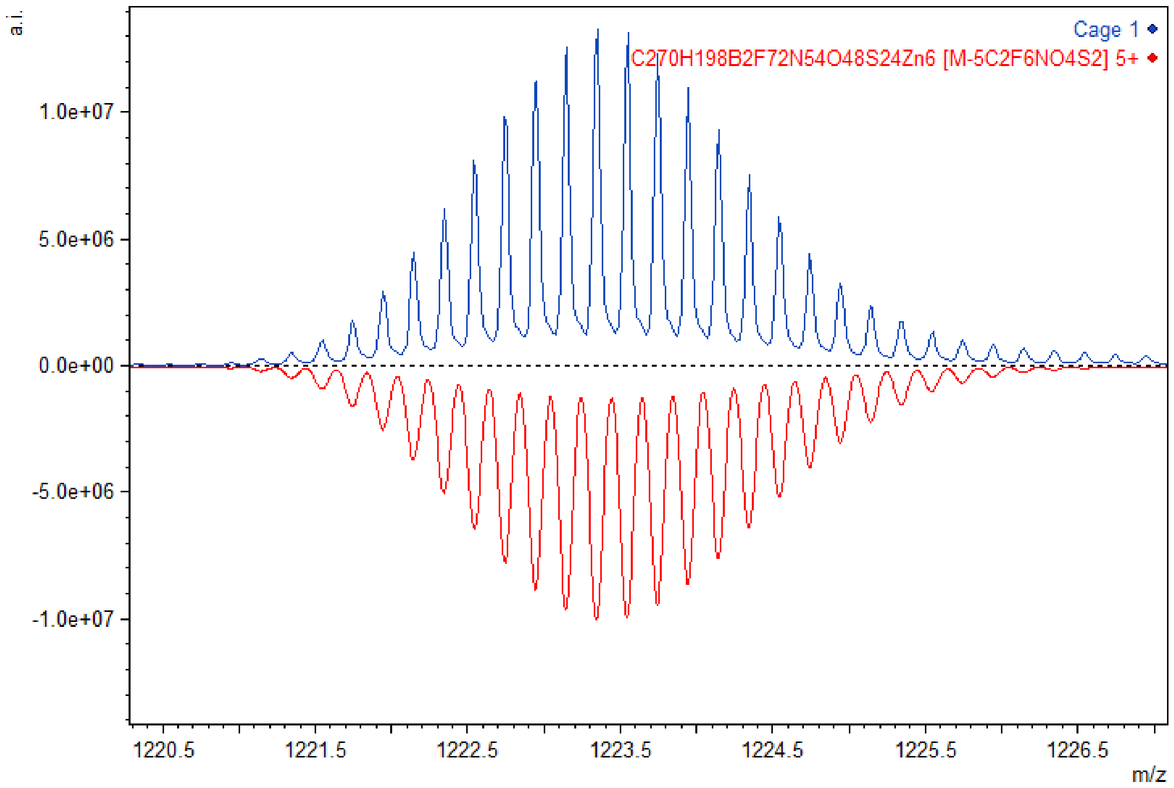

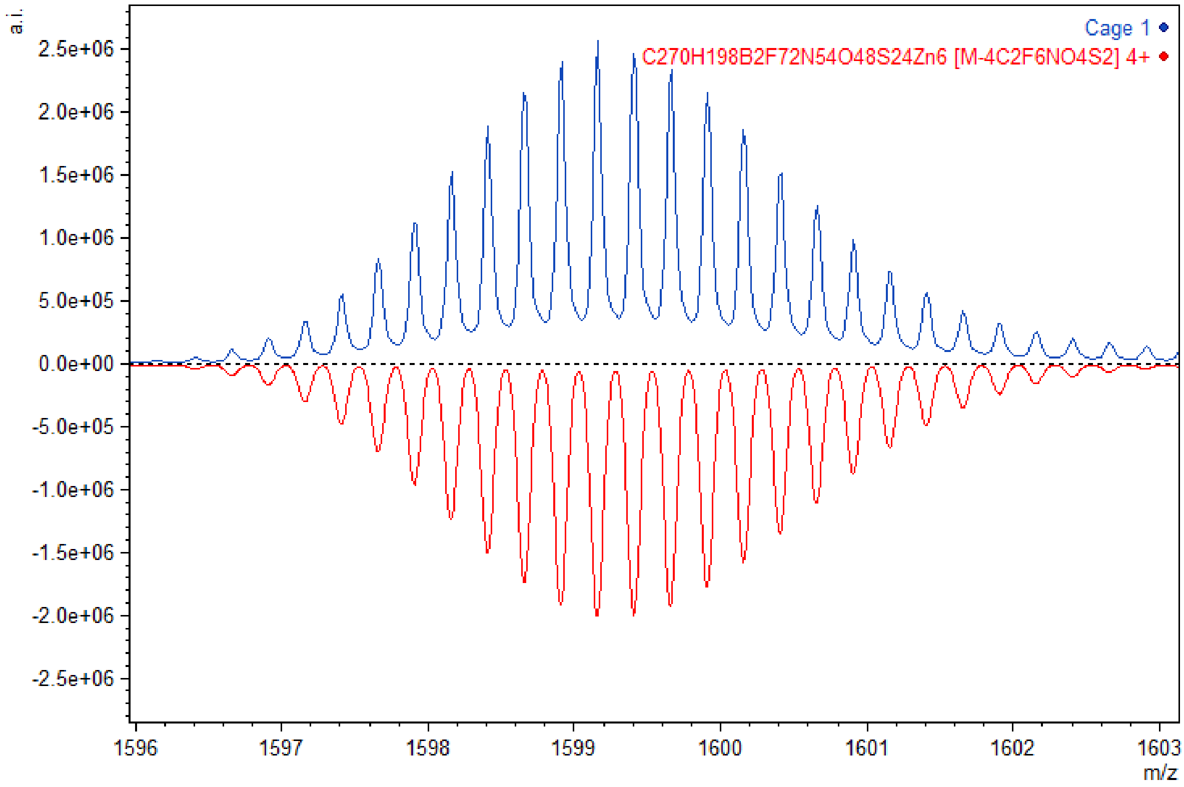

Figure S9. High-resolution ESI-mass spectrometry analysis of **1**·$(NTf_2^-)_{12}$ showing the observed (blue) and theoretical (red) isotope patterns for the +5 and +4 peaks.

2.2 Construction of 1·2F⁻ and intermediate 1·F⁻

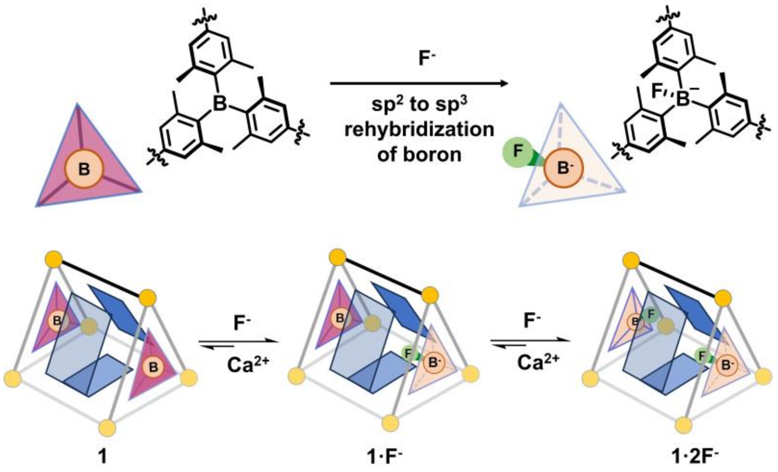

Scheme S2. Construction of cage **1**·2F⁻ and intermediate **1**·F⁻.

The procedure for fluoride addition is adapted from a literature procedure.[1] After stepwise addition of 2.5 equiv of tetrabutylammonium fluoride (TBAF) to **1** in CD_3CN, the color of the solution changed from dark red to light red and a new set of ^{1}H NMR peaks corresponding to the adduct with 2 equiv of F⁻ was observed. This observation indicates that the binding of F⁻ to the boron centers is slow on the ^{1}H NMR time scale. A single set of ligand signals was observed in the ^{1}H NMR spectrum of the product after the addition of 2.5 equiv of TBAF indicating the formation of **1**·2F⁻.

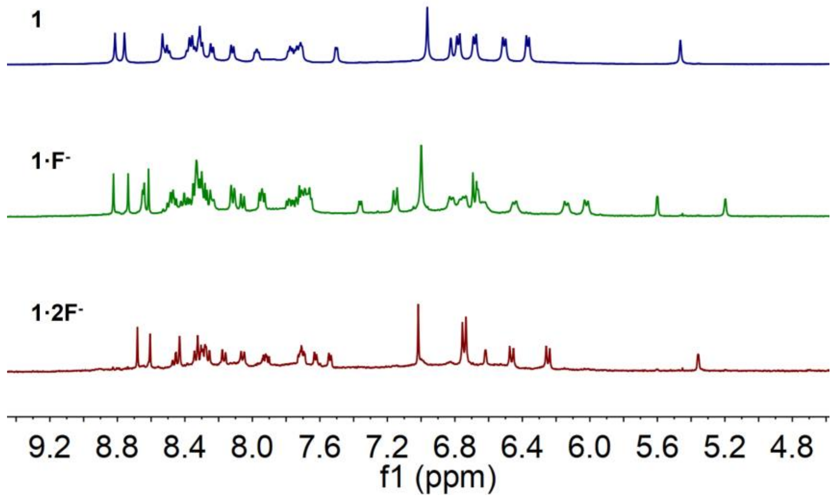

Figure S10. Aromatic region of the ^{1}H NMR spectra (500 MHz, 298 K, CD_3CN) showing the stepwise addition of TBAF to **1** in CD_3CN. A single set of ligand signals was observed in the ^{1}H NMR spectrum after the addition of 2.5 equiv of TBAF, indicating the formation of **1**·2F⁻. During the titration, an intermediate **1**·F⁻ with C_3 symmetry was also observed after adding 1 equiv of TBAF.

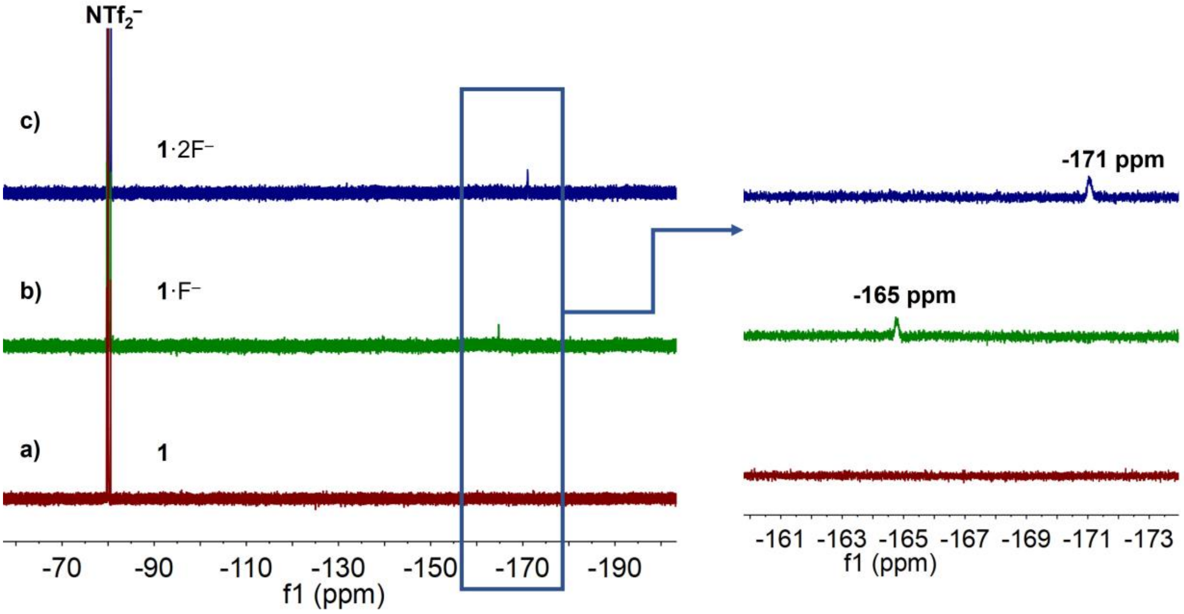

Figure S11. ^{19}F NMR spectra (376 MHz, 298 K, CD_3CN) showing the gradual addition of b) 1 equiv TBAF; c) 2.5 equiv.TBAF to **1** (a) in CD_3CN. No further change in the spectrum was observed after the addition of 2.5 equiv of TBAF, indicating the formation of **1**·2F$^-$, and the ^{19}F signals of B-F$^-$ in **1**·F$^-$ and **1**·2F$^-$ were observed at -165 ppm and -171 ppm, respectively.

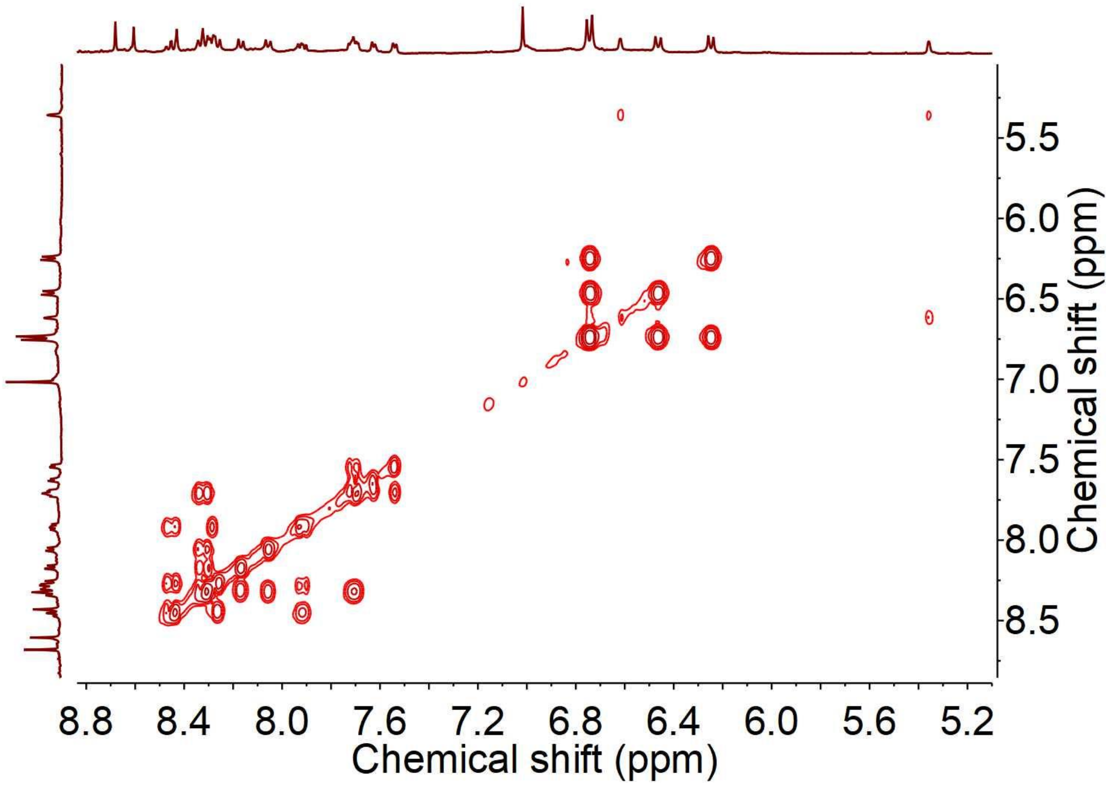

Figure S12. Aromatic region of the ^{1}H-^{1}H COSY NMR spectrum of **1**·2F$^-$ (500 MHz, CD_3CN, 298 K).

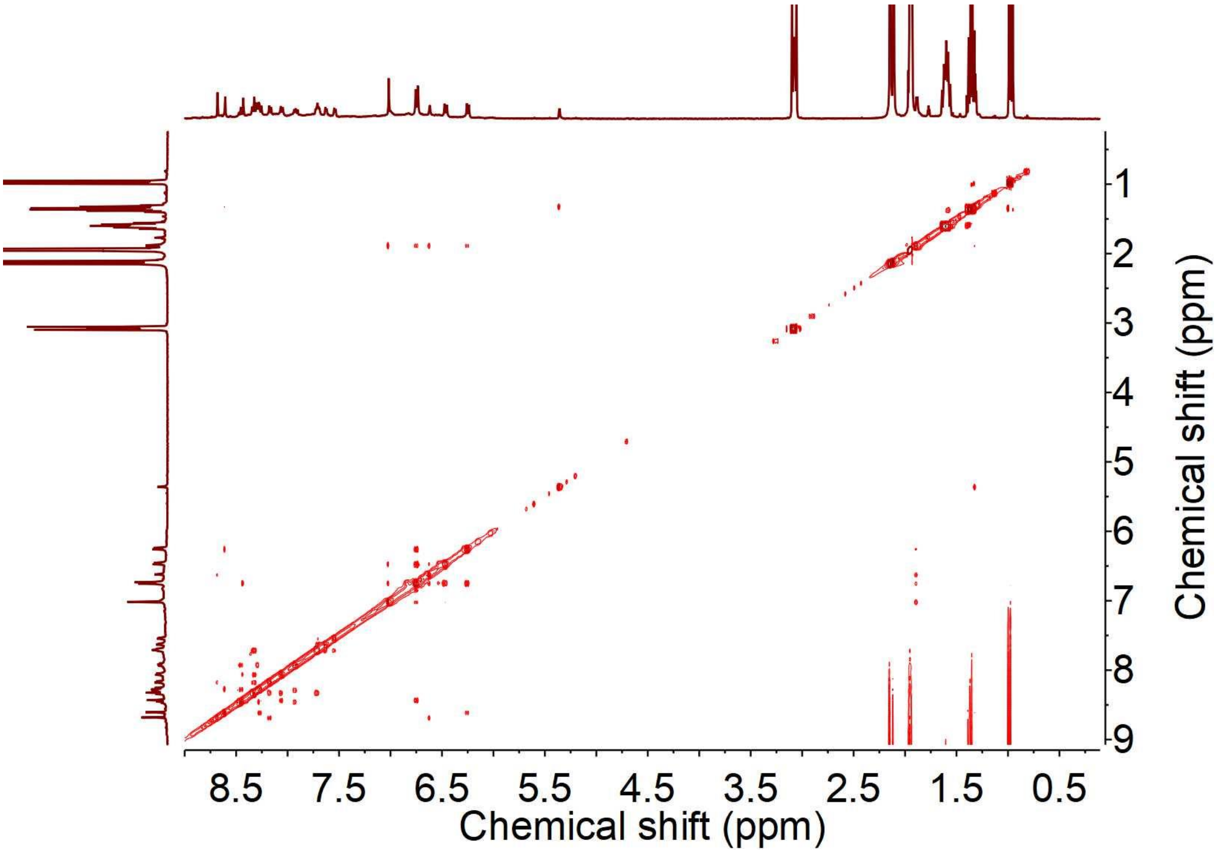

Figure S13. ^1H-^1H NOESY NMR spectrum of cage **1**·$2F^-$ (500 MHz, CD_3CN, 298 K).

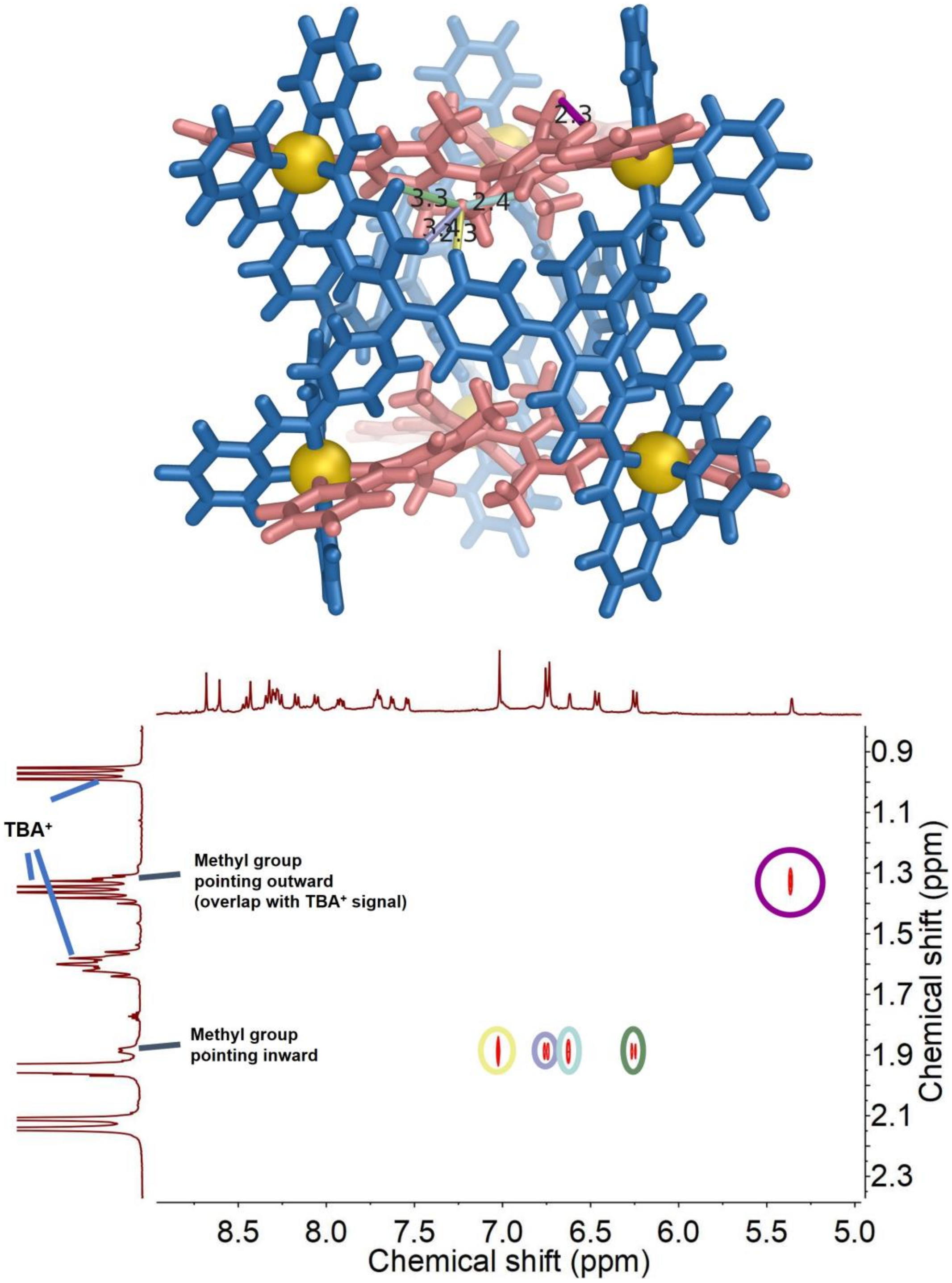

Figure S14. Partial ^1H-^1H NOESY NMR spectrum of cage **1**·$2F^-$ (500 MHz, CD_3CN, 298 K).

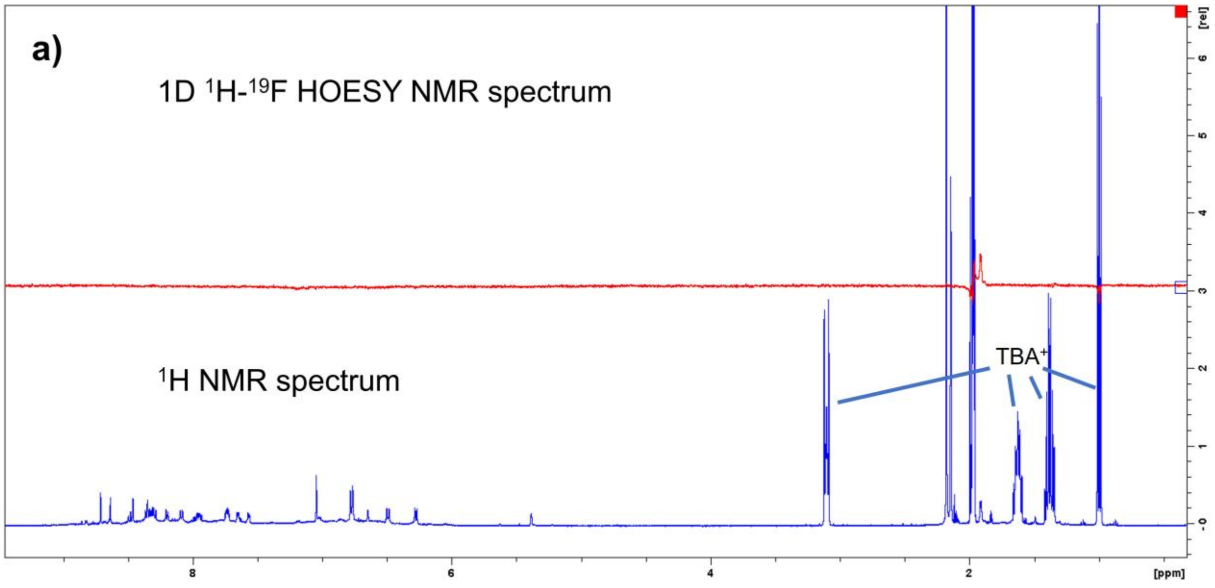

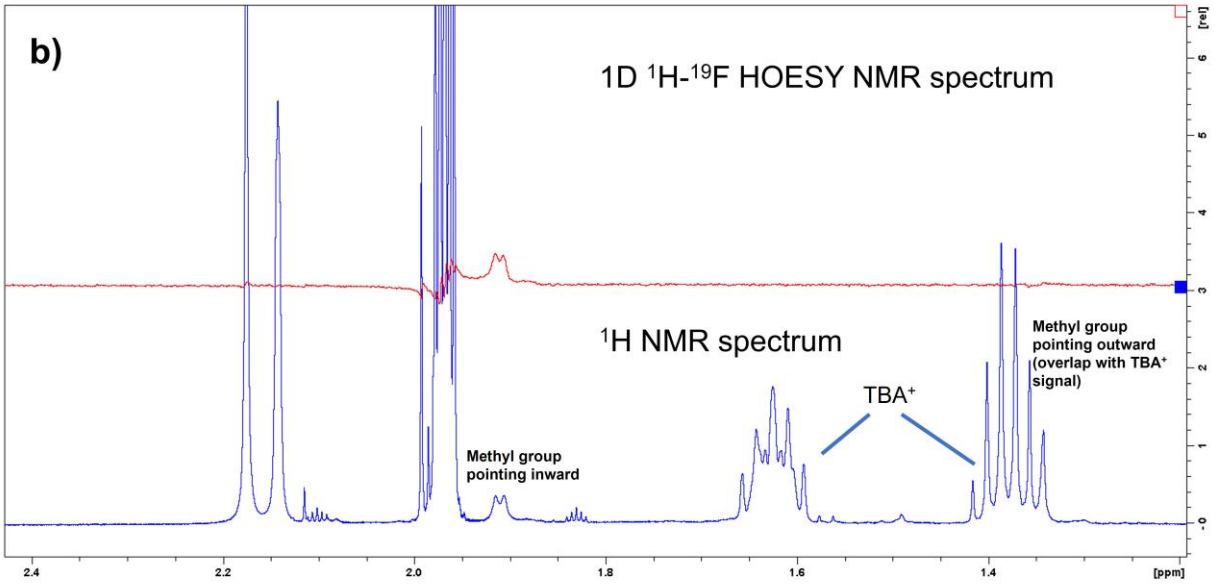

Figure S15. a) 1D ^{1}H-^{19}F HOESY NMR spectrum of **1**·2F^{-} (500 MHz, CD_3CN, 298 K); b) Expanded view of a). Correlation was observed exclusively between the inward-oriented methyl groups and fluoride, indicating inward fluoride binding in **1**·2F^{-}.

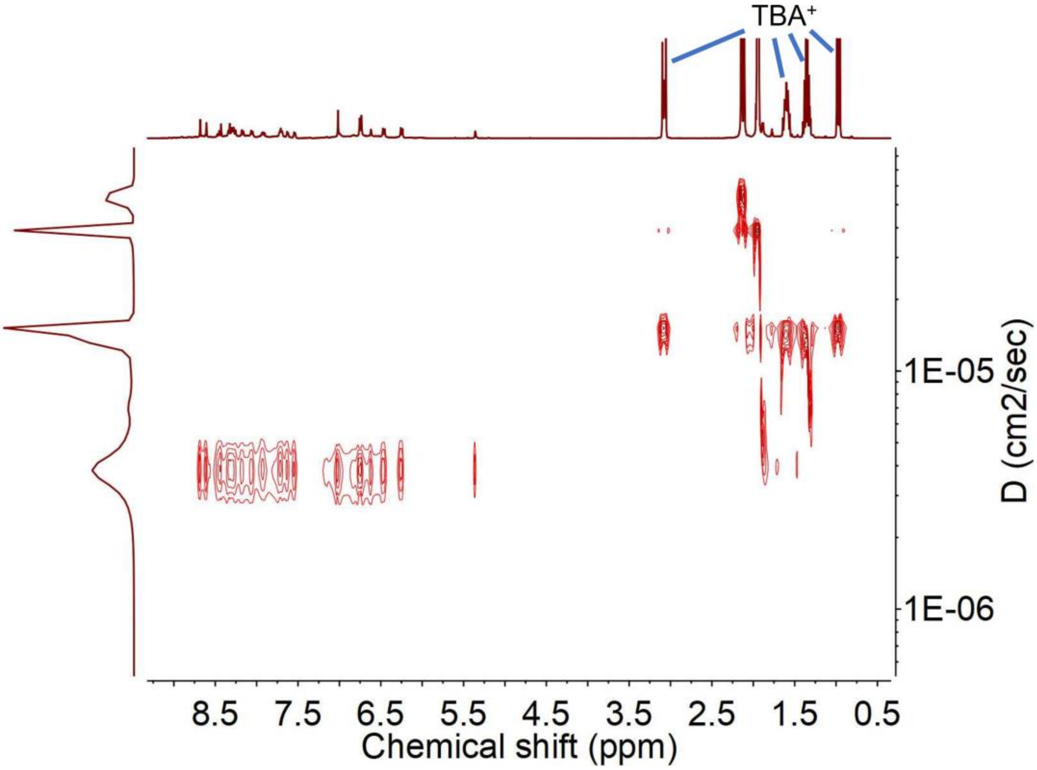

Figure S16 ^{1}H DOSY NMR spectrum of cage **1**·2F⁻ (400 MHz, CD_3CN, 298 K). The diffusion coefficient for **1**·2F⁻ in CD_3CN was measured to be 3.81×10^{-6} cm^2 s^{-1}, corresponding to a hydrodynamic radius of 17.2 Å.

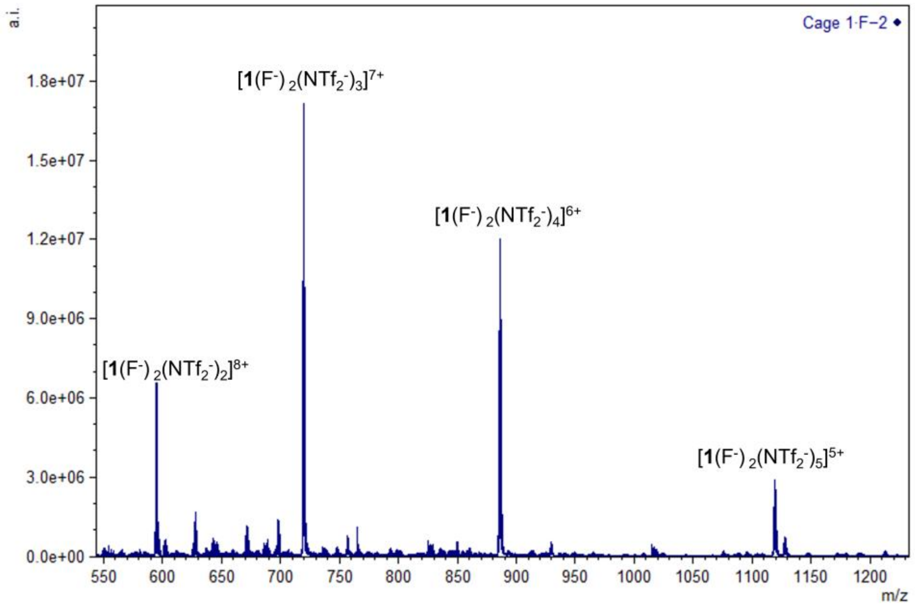

Figure S17. Low-resolution ESI-mass spectrum of **1**$(F^-)_2(NTf_2^-)_{10}$. ESI-MS: Experimental results: m/z = 594.3 [**1**$(F^-)_2(NTf_2^-)_2]^{8+}$, 719.1 [**1**$(F^-)_2(NTf_2^-)_3]^{7+}$, 885.6 [**1**$(F^-)_2(NTf_2^-)_4]^{6+}$, 1119.0 [**1**$(F^-)_2(NTf_2^-)_5]^{5+}$. Calculated results: m/z = 594.3 [**1**$(F^-)_2(NTf_2^-)_2]^{8+}$, 719.3 [**1**$(F^-)_2(NTf_2^-)_3]^{7+}$, 885.8 [**1**$(F^-)_2(NTf_2^-)_4]^{6+}$, 1119.0 [**1**$(F^-)_2(NTf_2^-)_5]^{5+}$.

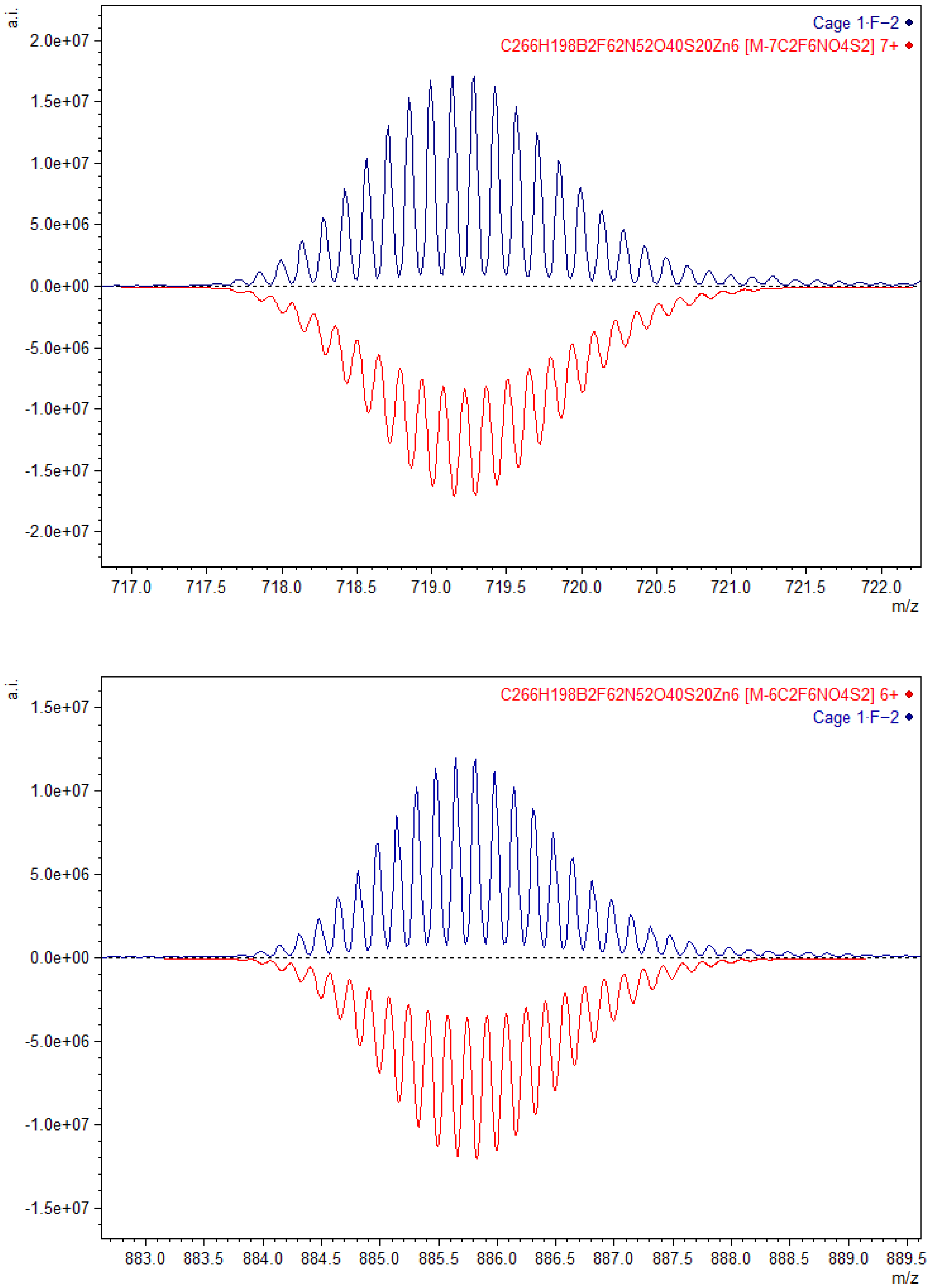

Figure S18. High-resolution ESI-mass spectrometry analysis of **1**$(F^-)_2(NTf_2^-)_{10}$ showing the observed (blue) and theoretical (red) isotope patterns for the +7 and +6 peaks.

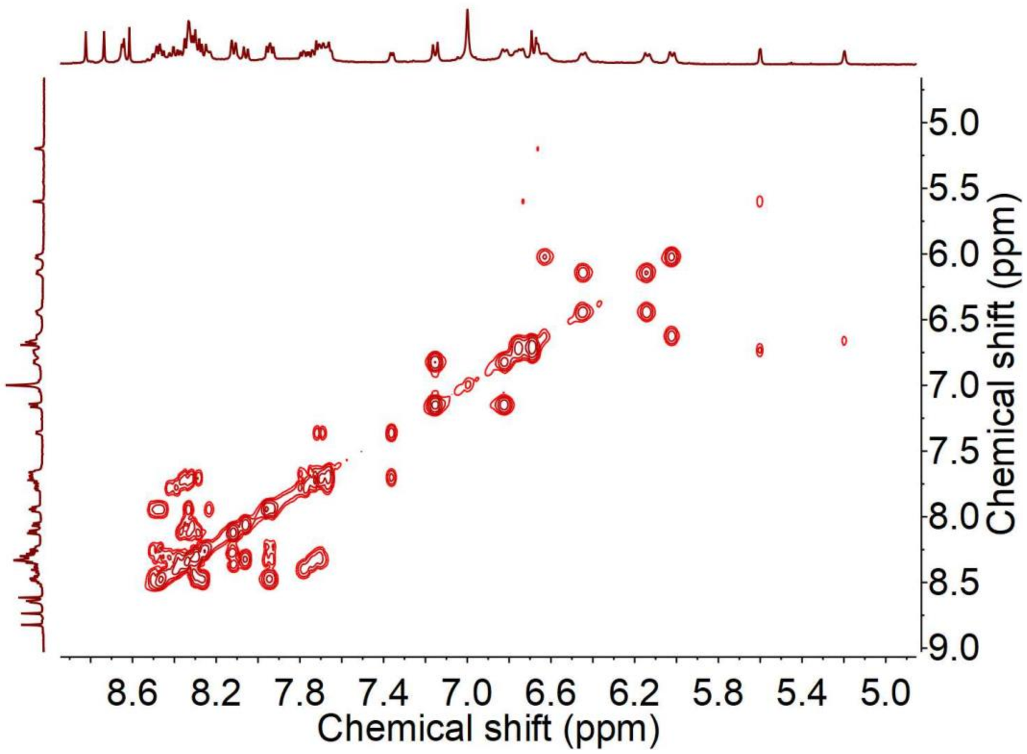

Figure S19. Aromatic region of the ^{1}H-^{1}H COSY NMR spectrum of **1**·F^- (500 MHz, CD_3CN, 298 K).

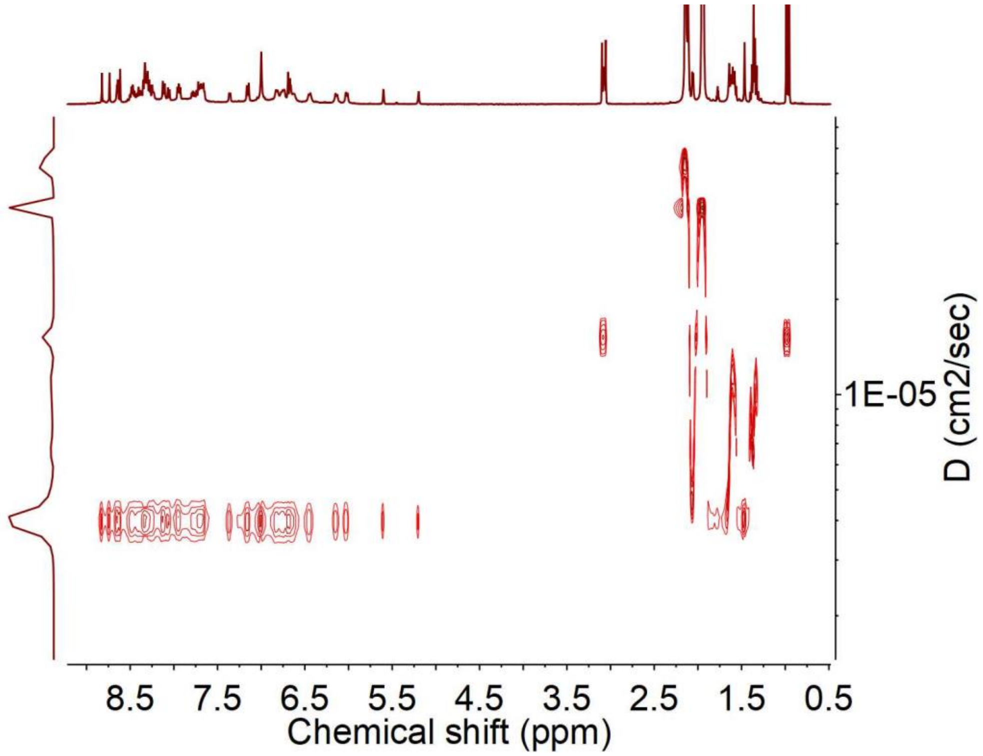

Figure S20. ^{1}H DOSY NMR spectrum of cage **1**·F^- (400 MHz, CD_3CN, 298 K). The diffusion coefficient for **1**·F^- in CD3CN was measured to be 3.96×10^{-6} cm^2 s^{-1}, corresponding to a hydrodynamic radius of 16.5 Å.

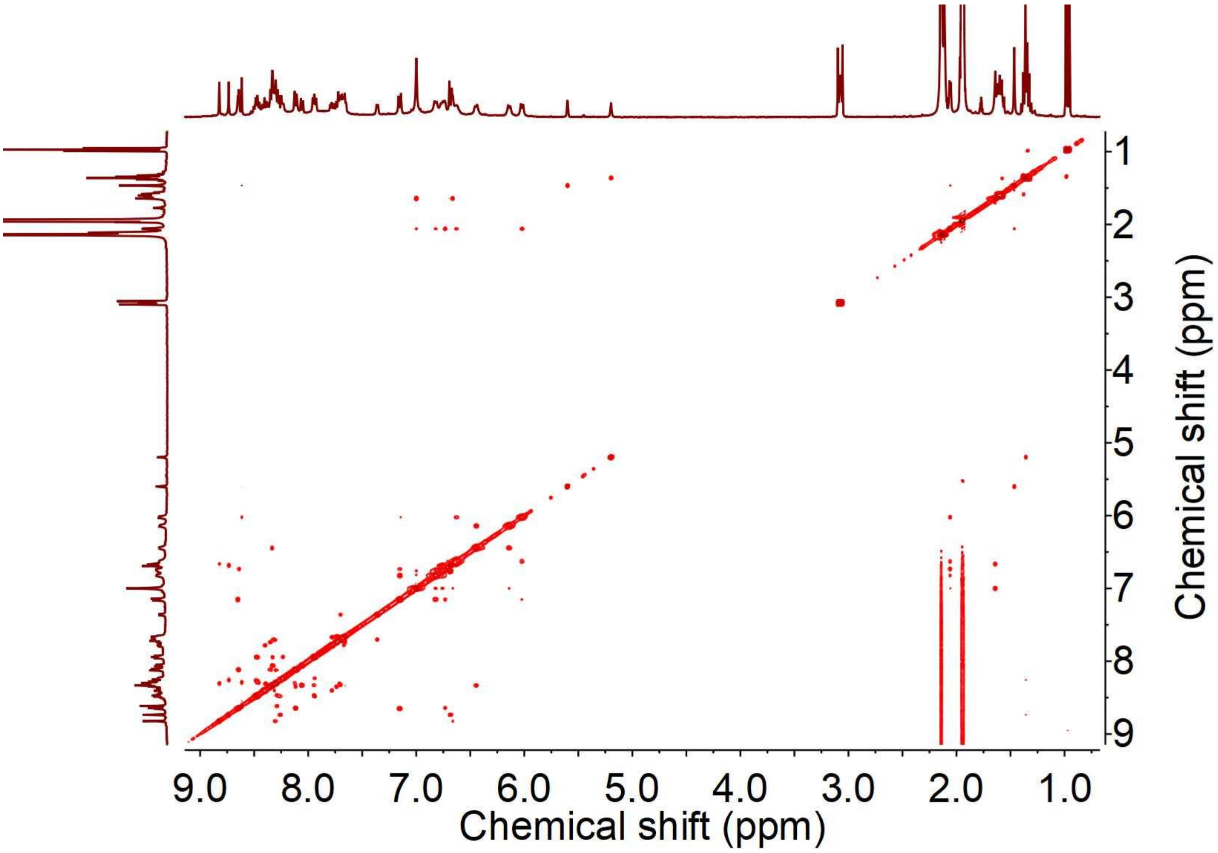

Figure S21. ^{1}H-^{1}H NOESY NMR spectrum of cage **1**·F$^-$ (500 MHz, CD_3CN, 298 K).

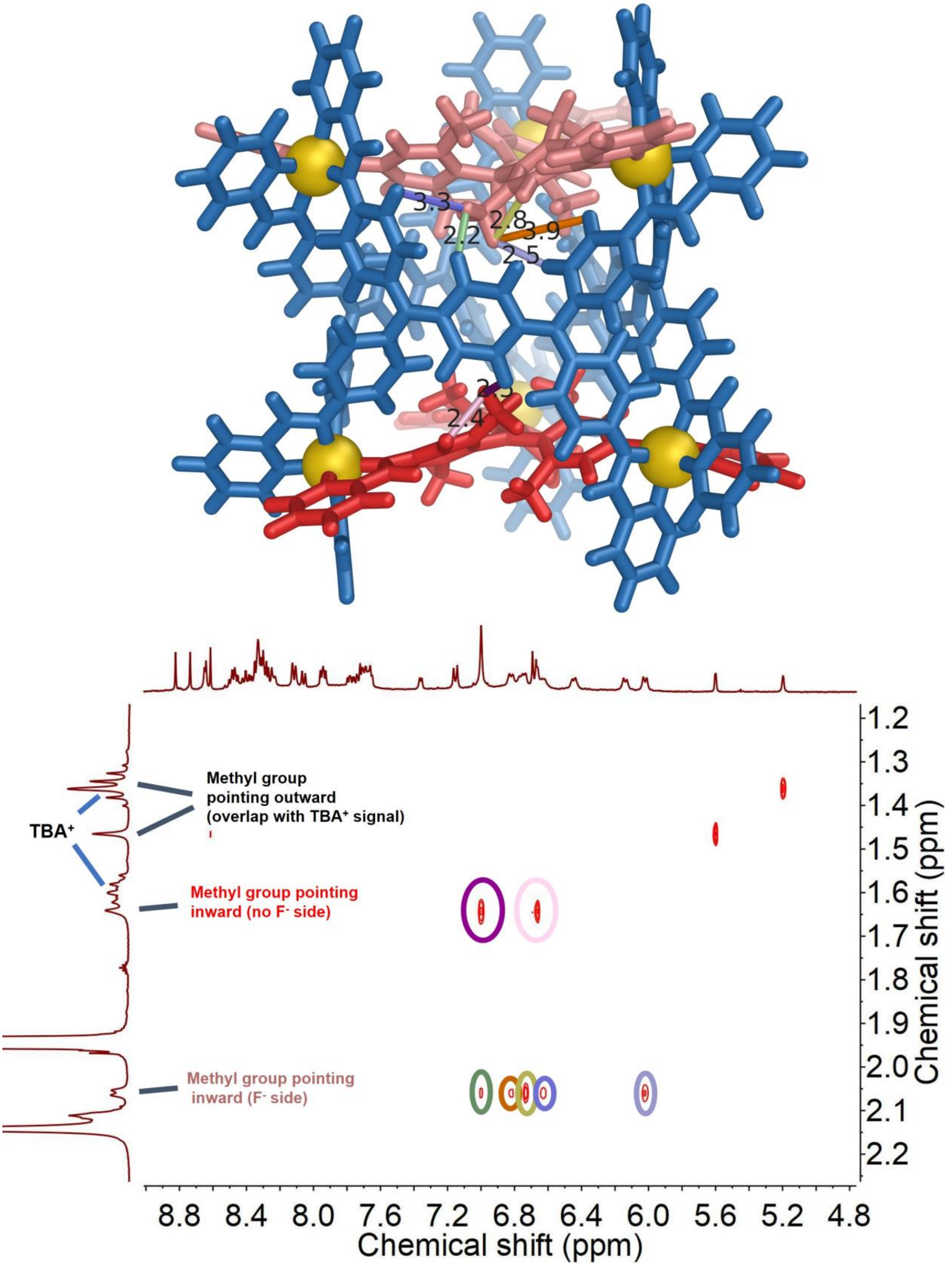

Figure S22. Partial ^{1}H-^{1}H NOESY NMR spectrum of cage **1**·F^{-} (500 MHz, CD_3CN, 298 K).

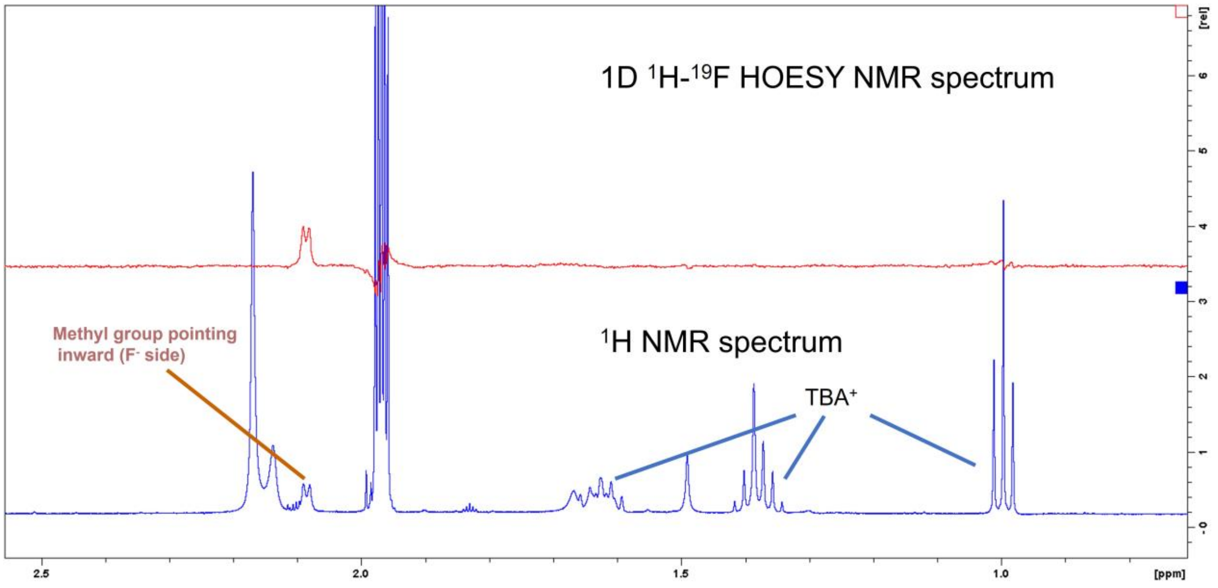

Figure S23. Partial 1D ^{1}H-^{19}F HOESY NMR spectrum of **1**·F$^-$ (500 MHz, CD_3CN, 298 K). Correlation was observed exclusively between an inward-oriented methyl group and fluoride, indicating inward fluoride binding in **1**·F$^-$.

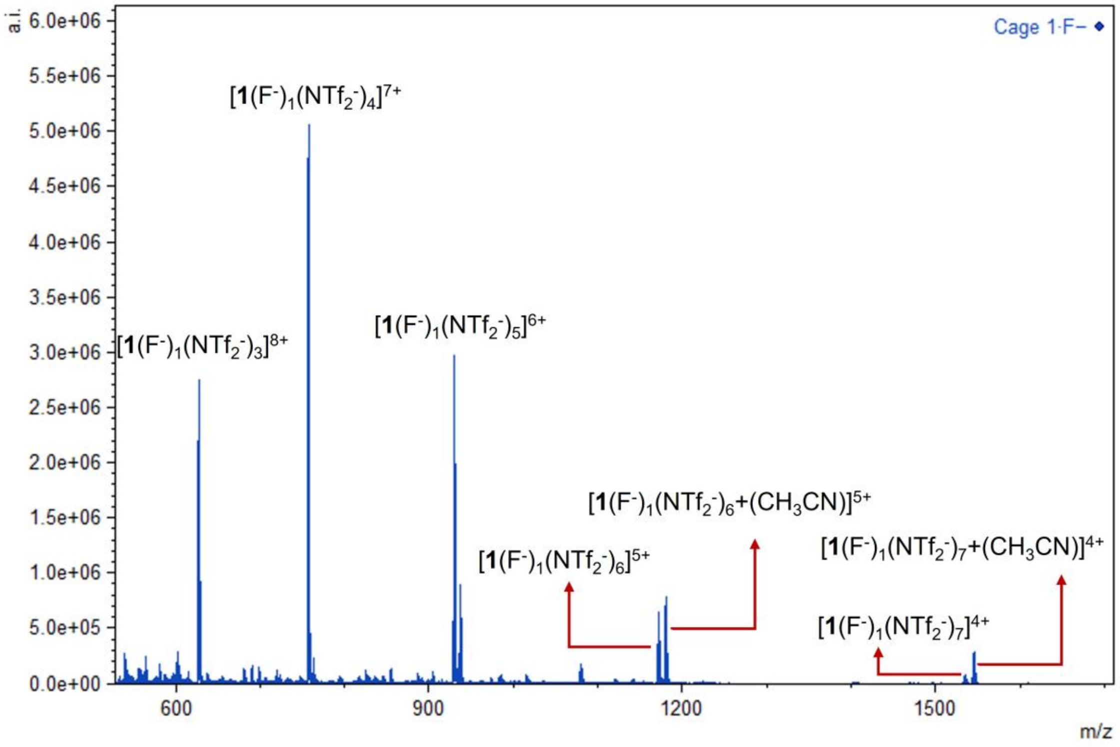

Figure S24. Low-resolution ESI-mass spectrum of **1**$(F^-)_1(NTf_2^-)_{11}$. ESI-MS: Experimental results: m/z = 627.1 [**1**$(F^-)_1(NTf_2^-)_2]^{8+}$, 756.6 [**1**$(F^-)_1(NTf_2^-)_4]^{7+}$, 929.3 [**1**$(F^-)_1(NTf_2^-)_5]^{6+}$, 1171.4 [**1**$(F^-)_1(NTf_2^-)_6]^{5+}$, 1533.9 [**1**$(F^-)_1(NTf_2^-)_7]^{4+}$. Calculated results: m/z = 627.0 [**1**$(F^-)_1(NTf_2^-)_2]^{8+}$, 756.6 [**1**$(F^-)_1(NTf_2^-)_4]^{7+}$, 929.4 [**1**$(F^-)_1(NTf_2^-)_5]^{6+}$, 1171.3 [**1**$(F^-)_1(NTf_2^-)_6]^{5+}$, 1534.1 [**1**$(F^-)_1(NTf_2^-)_7]^{4+}$.

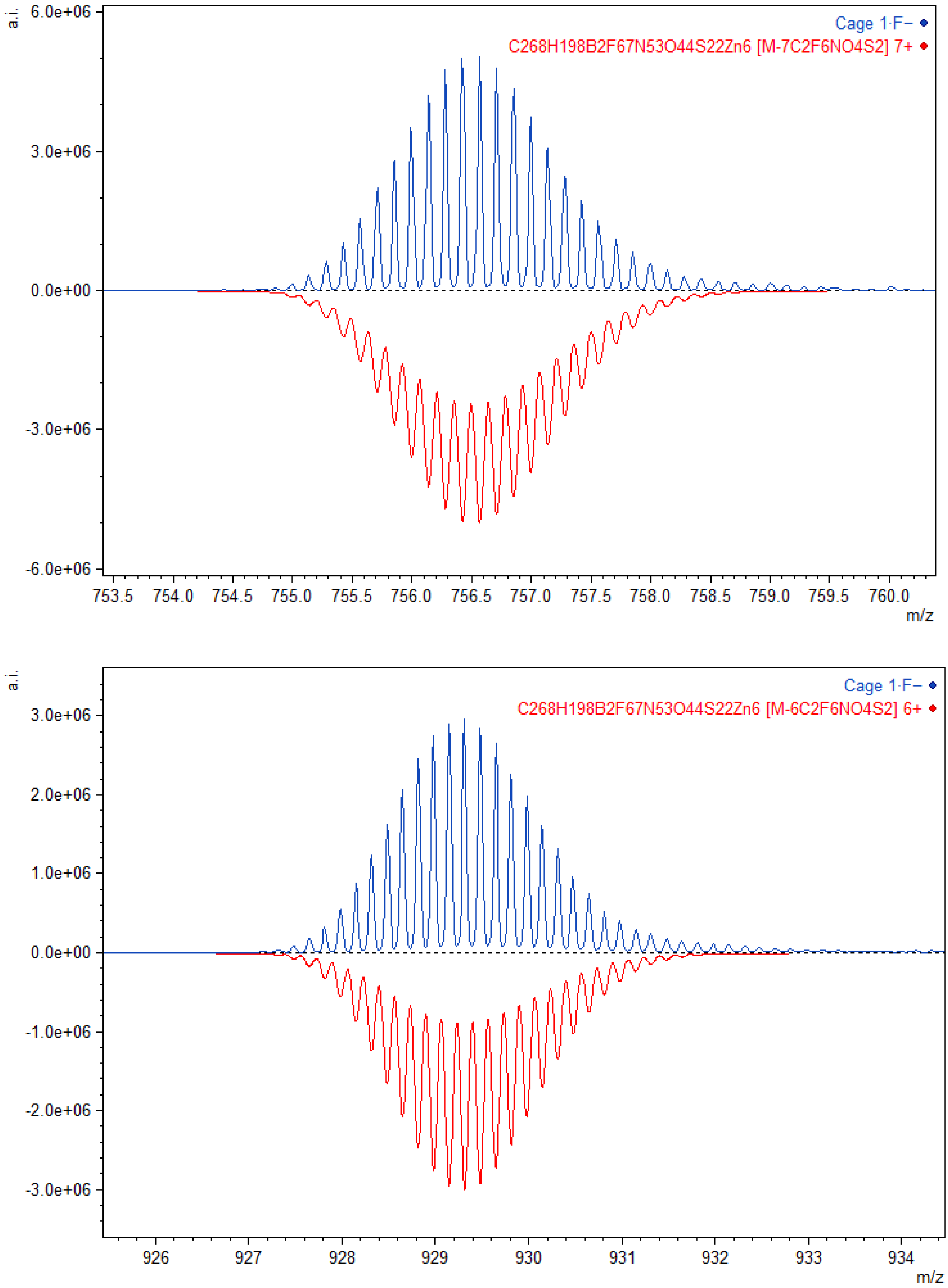

Figure S25. High-resolution ESI-mass spectrometry analysis of **1**$\cdot(F^-)_1(NTf_2^-)_{11}$ showing the observed (blue) and theoretical (red) isotope patterns for the +7 and +6 peaks.

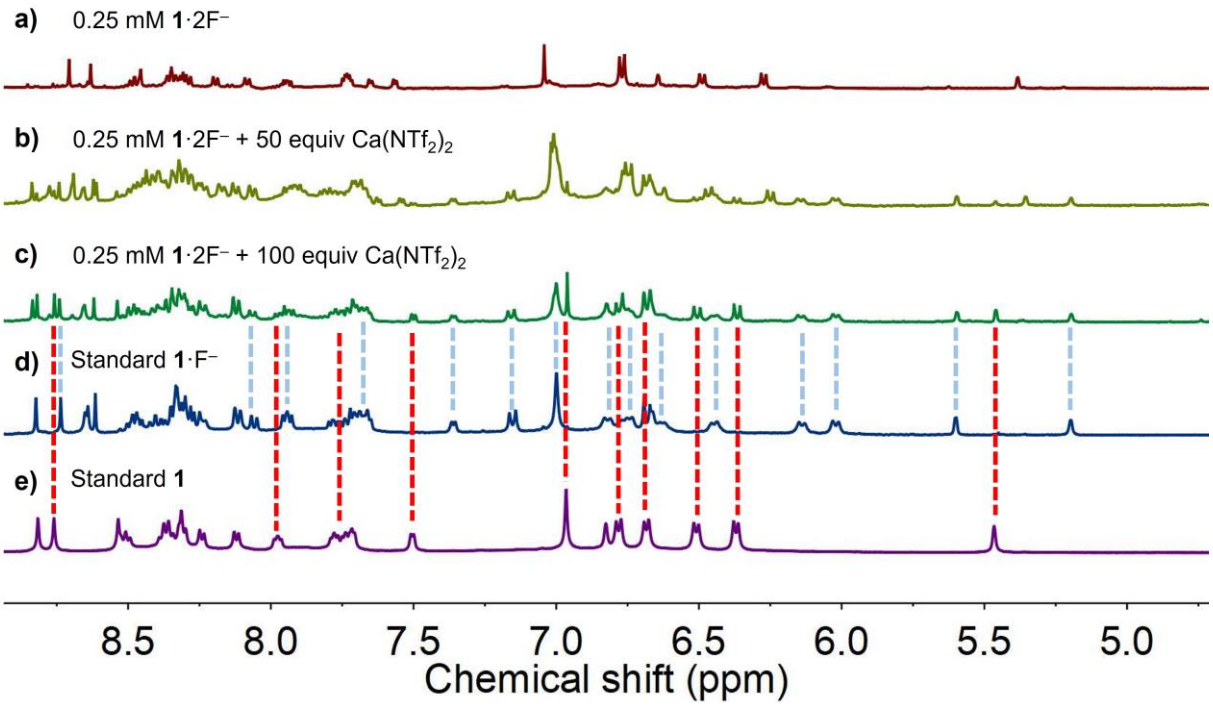

Figure S26. Aromatic region of ^{1}H NMR spectra (400 MHz, 298 K, CD_3CN) showing the gradual addition of $Ca(NTf_2)_2$ to 0.25 mM **1**·$2F^-$ in CD_3CN. a) 0.25 mM **1**·$2F^-$; b) 0.25 mM **1**·$2F^-$ with addition of 50 equiv $Ca(NTf_2)_2$; c) 0.25 mM **1**·$2F^-$ with addition of 100 equiv $Ca(NTf_2)_2$; d) Pure sample of 0.5 mM **1**·F^- ; e) Pure sample of 0.5 mM **1**.

3. Host-guest studies

3.1 General procedures

Host-guest complexes were prepared on an NMR scale and characterized by ^{1}H NMR spectroscopy. A solution of cage sample in CD_3CN (0.25 mM) was transferred to an NMR tube and guest molecules were gradually added. The NMR tube was heated at 50 °C for 10 h after guest addition, and then the ^{1}H NMR spectrum and ESI-MS spectrum were measured to confirm guest encapsulation.

Calculation of binding constants: NMR spectra were measured after cooling down to room temperature for 1 hour. The peaks of empty host **1** and **1**·$2F^-$, guests **G1-G8** and the host-guest complexes (**G1-G8⊂1** and **G1-G8⊂1**·$2F^-$) were well separated in the ^{1}H NMR spectra enabling the peaks for the different species to be integrated. The peaks used for concentration determination are labelled in the following figures. Binding constants were calculated with different amounts of guest present and averaged. The averaged binding constants were calculated via formula Ka = [HG]/[H][G]. The heating of **1**·F^- led to the generation of a small amount of both **1** and **1**·$2F^-$; therefore, the binding affinity between guests and the host **1**·F^- was not investigated.

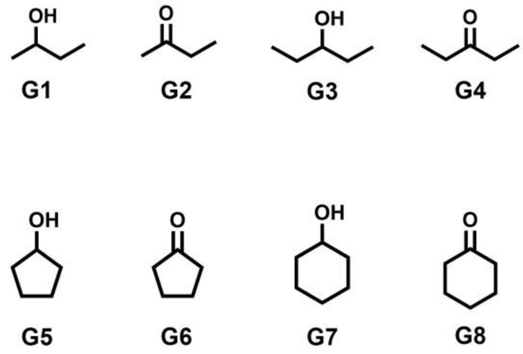

Figure S27. List of guests **G1-G8**.

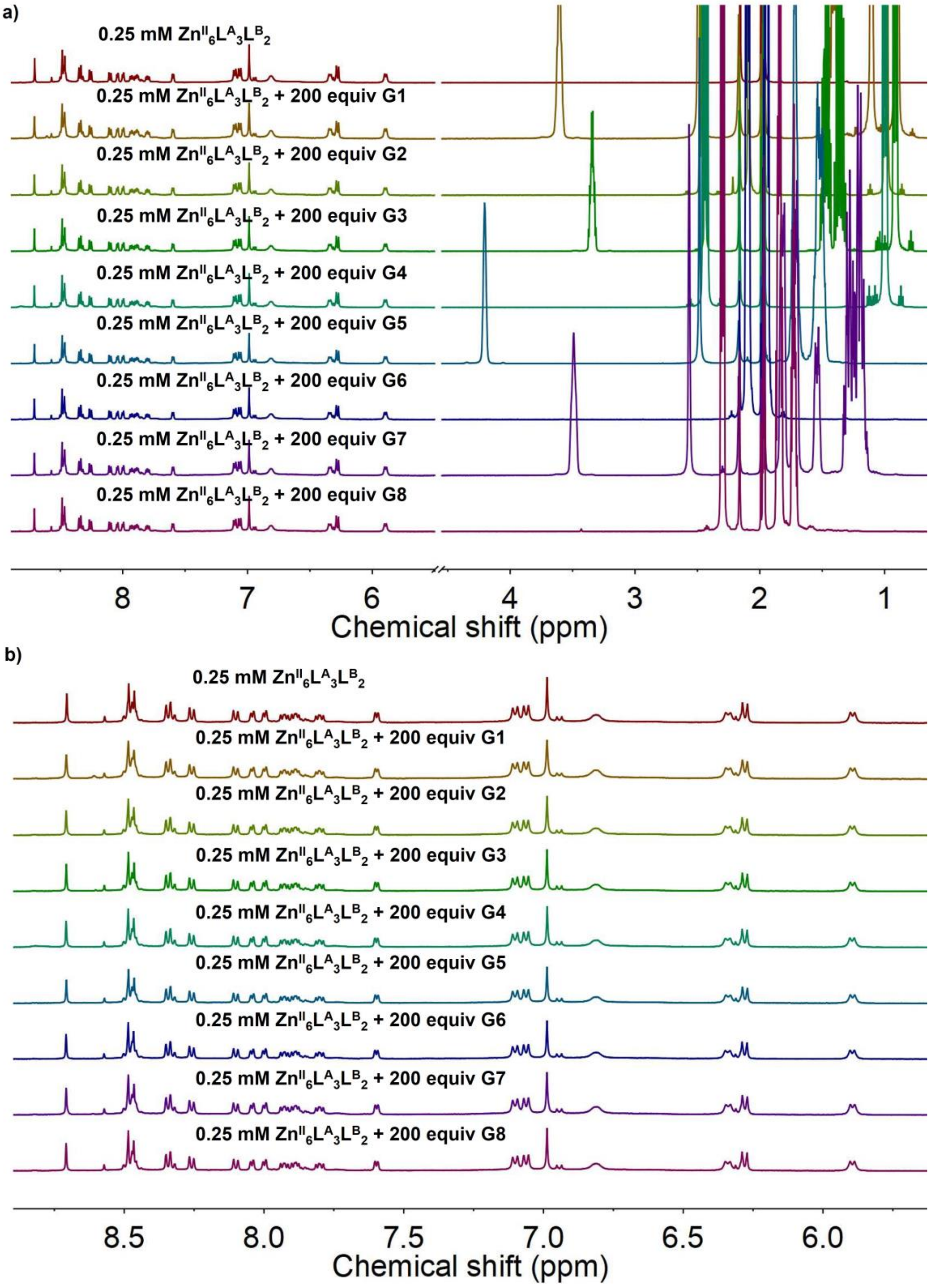

Figure S28. ^{1}H NMR spectra (500 MHz, CD_3CN, 298 K) of a) 0.25 mM previously-reported trigonal prismatic ‘portrait’ capsule with 200 equiv of **G1-G8**; b) Expanded view of the aromatic region of a). No significant binding interactions were observed based on the signals of the central phenyl moieties (6.98 ppm).

3.2 Host-guest interactions of 1 with different guest molecules (G1-G8)

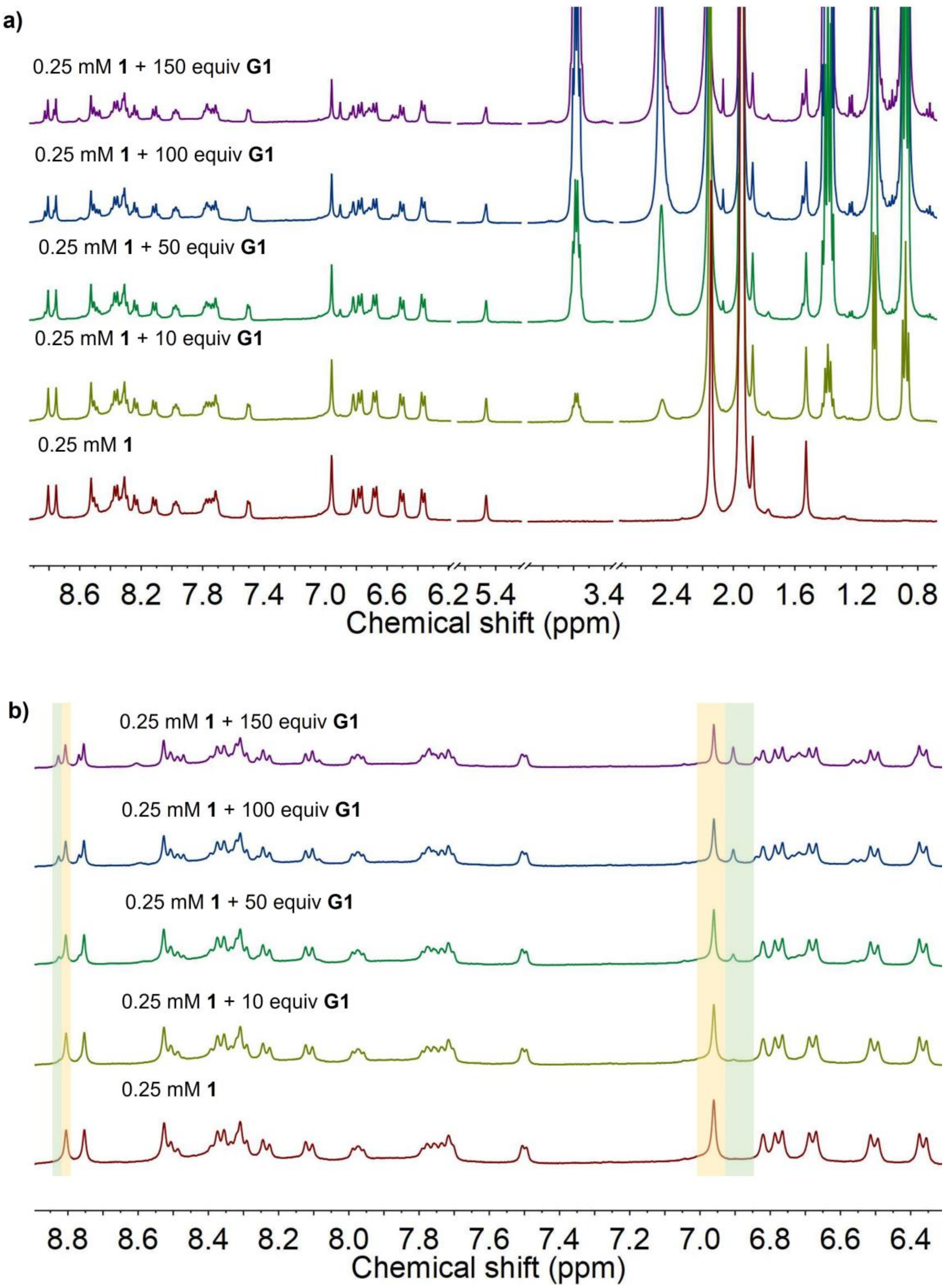

Figure S29. ^{1}H NMR spectra (500 MHz, CD_3CN, 298 K) of a) 0.25 mM **1** with different amounts of **G1** (2-butanol, 10 equiv, 50 equiv, 100 equiv, 150 equiv) ; b) Expanded view of the aromatic region of a). Selected peaks for the **G1⊂1** and free **1** are highlighted by light green and light yellow shading respectively. The binding constants was calculated to be 32.1 ± 6.1 M^{-1}.

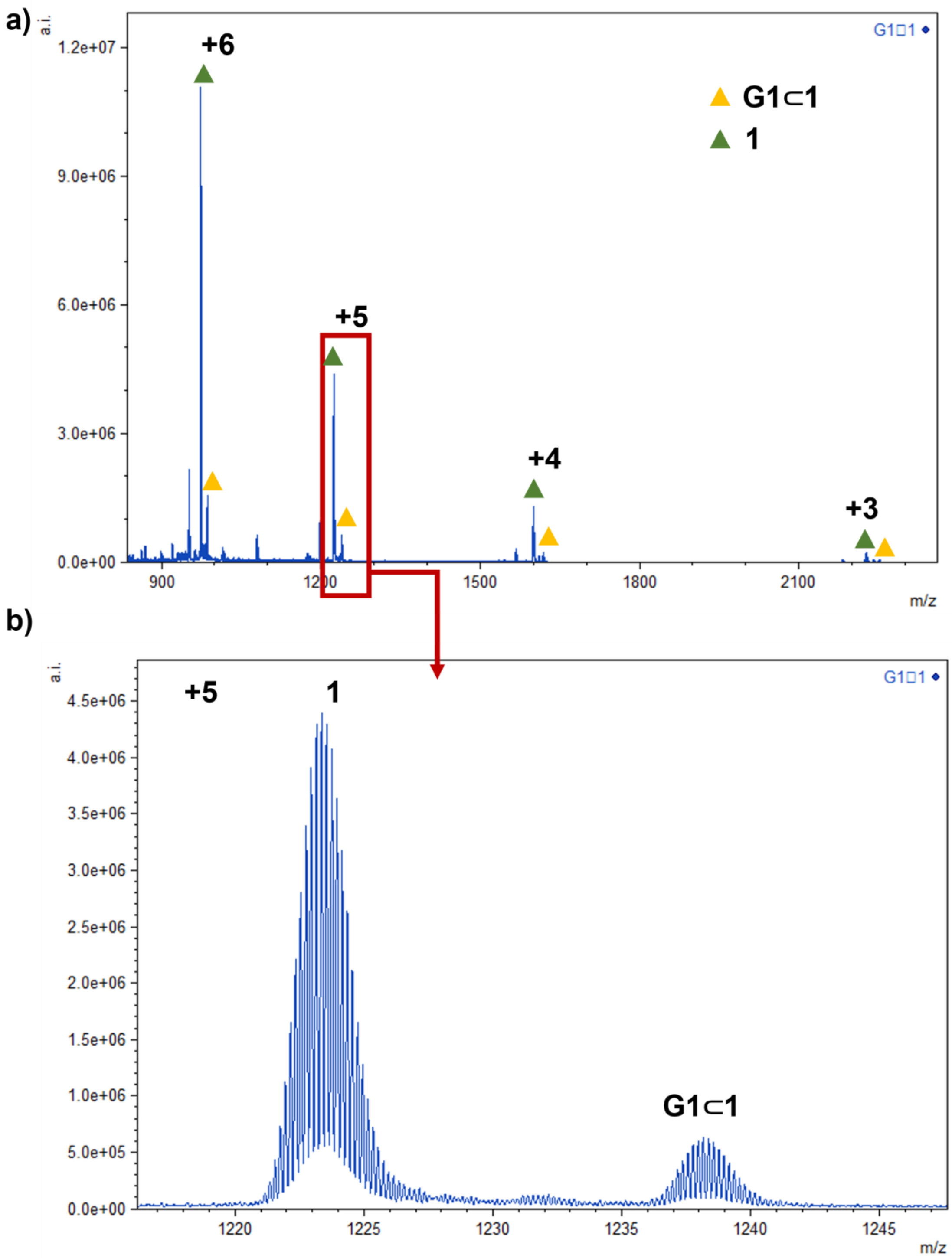

Figure S30. a) High-resolution ESI-mass spectrometry analysis of **1** mixed with 150 equiv **G1**, showing the +6, +5, +4 and +3 peaks ; b) Expanded view showing the +5 peaks.

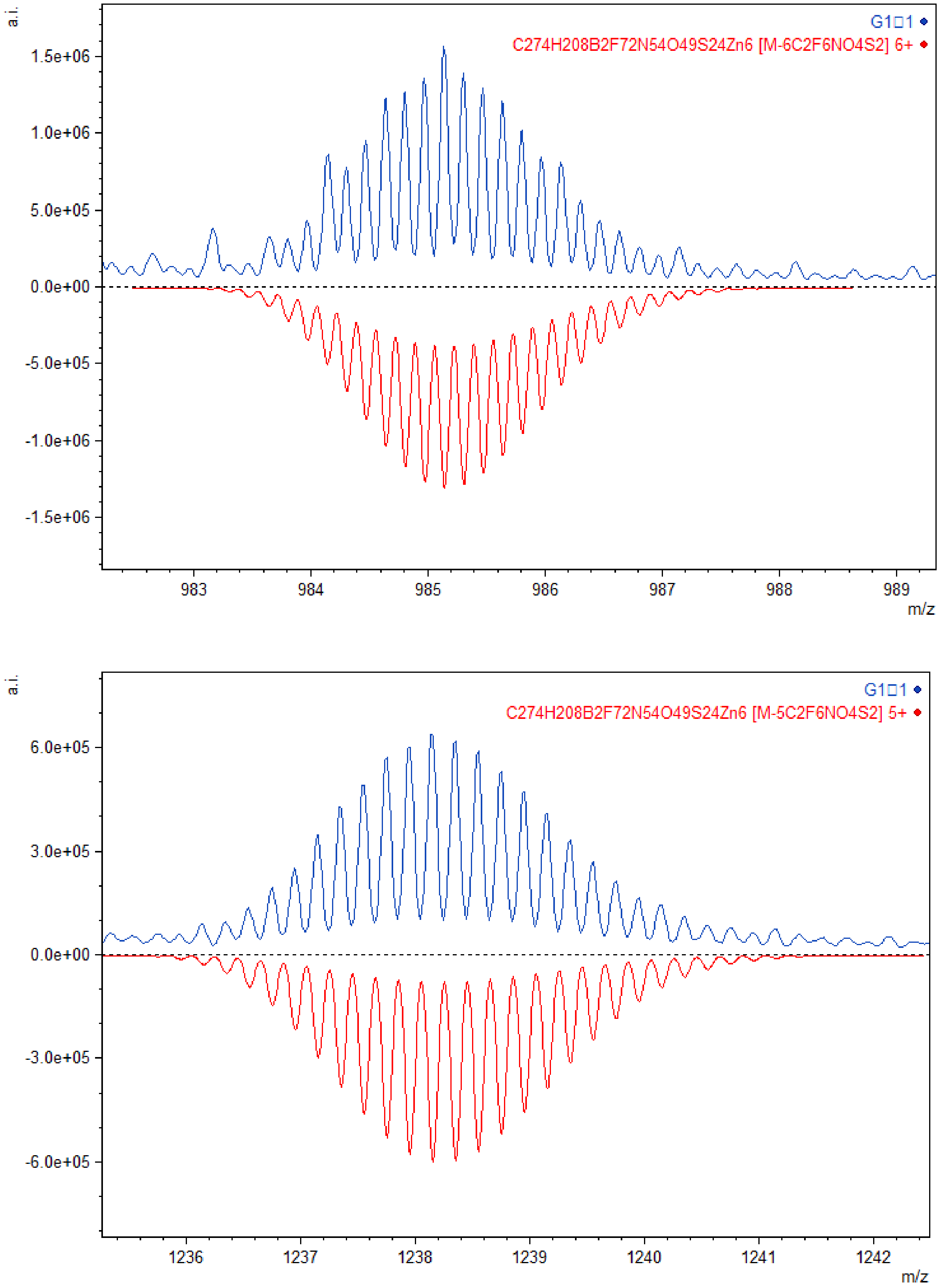

Figure S31. High-resolution ESI-mass spectrometry analysis of **G1⊂1** showing the observed (blue) and theoretical (red) isotope patterns for the +6 and +5 peaks.

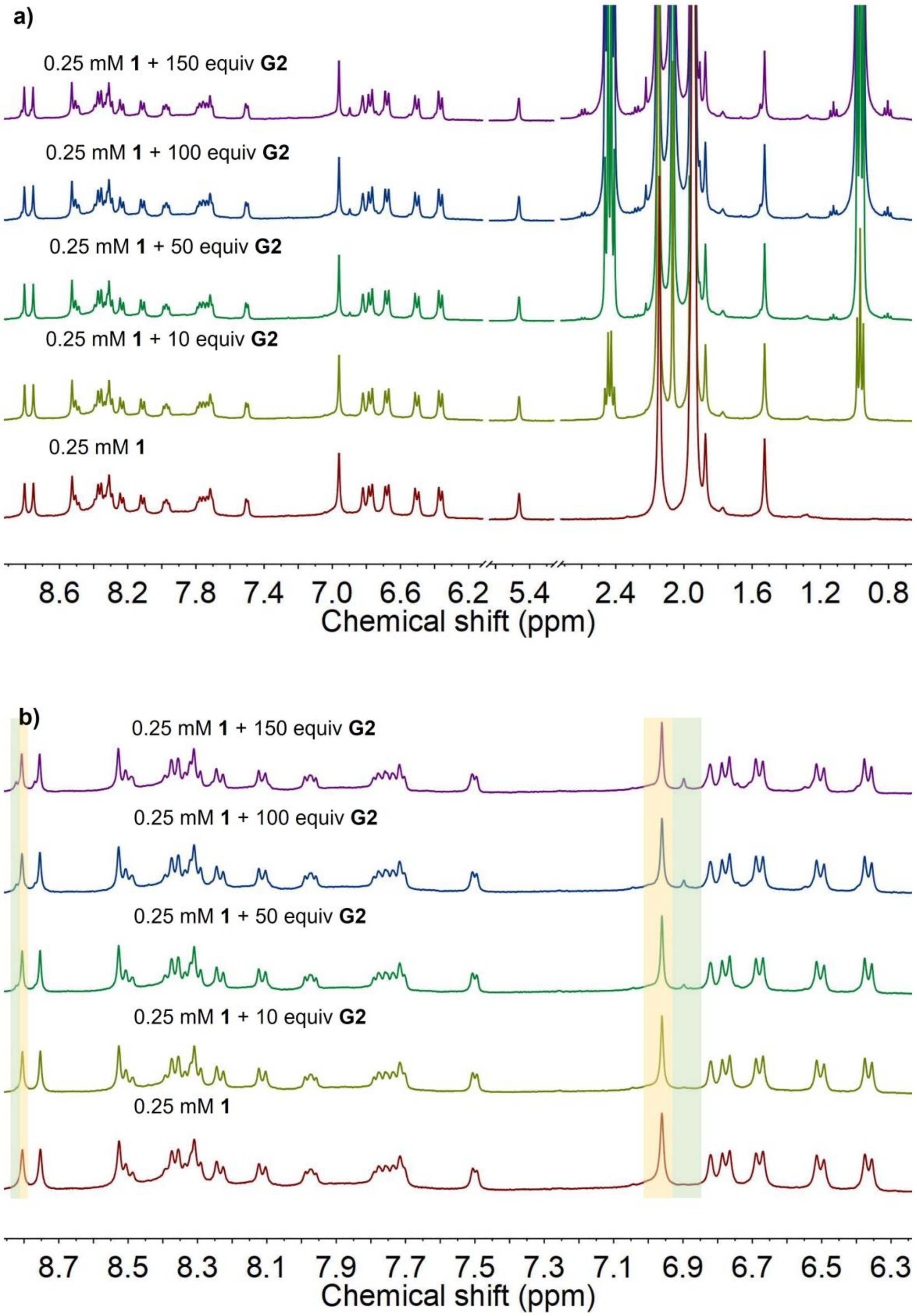

Figure S32. ^{1}H NMR spectra (500 MHz, CD_3CN, 298 K) of a) 0.25 mM **1** with different amounts of **G2** (2-butanone, 10 equiv, 50 equiv, 100 equiv, 150 equiv); b) Expanded view of the aromatic region of a). Selected peaks for **G2⊂1** and free **1** are highlighted by light green and light yellow shading respectively. The binding constants was calculated to be 5.15 ± 1.3 M^{-1}.

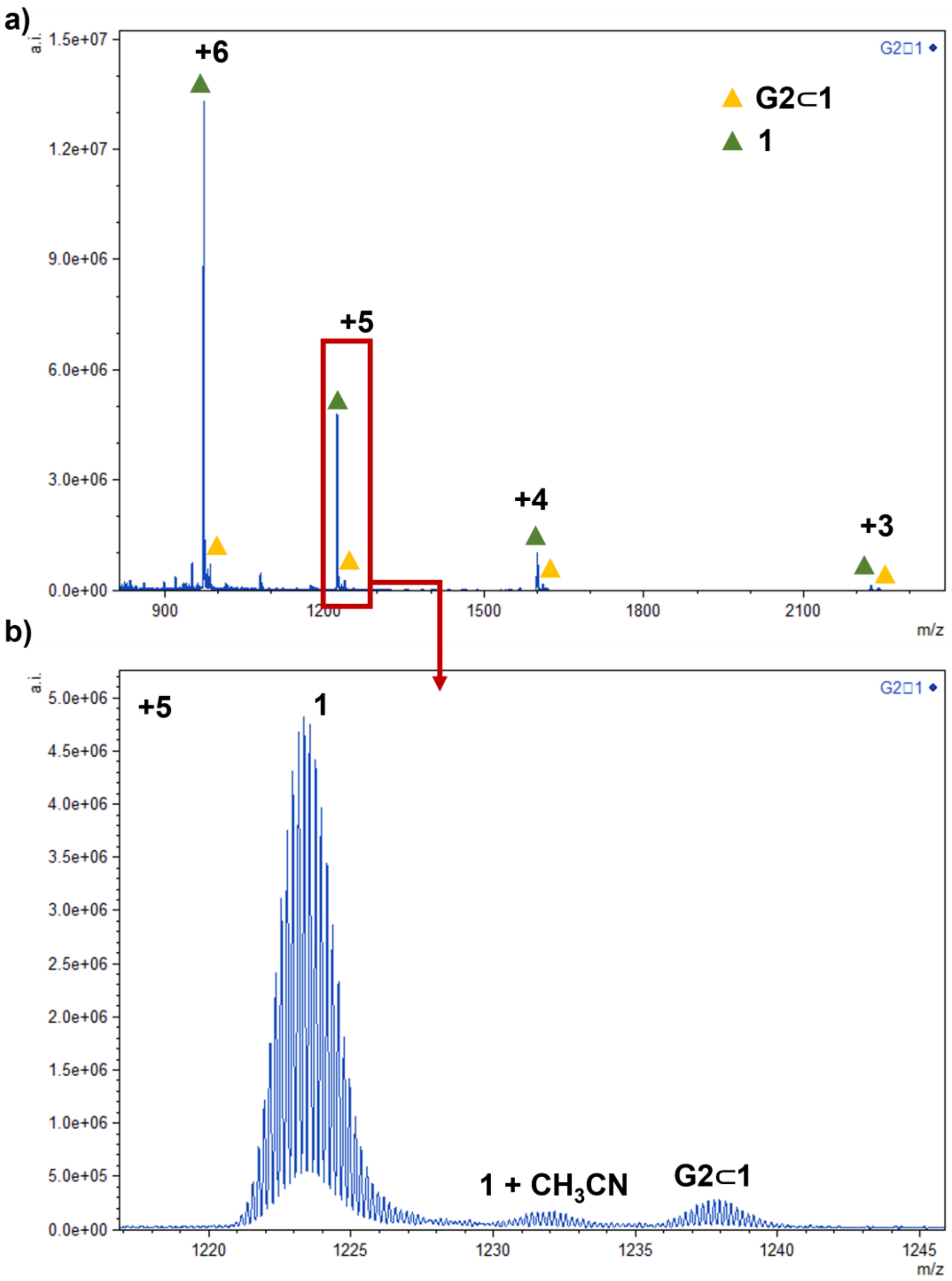

Figure S33. a) High-resolution ESI-mass spectrometry analysis of **1** mixed with 150 equiv **G2**, showing the +6, +5, +4 and +3 peaks ; b) Expanded view showing the +5 peaks.

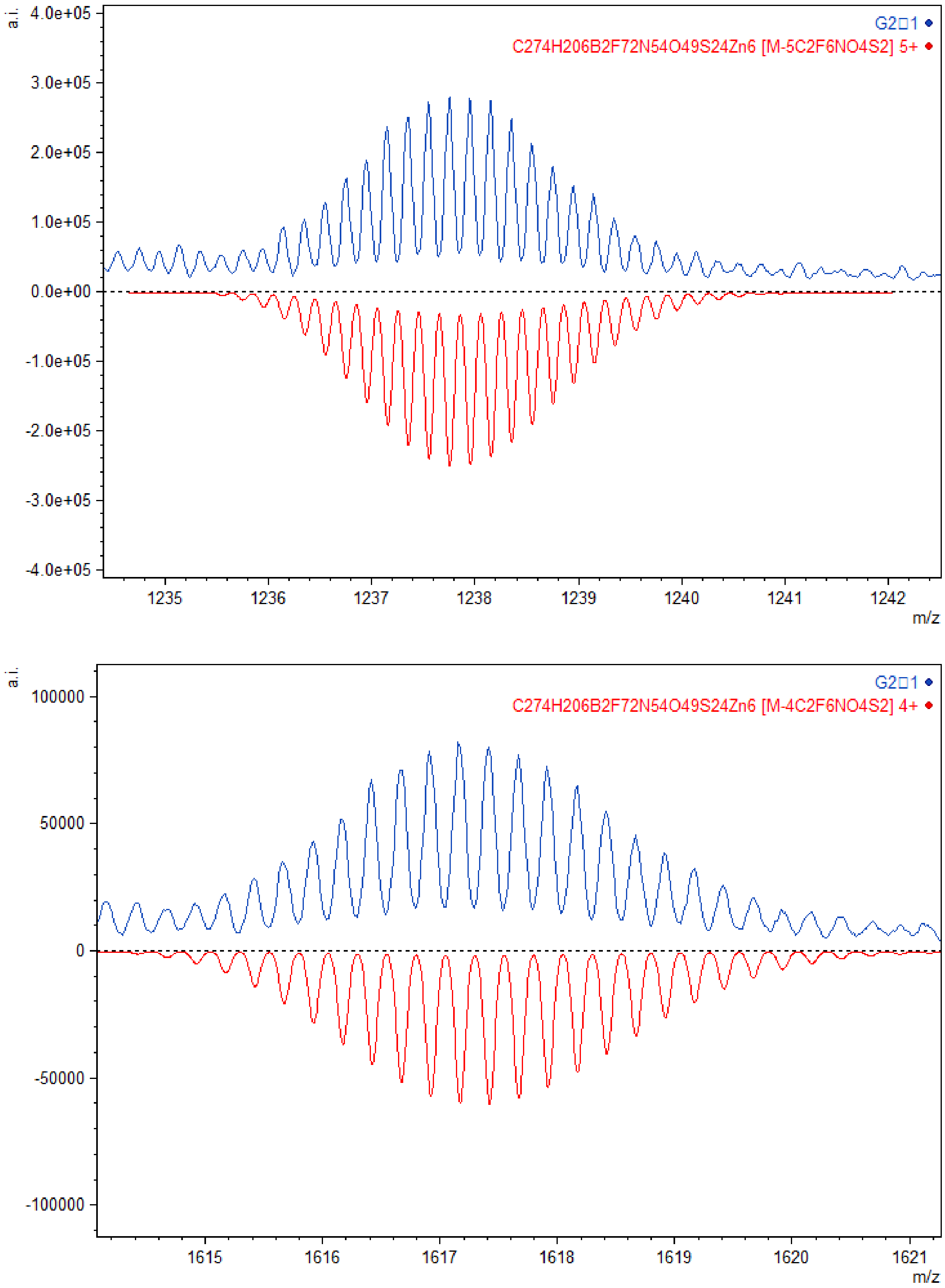

Figure S34. High-resolution ESI-mass spectrometry analysis of **G2⊂1** showing the observed (blue) and theoretical (red) isotope patterns for the +5 and +4 peaks.

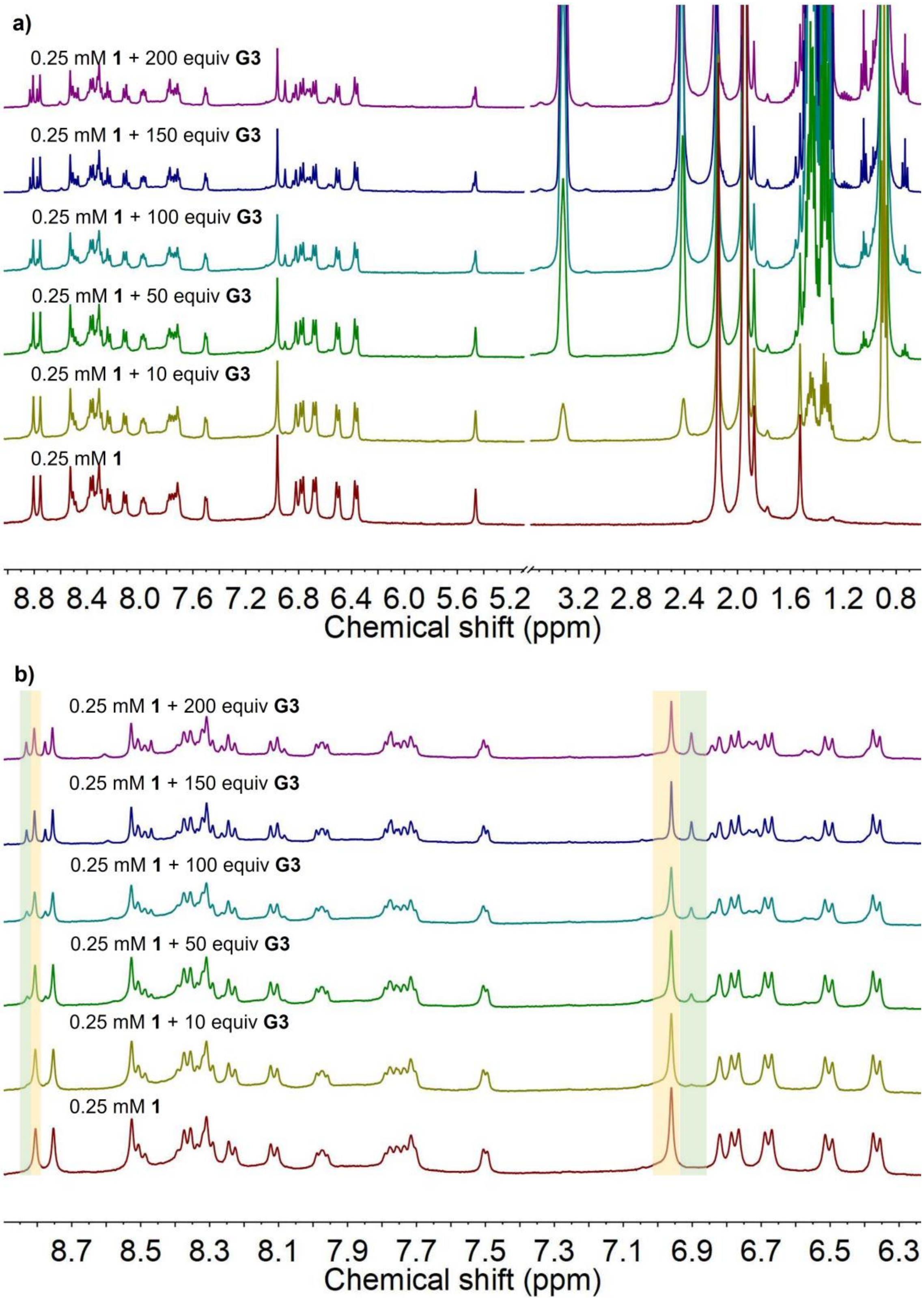

Figure S35. ^{1}H NMR spectra (500 MHz, CD_3CN, 298 K) of a) 0.25 mM **1** with different amounts of **G3** (3-pentanol, 10 equiv, 50 equiv, 100 equiv, 150 equiv and 200 equiv) ; b) Expanded view of the aromatic region of a). Selected peaks for **G3⊂1** and free **1** are highlighted by light green and light yellow shading respectively. The binding constants was calculated to be 26.1 ± 8.1 M^{-1}.

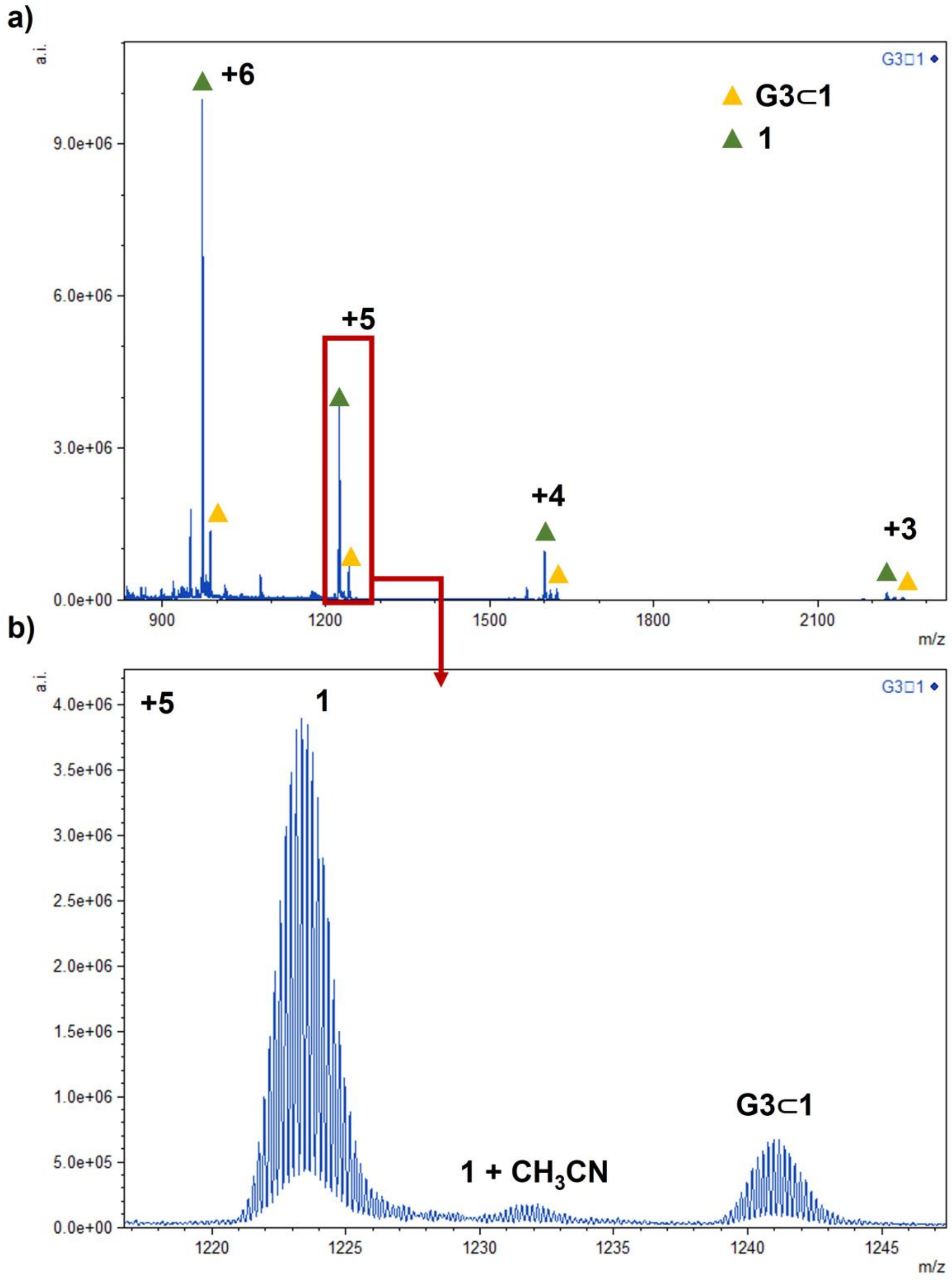

Figure S36. a) High-resolution ESI-mass spectrometry analysis of **1** mixed with 200 equiv **G3**, showing the +6, +5, +4 and +3 peaks ; b) Expanded view showing the +5 peaks.

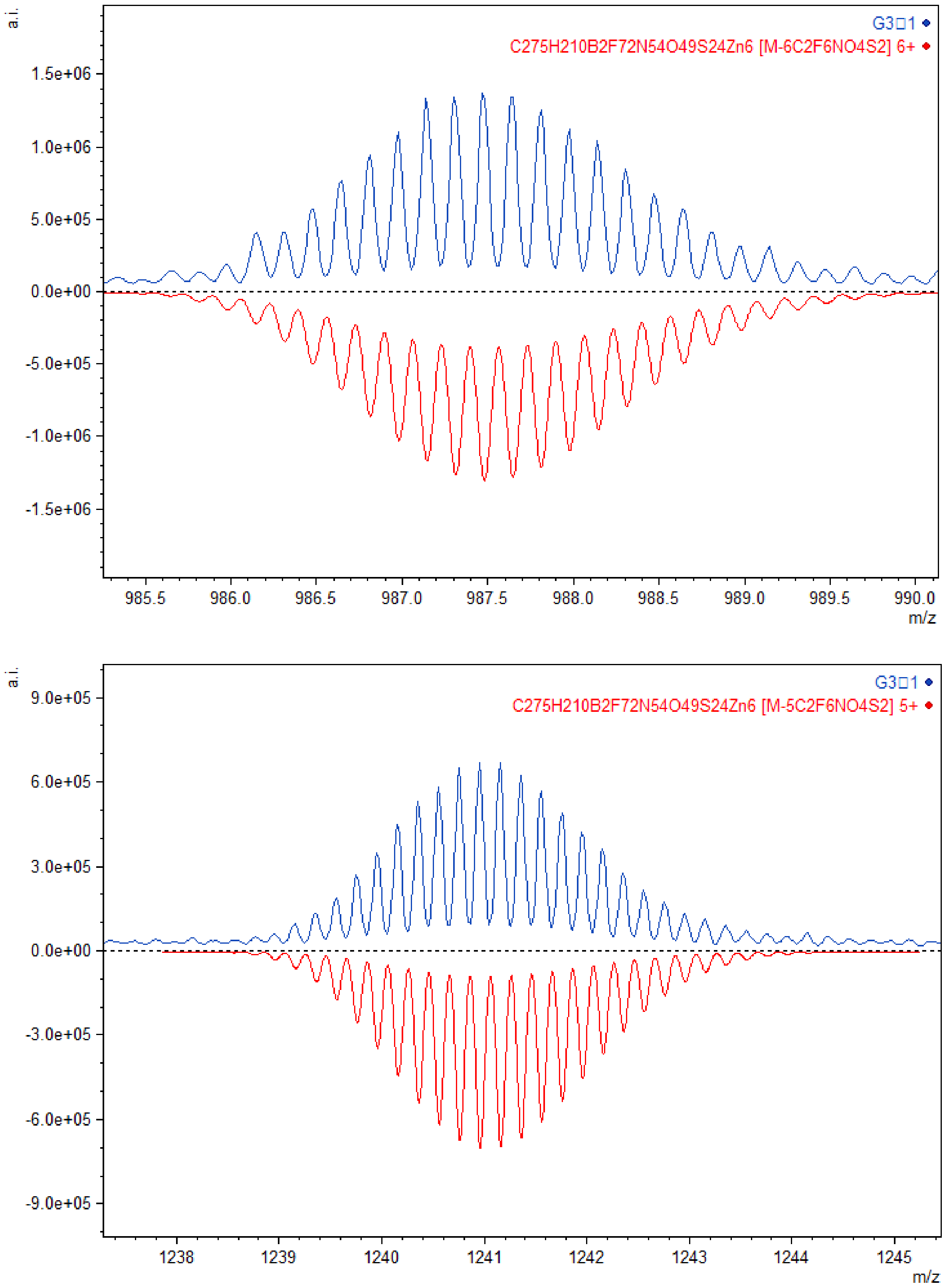

Figure S37. High-resolution ESI-mass spectrometry analysis of **G3⊂1** showing the observed (blue) and theoretical (red) isotope patterns for the +6 and +5 peaks.

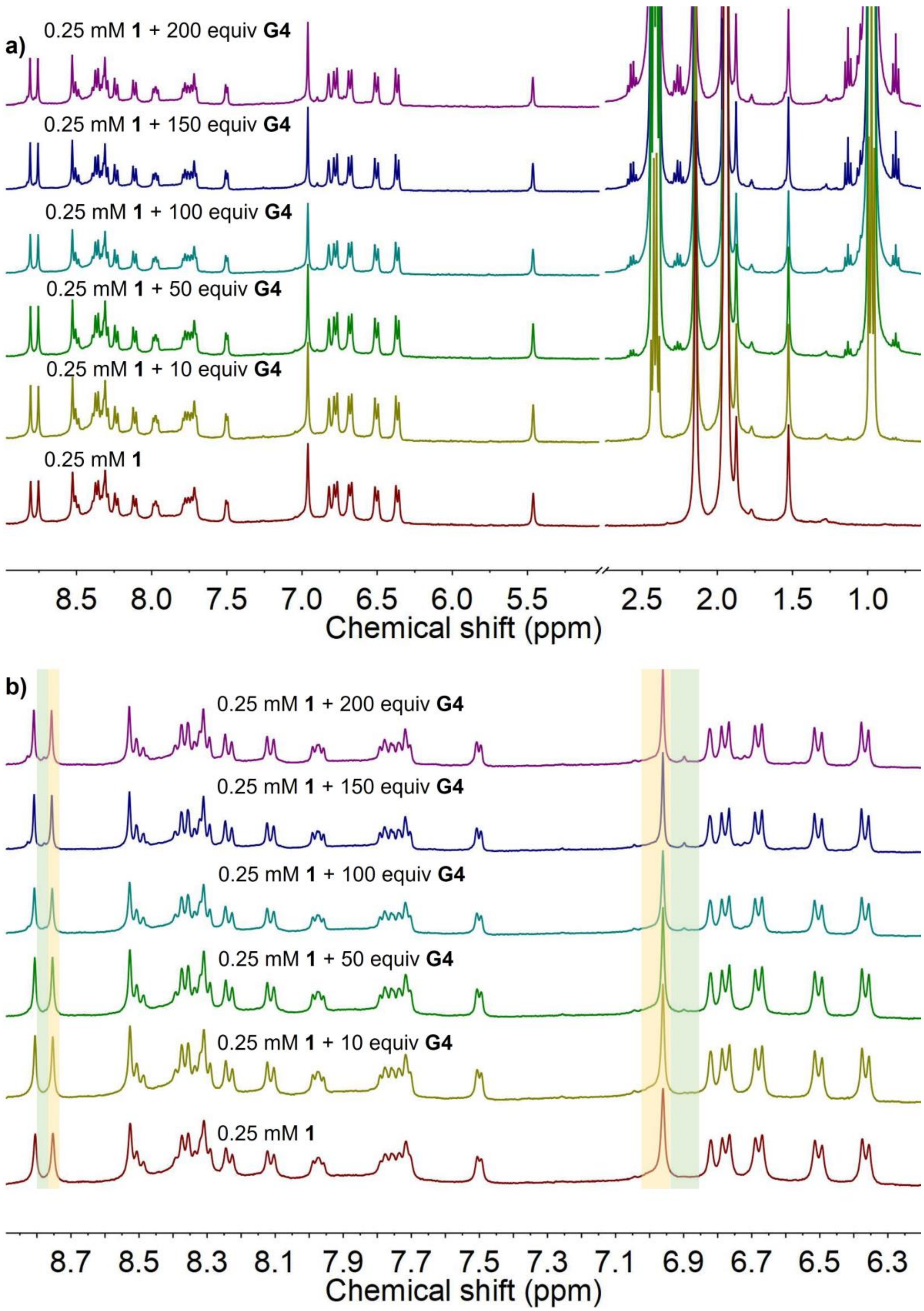

Figure S38. ^{1}H NMR spectra (500 MHz, CD_3CN, 298 K) of a) 0.25 mM **1** with different amounts of **G4** (3-pentanone, 10 equiv, 50 equiv, 100 equiv, 150 equiv and 200 equiv) ; b) Expanded view of the aromatic region of a). Selected peaks for **G4⊂1** and free **1** are highlighted by light green and light yellow shading respectively. The binding constants was calculated to be 4.05 ± 1.1 M^{-1}.

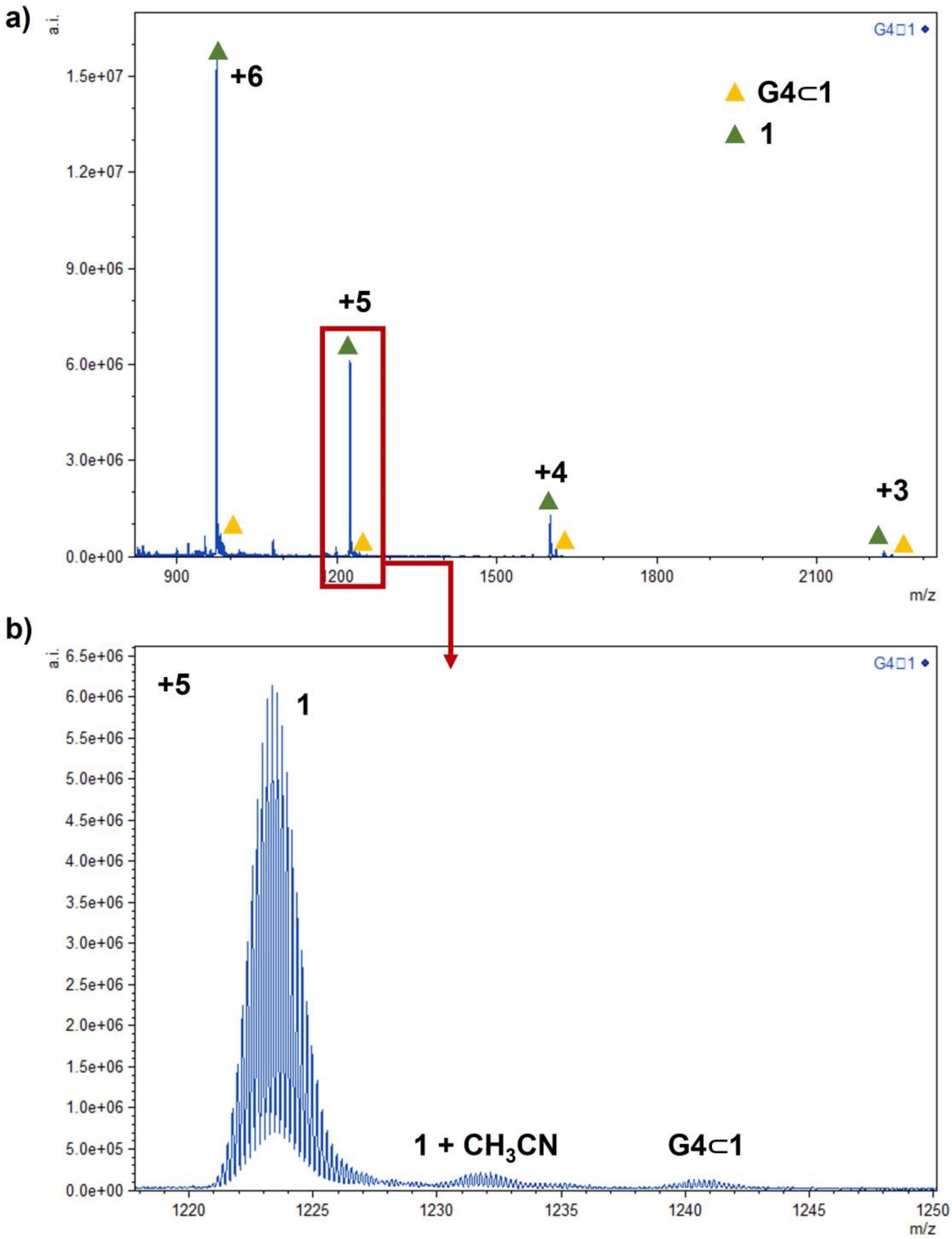

Figure S39. a) High-resolution ESI-mass spectrometry analysis of **1** mixed with 200 equiv **G4**, showing the +6, +5, +4 and +3 peaks ; b) Expanded view of the +5 peaks.

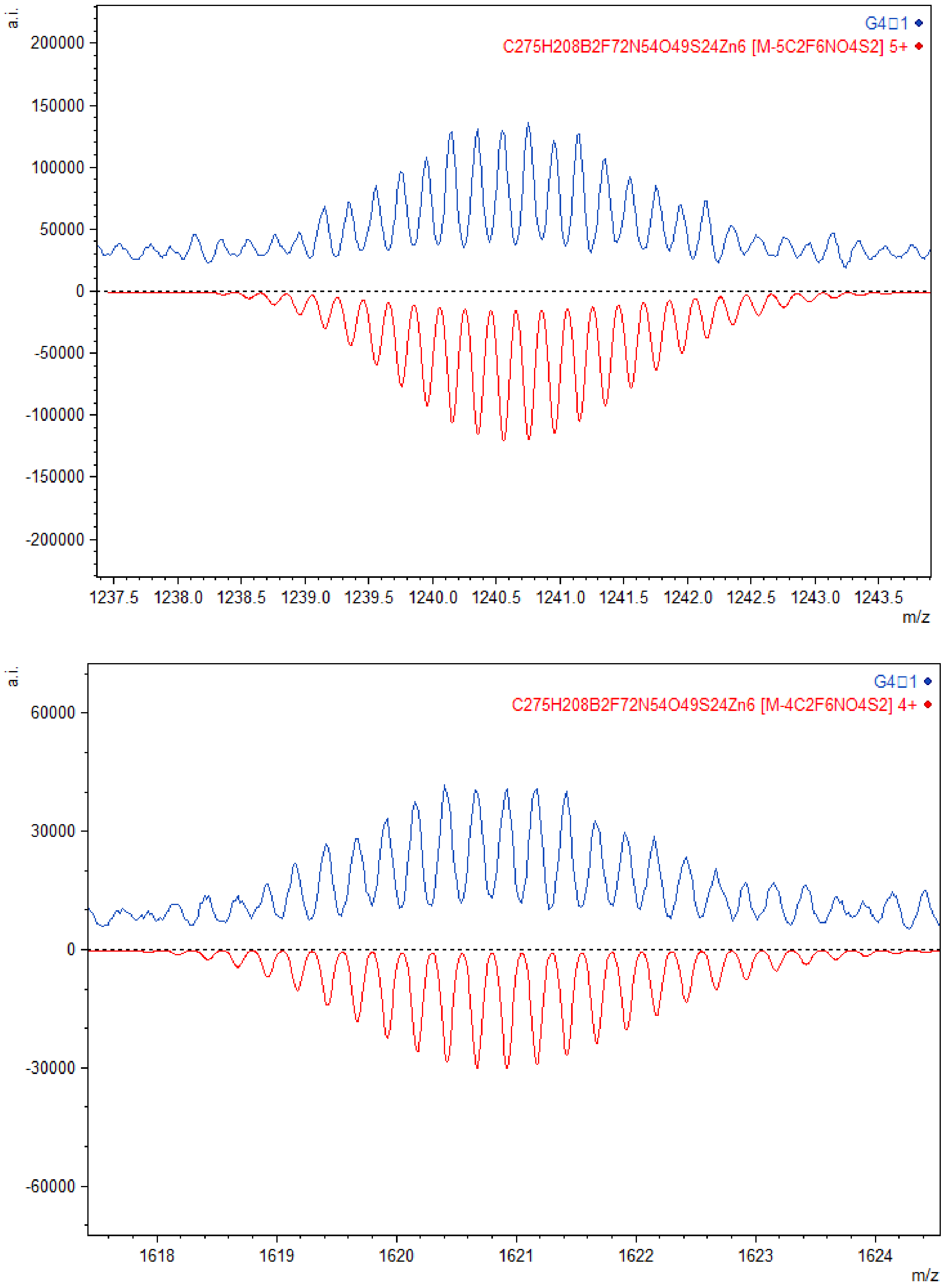

Figure S40. High-resolution ESI-mass spectrometry analysis of **G4⊂1** showing the observed (blue) and theoretical (red) isotope patterns for the +5 and +4 peaks.

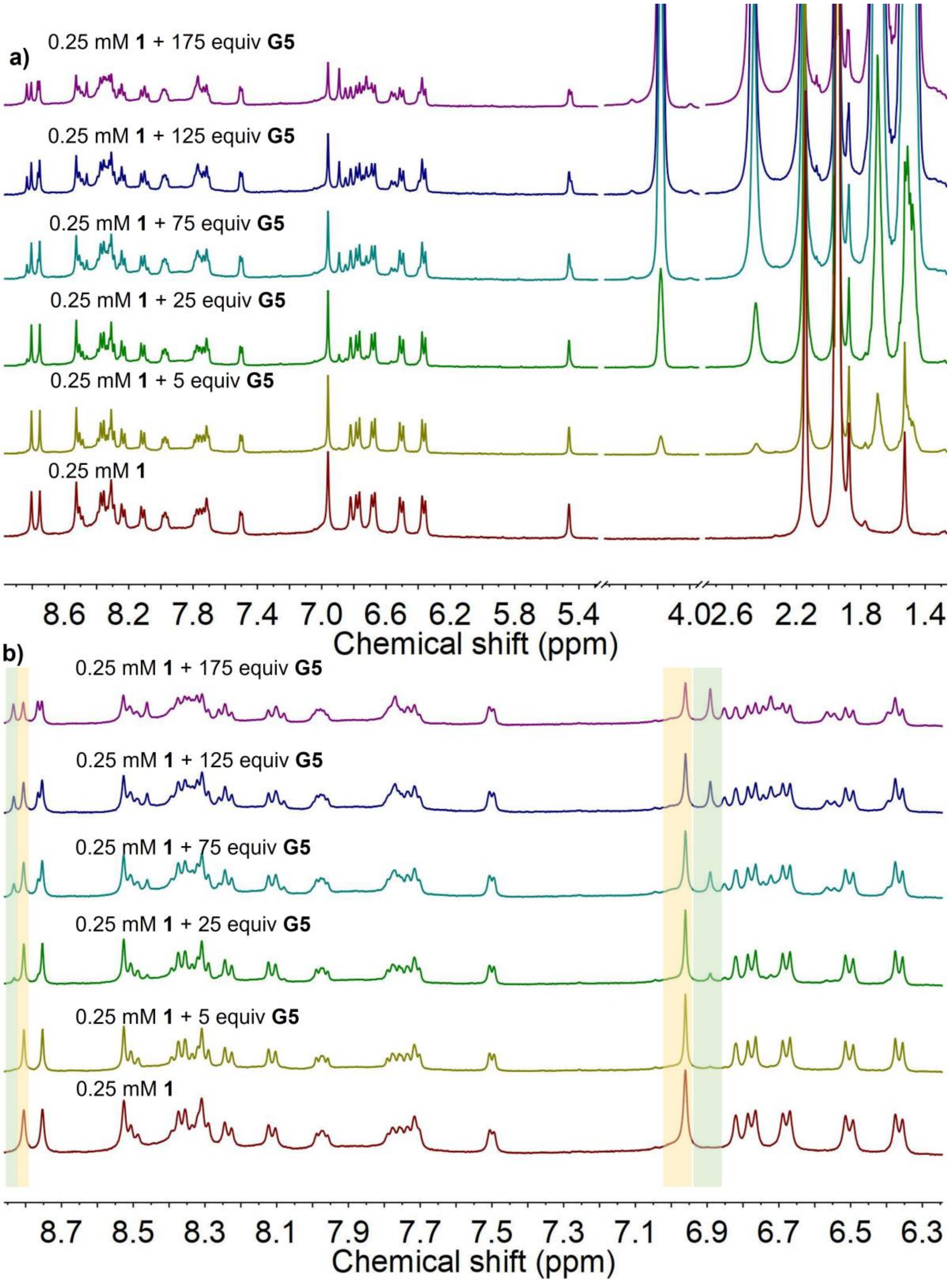

Figure S41. ^{1}H NMR spectra (500 MHz, CD_3CN, 298 K) of a) 0.25 mM **1** with different amounts of **G5** (cyclopentanol, 5 equiv, 25 equiv, 75 equiv, 125 equiv and 175 equiv) ; b) Expanded view of the aromatic region of a). Selected peaks for **G5⊂1** and free **1** are highlighted by light green and light yellow shading respectively. The binding constants was calculated to be 37.8 ± 7.4 M^{-1}.

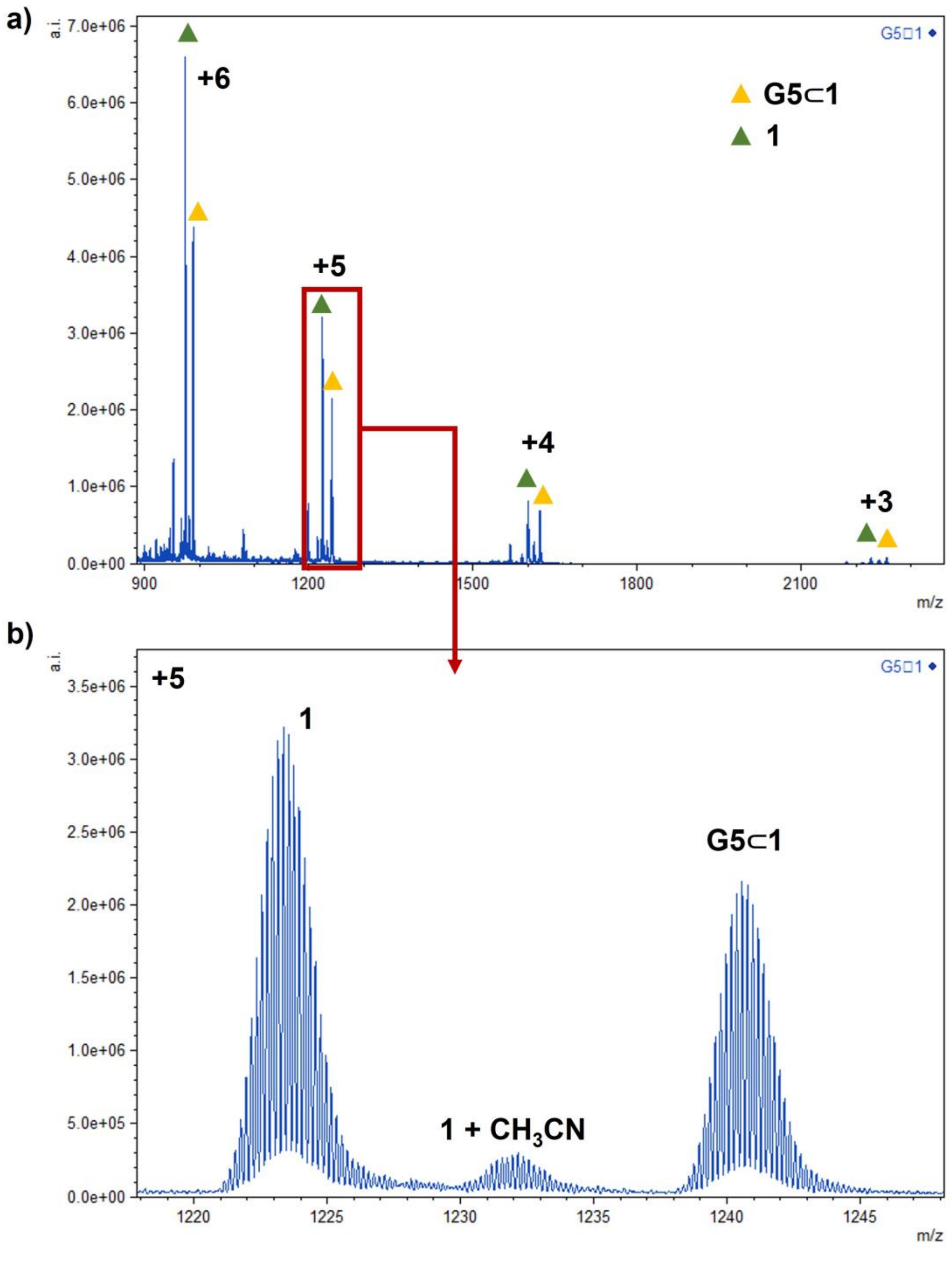

Figure S42. a) High-resolution ESI-mass spectrometry analysis of **1** mixed with 175 equiv **G5**, showing the +6, +5, +4 and +3 peaks ; b) Expanded view showing the +5 peaks.

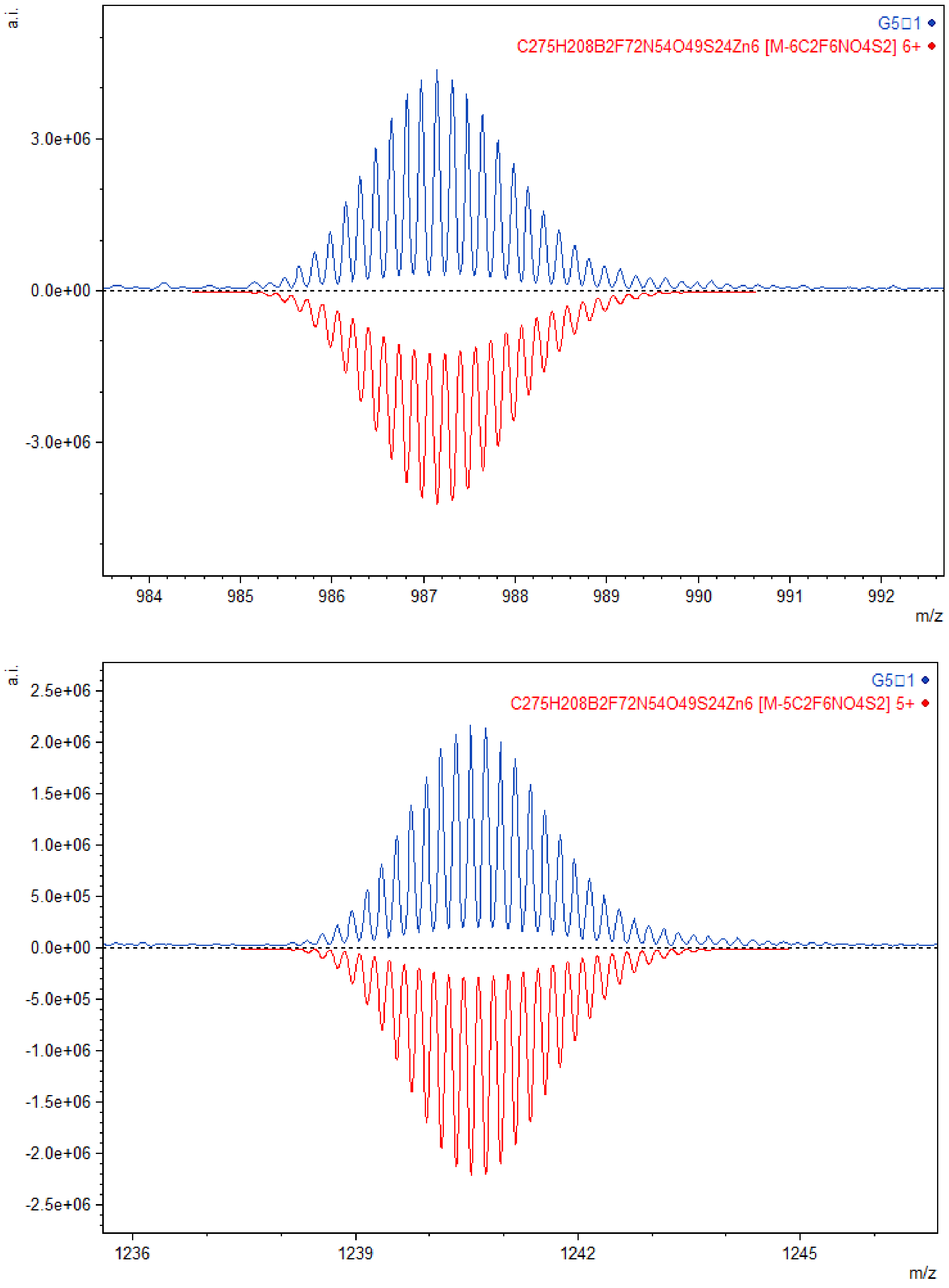

Figure S43. High-resolution ESI-mass spectrometry analysis of **G5⊂1** showing the observed (blue) and theoretical (red) isotope patterns for the +6 and +5 peaks.

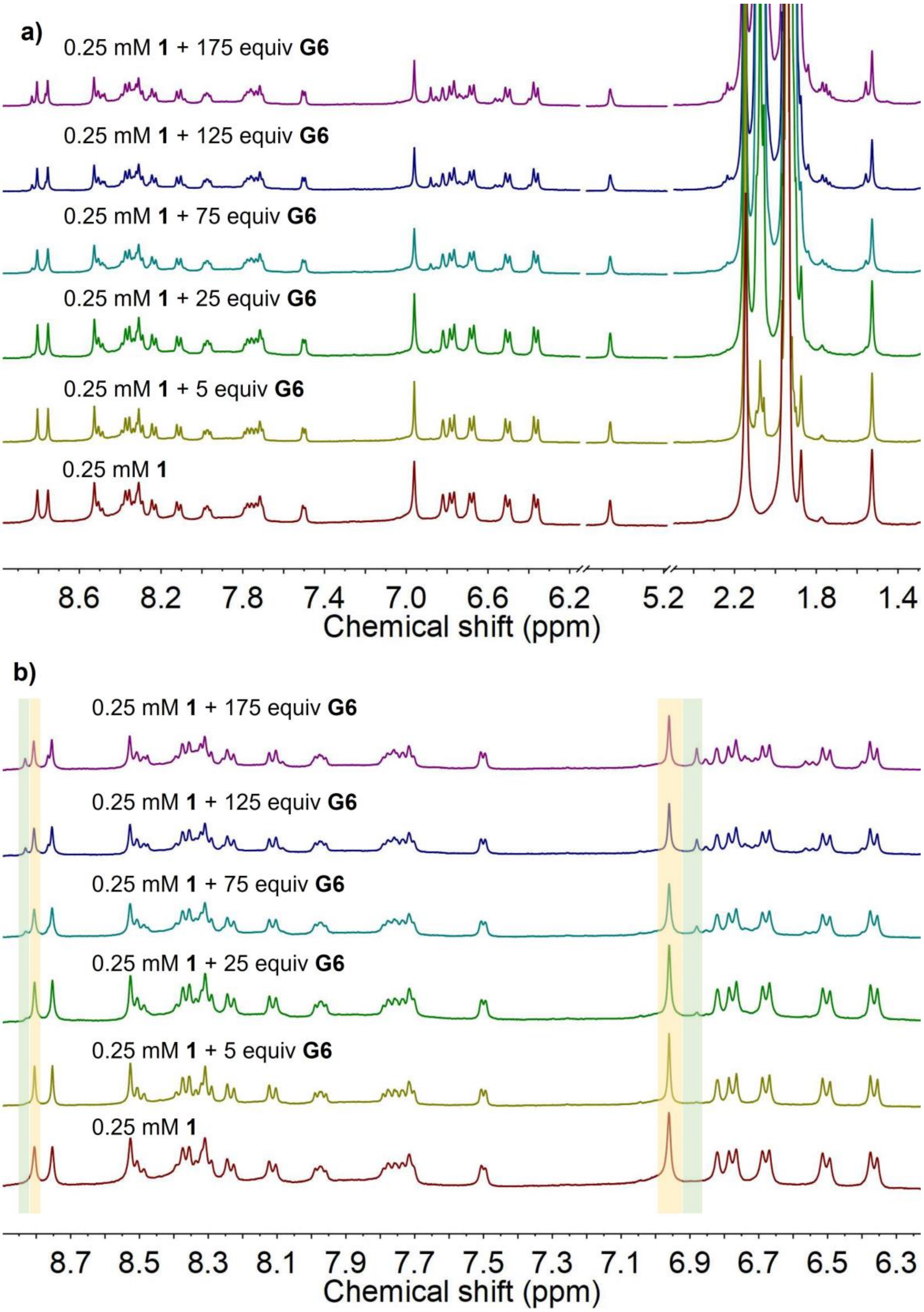

Figure S44. ^{1}H NMR spectra (500 MHz, CD_3CN, 298 K) of a) 0.25 mM **1** with different amounts of **G6** (cyclopentanone, 5 equiv, 25 equiv, 75 equiv, 125 equiv and 175 equiv) ; b) Expanded view of the aromatic region of a). Selected peaks for **G6⊂1** and free **1** are highlighted by light green and light yellow shading respectively. The binding constants was calculated to be 10.1 ± 0.4 M^{-1}.

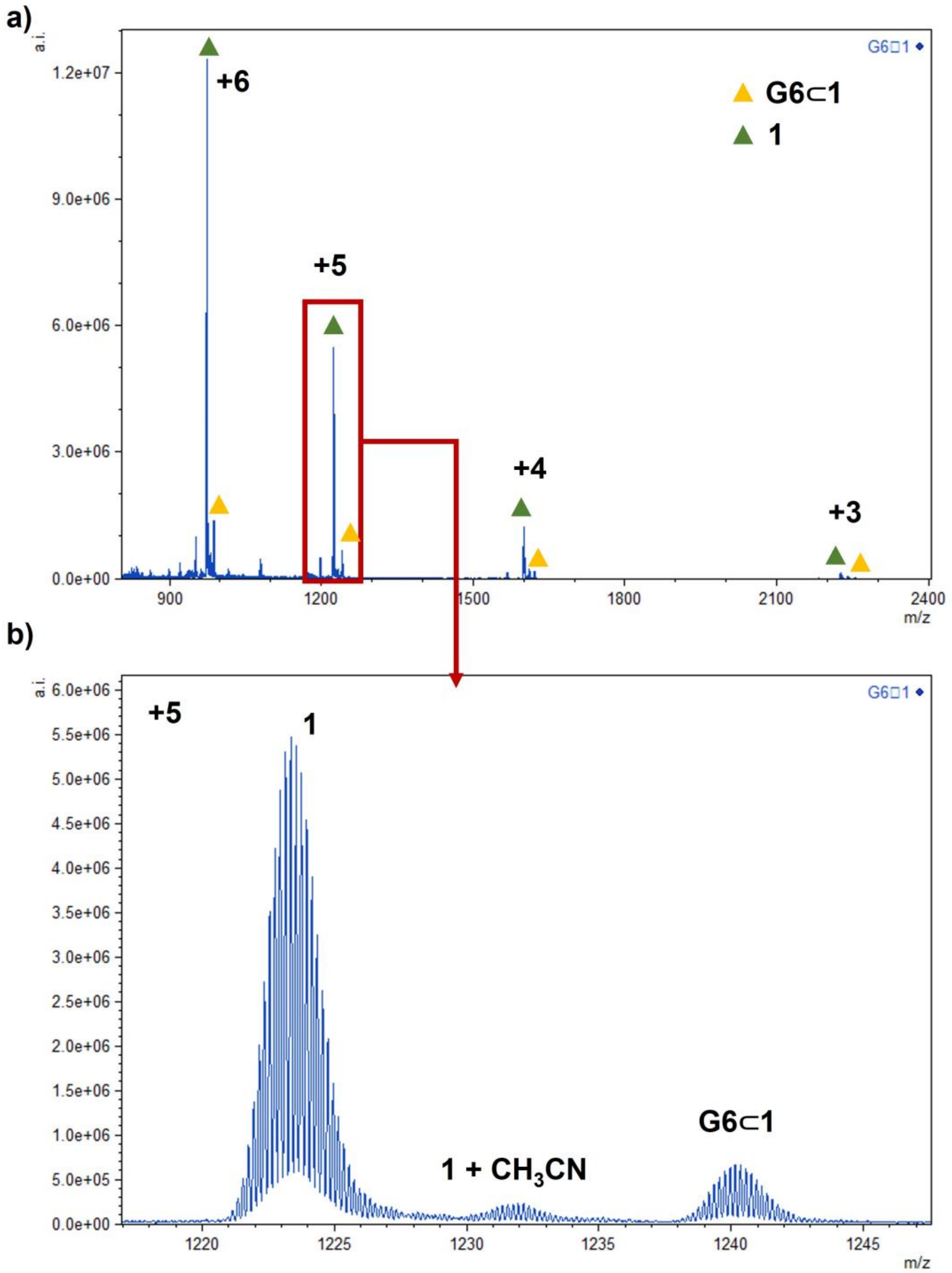

Figure S45. a) High-resolution ESI-mass spectrometry analysis of **1** mixed with 175 equiv **G6**, showing the +6, +5, +4 and +3 peaks ; b) Expand view showing the +5 peaks.

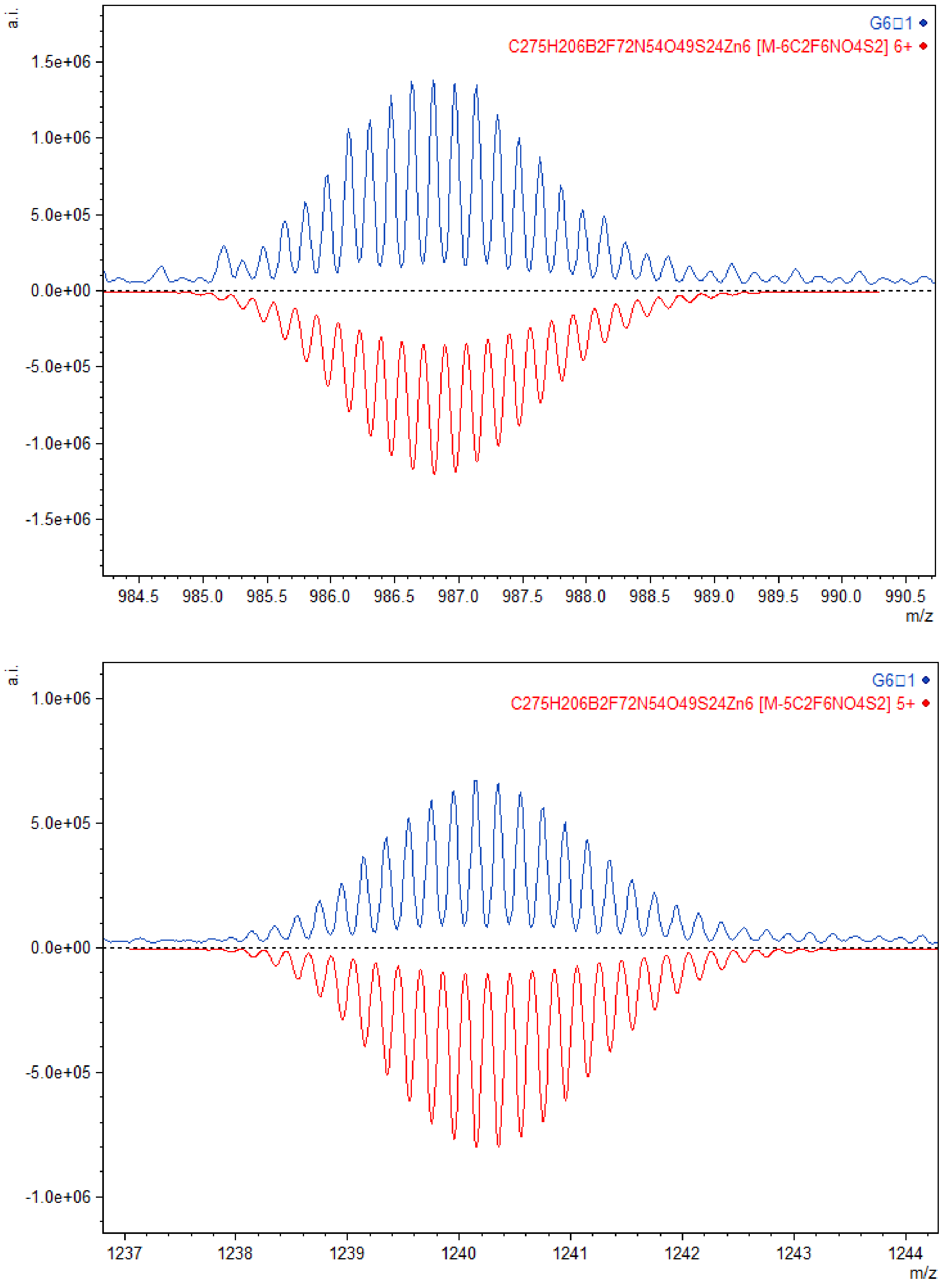

Figure S46. High-resolution ESI-mass spectrometry analysis of **G6⊂1** showing the observed (blue) and theoretical (red) isotope patterns for the +6 and +5 peaks.

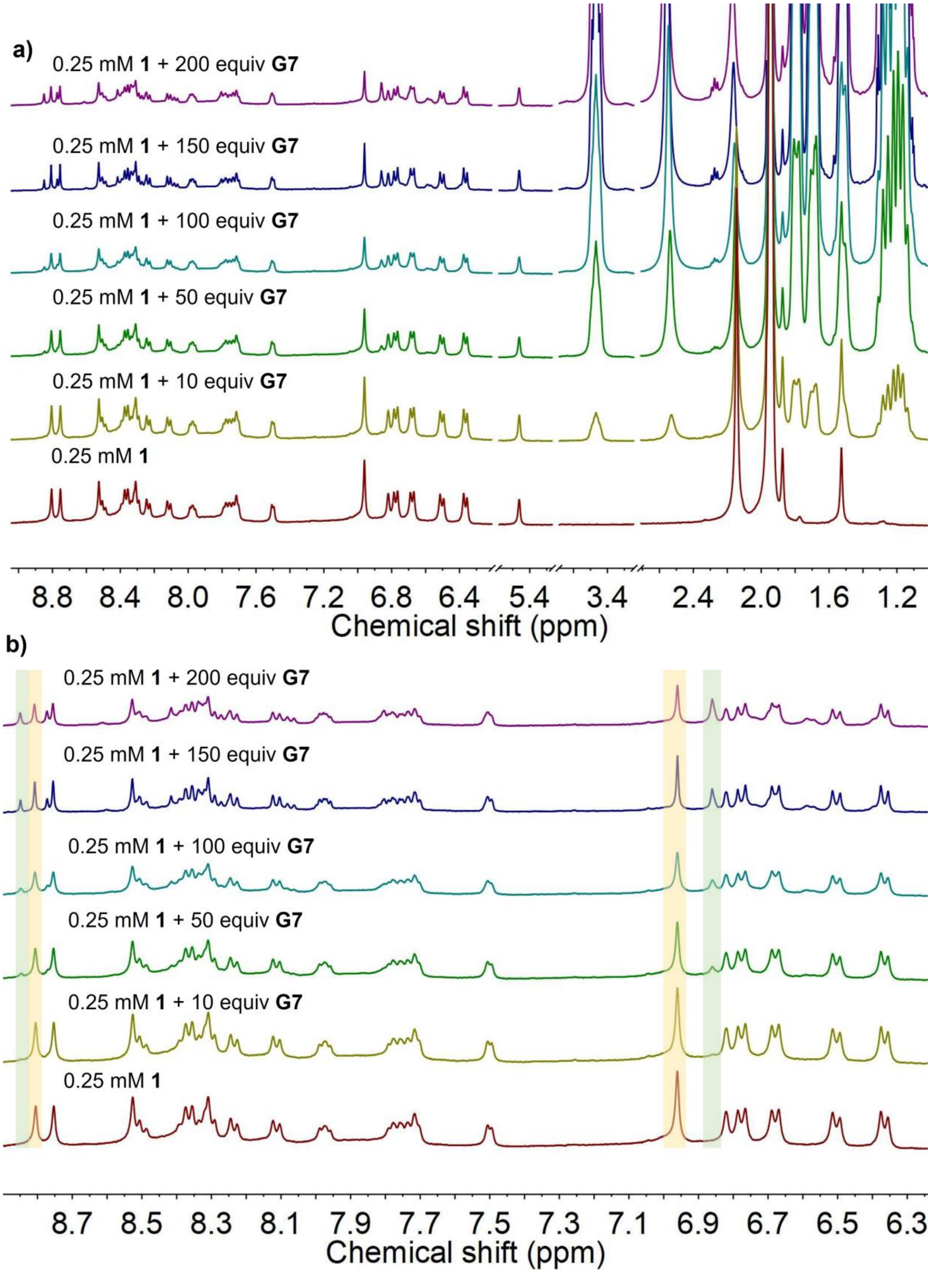

Figure S47. ^{1}H NMR spectra (500 MHz, CD_3CN, 298 K) of a) 0.25 mM **1** with different amounts of **G7** (cyclohexanol, 10 equiv, 50 equiv, 100 equiv, 150 equiv and 200 equiv) ; b) Expand view of the aromatic region of a). Selected peaks for **G7⊂1** and free **1** are highlighted by light green and light yellow shading respectively. The binding constants was calculated to be 26.9 ± 4.8 M^{-1}.

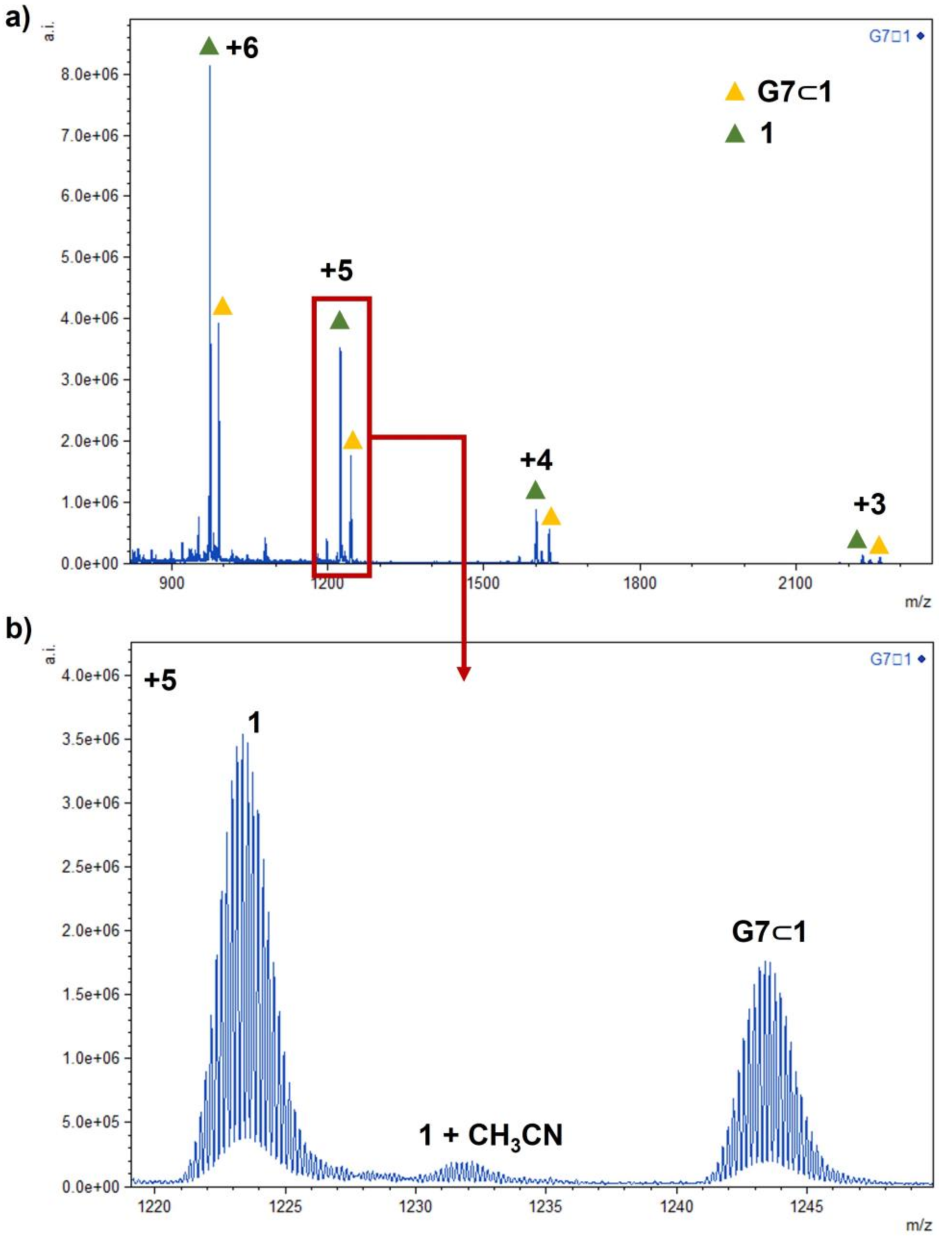

Figure S48. a) High-resolution ESI-mass spectrometry analysis of **1** mixed with 200 equiv **G7**, showing the +6, +5, +4 and +3 peaks ; b) Expanded view showing the +5 peaks.

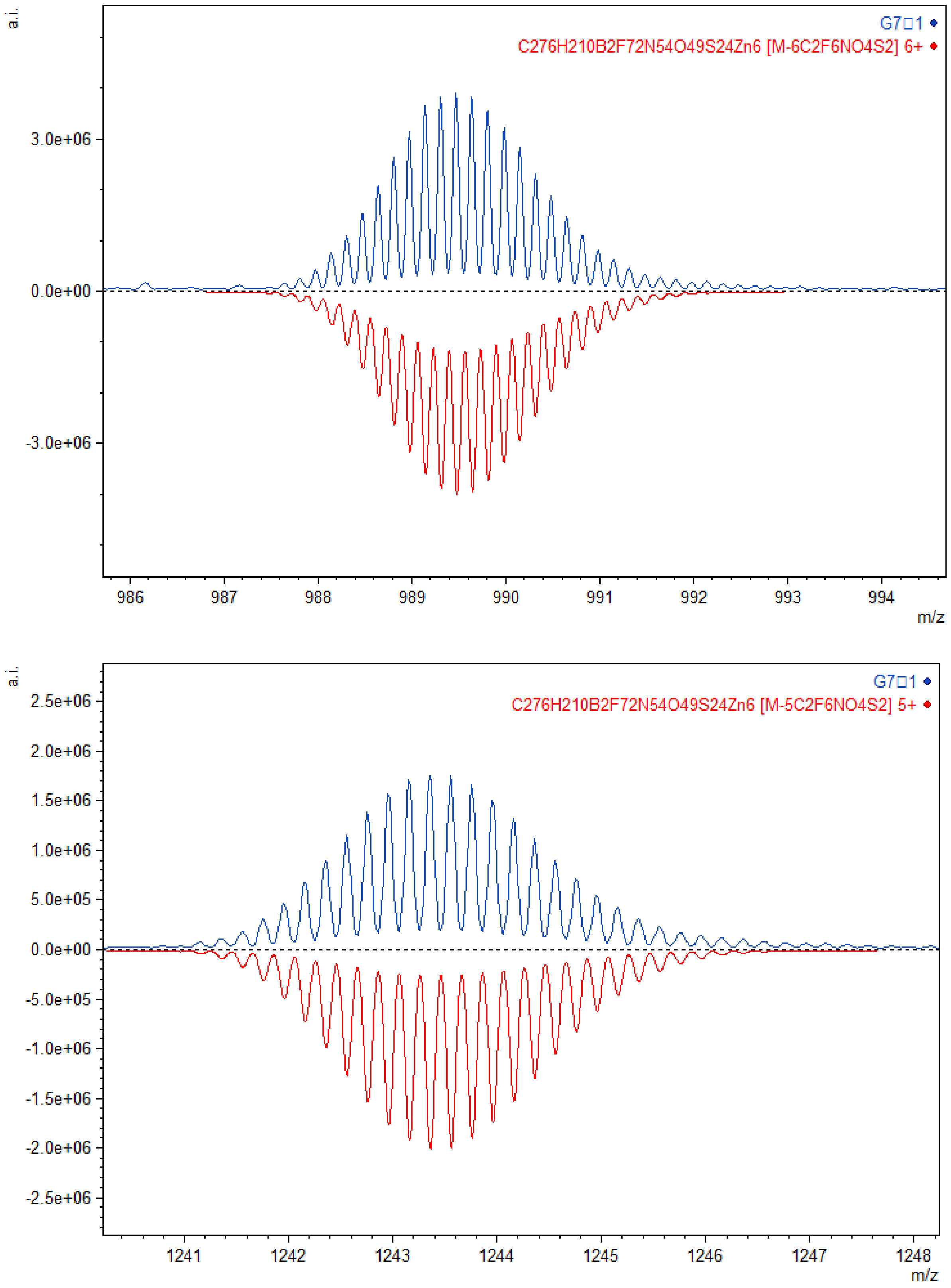

Figure S49. High-resolution ESI-mass spectrometry analysis of **G7⊂1** showing the observed (blue) and theoretical (red) isotope patterns for the +6 and +5 peaks.

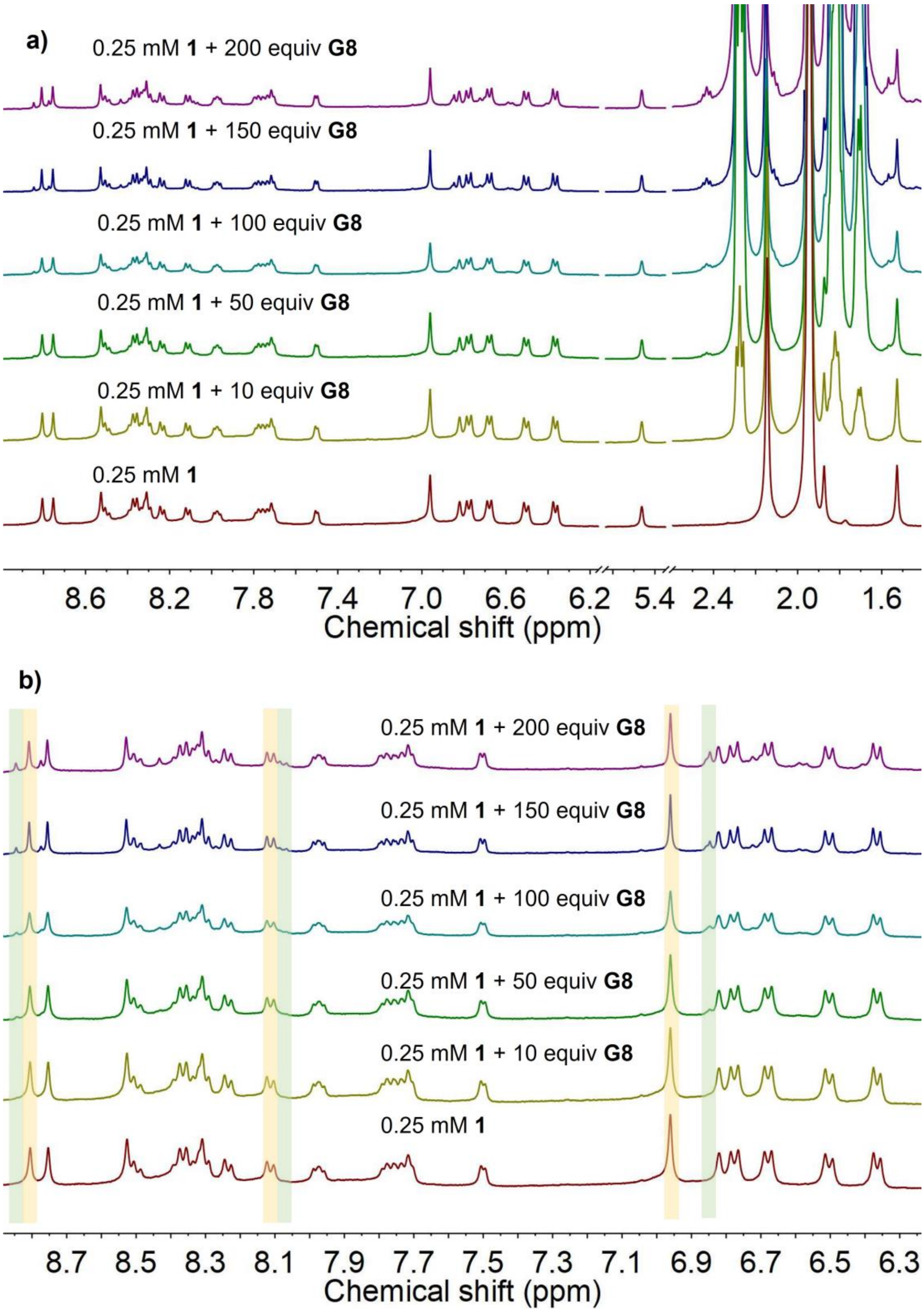

Figure S50. ^{1}H NMR spectra (500 MHz, CD_3CN, 298 K) of a) 0.25 mM **1** with different amounts of **G8** (cyclohexanone, 10 equiv, 50 equiv, 100 equiv, 150 equiv and 200 equiv) ; b) Expanded view of the aromatic region of a). Selected peaks for **G8⊂1** and free **1** are highlighted by light green and light yellow shading respectively. The binding constants was calculated to be 10.6 ± 1.5 M^{-1}.

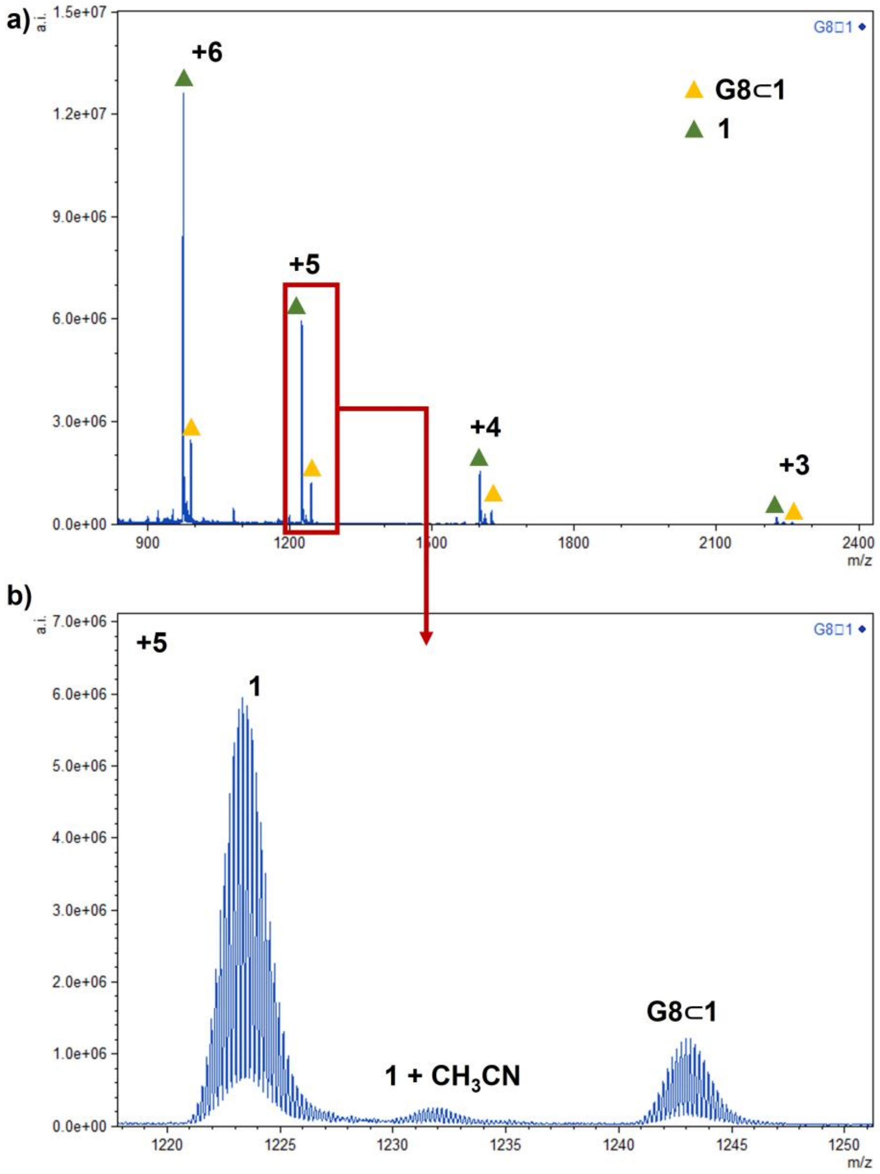

Figure S51. a) High-resolution ESI-mass spectrometry analysis of **1** mixed with 200 equiv **G8**, showing the +6, +5, +4 and +3 peaks ; b) Expanded view of the +5 peaks.

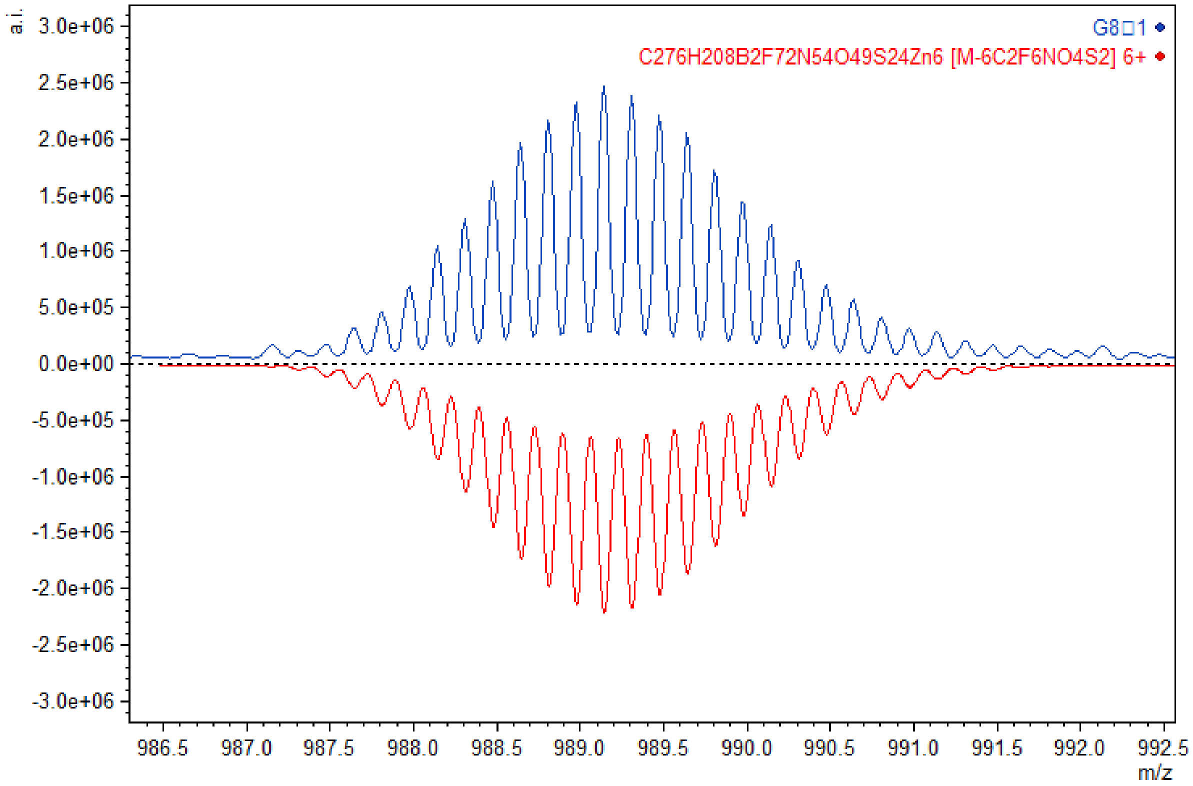

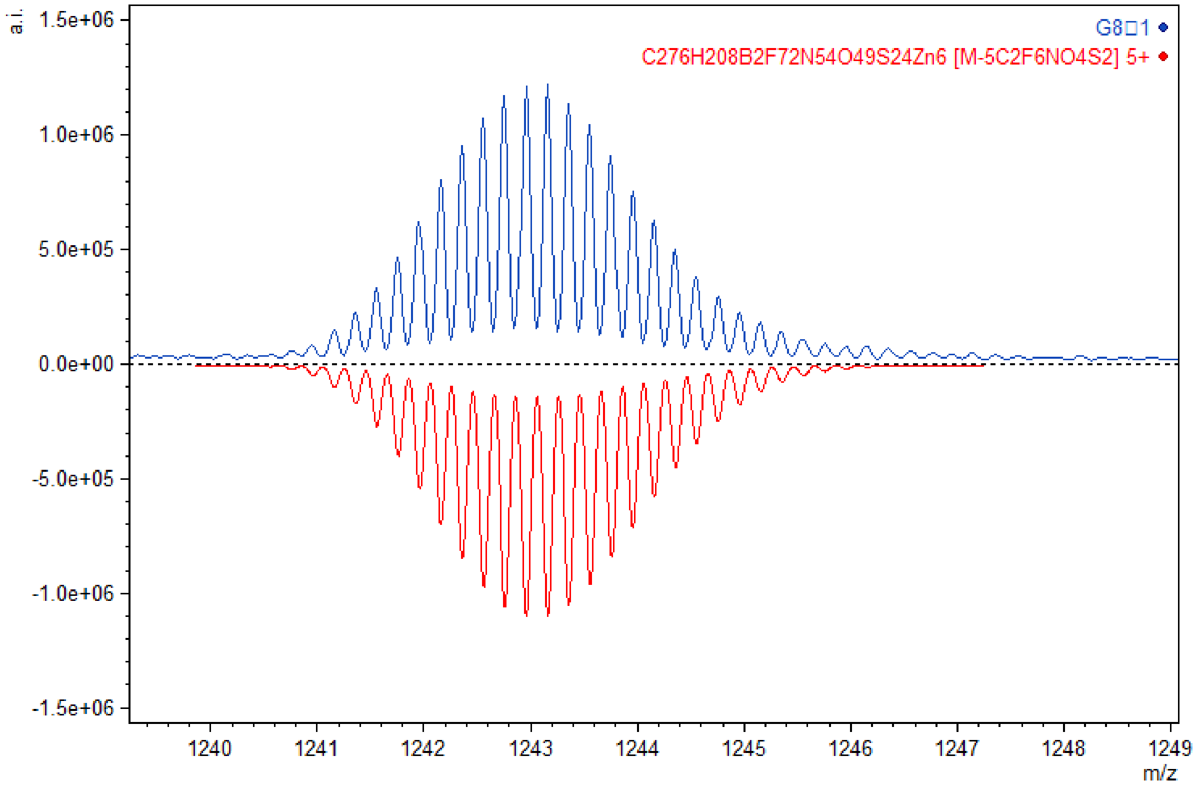

Figure S52. High-resolution ESI-mass spectrometry analysis of **G8⊂1** showing the observed (blue) and theoretical (red) isotope patterns for the +6 and +5 peaks.

3.3 Host-guest interactions of 1·2F⁻ with different guest molecules (G1-G8)

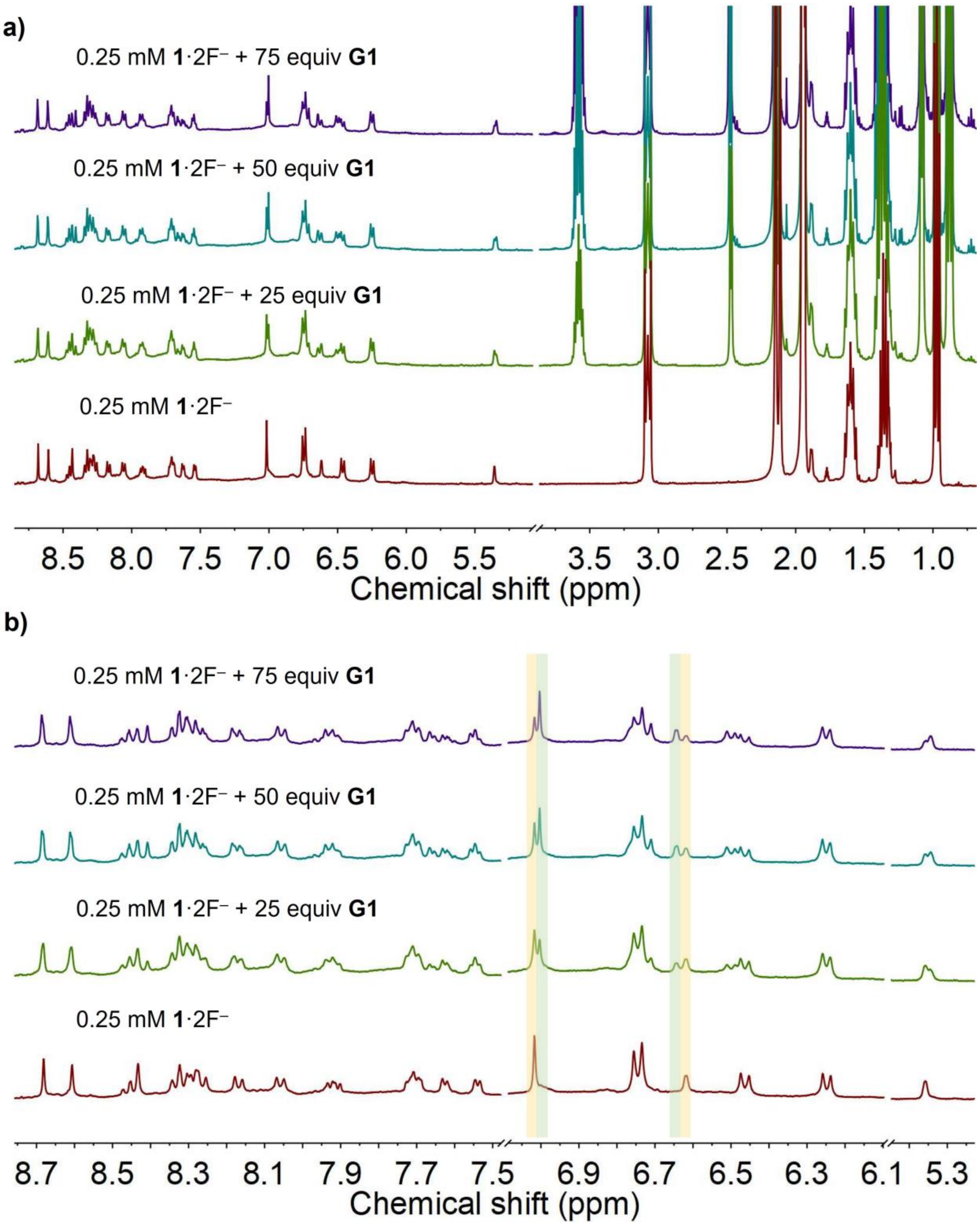

Figure S53: ^{1}H NMR spectra (500 MHz, CD_3CN, 298 K) of a) 0.25 mM **1**·2F⁻ with different amounts of **G1** (2-butanol, 25 equiv, 50 equiv and 75 equiv) ; b) Expanded view of the aromatic region of a). Selected peaks for **G1⊂1**·2F⁻and free **1**·2F⁻are highlighted by light green and light yellow shading respectively. The binding constant was calculated to be 254 ± 56 M^{-1}.

.

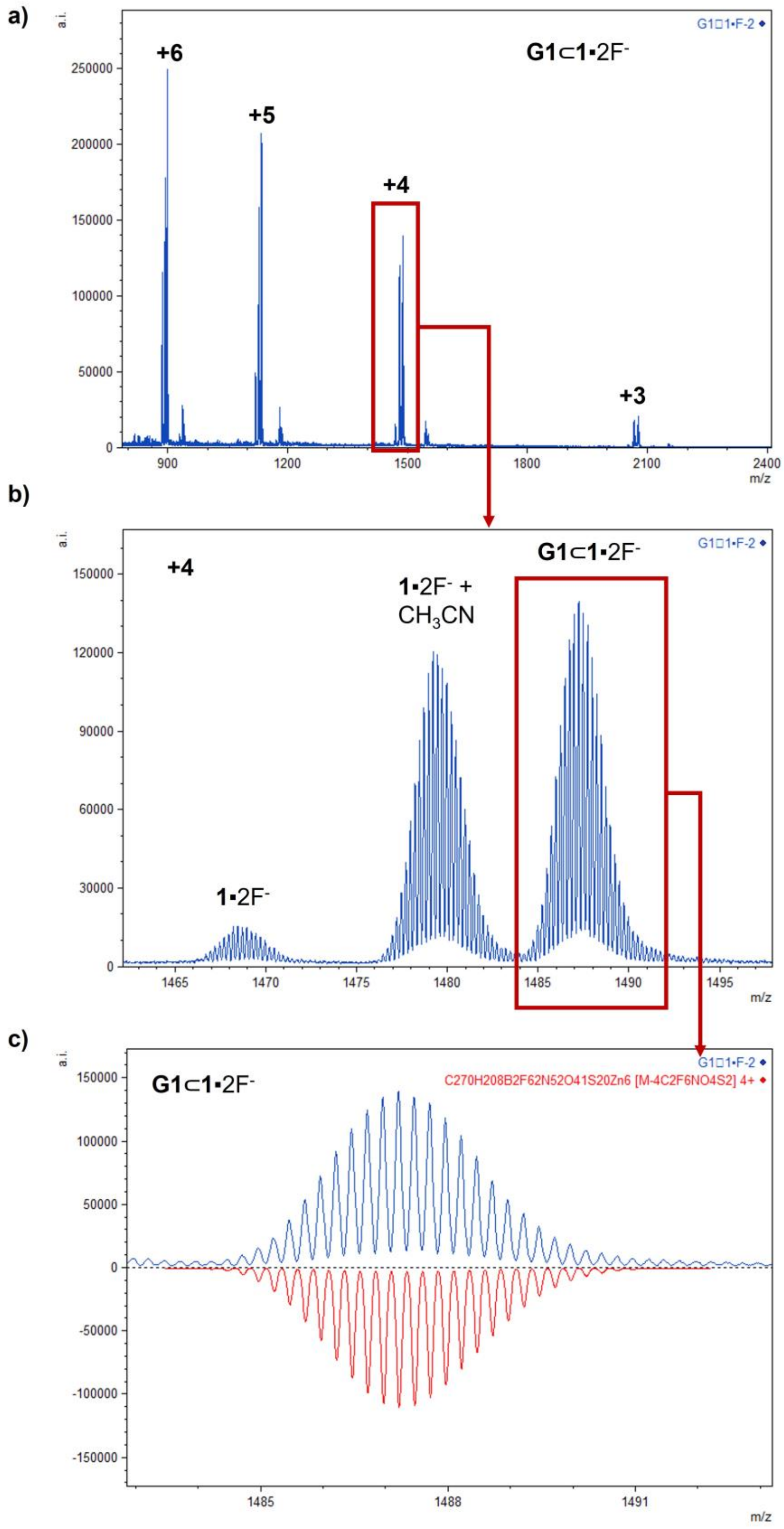

Figure S54. a) High-resolution ESI-mass spectrometry analysis of **1**·F^{-}_{2} mixed with 75 equiv **G1**, showing the +6, +5, +4 and +3 peaks ; b) Expanded view of the +4 peaks ; c) Observed (blue) and theoretical (red) isotope patterns for the +4 peak of the **G1⊂1**·$2F^{-}$ complex.

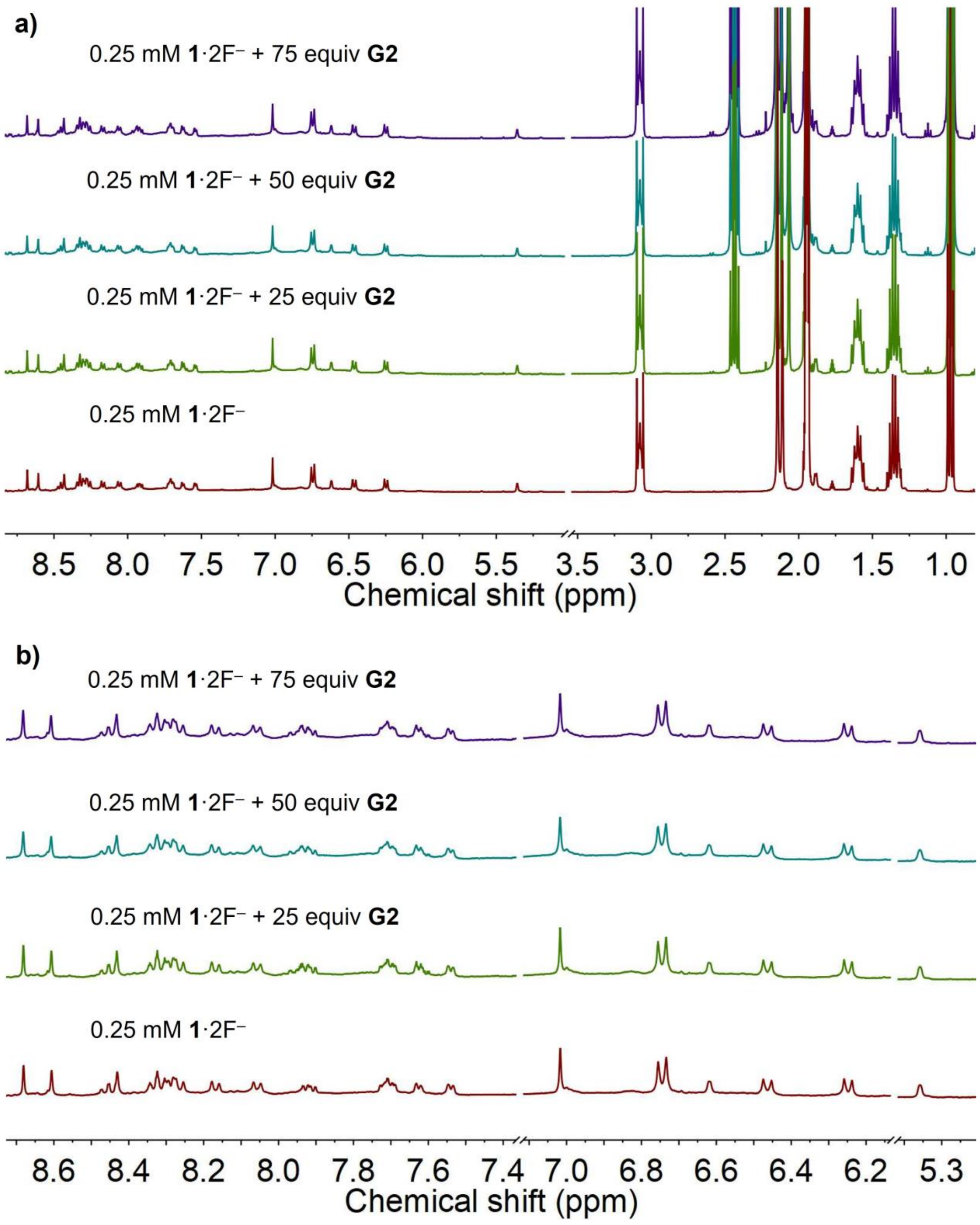

Figure S55. ^{1}H NMR spectra (500 MHz, CD_3CN, 298 K) of a) 0.25 mM **1**·$2F^{-}$ with different amounts of **G2** (2-butanone, 25 equiv, 50 equiv and 75 equiv) ; b) Expanded view of the aromatic region of a). No peaks were observed for **G2⊂1**·$2F^{-}$, indicating a binding constant below 1.

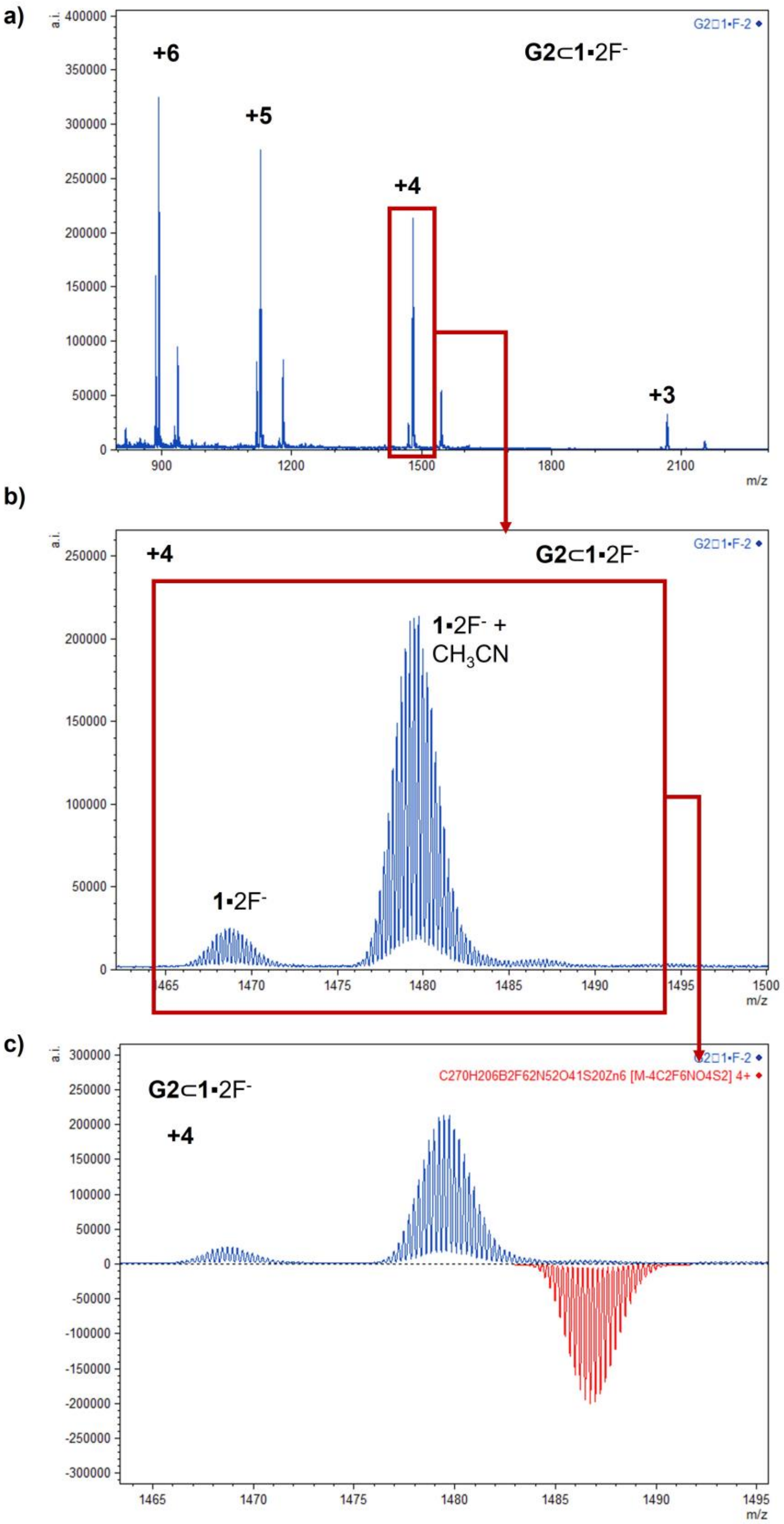

Figure S56. a) High-resolution ESI-mass spectrometry analysis of **1**·2F⁻ mixed with 75 equiv **G2**, showing the +6, +5, +4 and +3 peaks ; b) Expandd view of the +4 peaks ; c) Observed (blue) data and theoretical (red) isotope patterns for the +4 peak of the hypothetical host-guest complex. No clear signal was observed for **G2⊂1**·2F⁻, which is consistent with the NMR results.

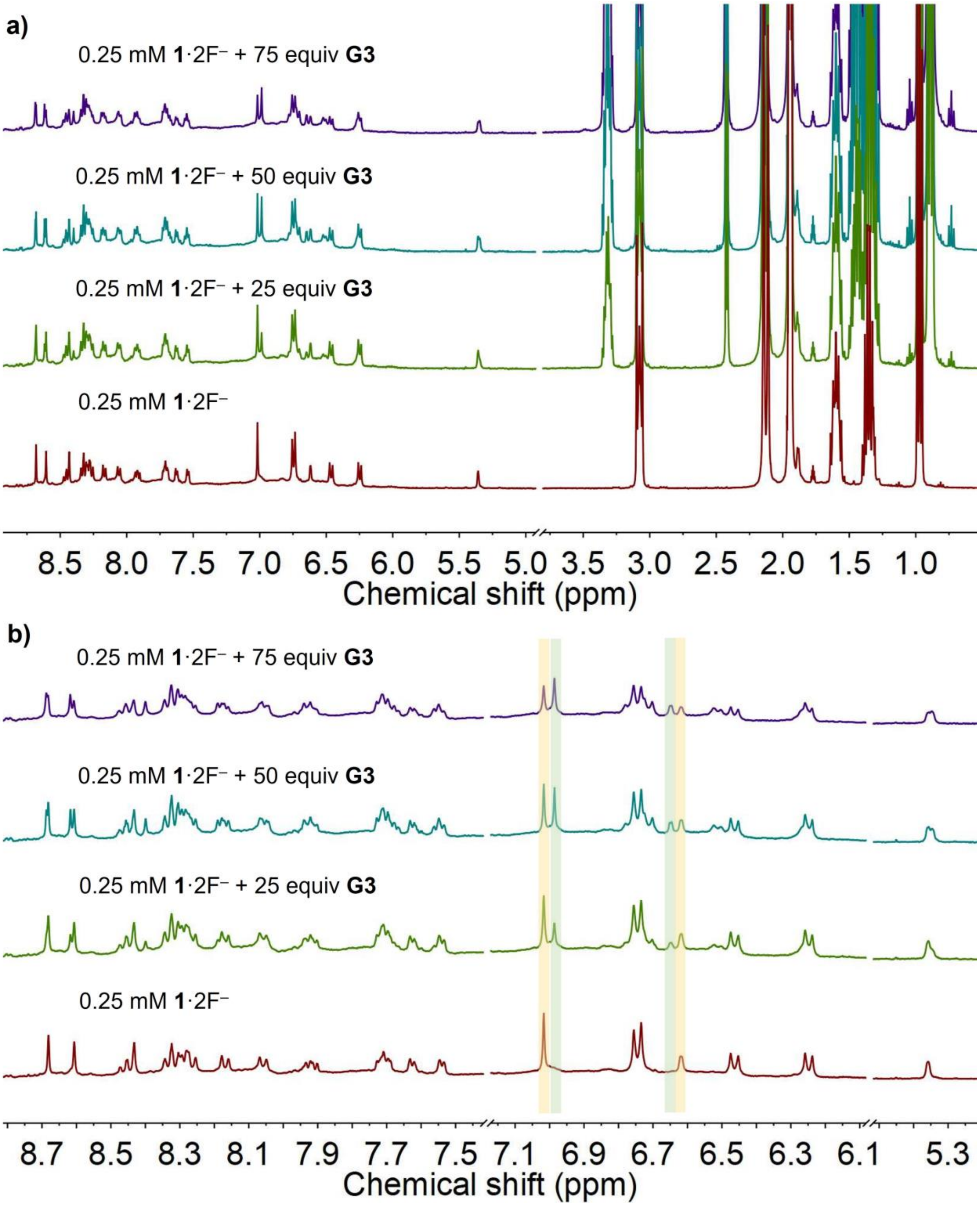

Figure S57. ^{1}H NMR spectra (500 MHz, CD_3CN, 298 K) of a) 0.25 mM **1**·$2F^–$ with different amounts of **G3** (3-pentanol, 25 equiv, 50 equiv and 75 equiv) ; b) Expanded view of the aromatic region of a). Selected peaks for **G3⊂1**·$2F^–$ and free **1**·$2F^–$ are highlighted by light green and light yellow shading respectively. The binding constant was calculated to be 210 ± 35 M^{-1}

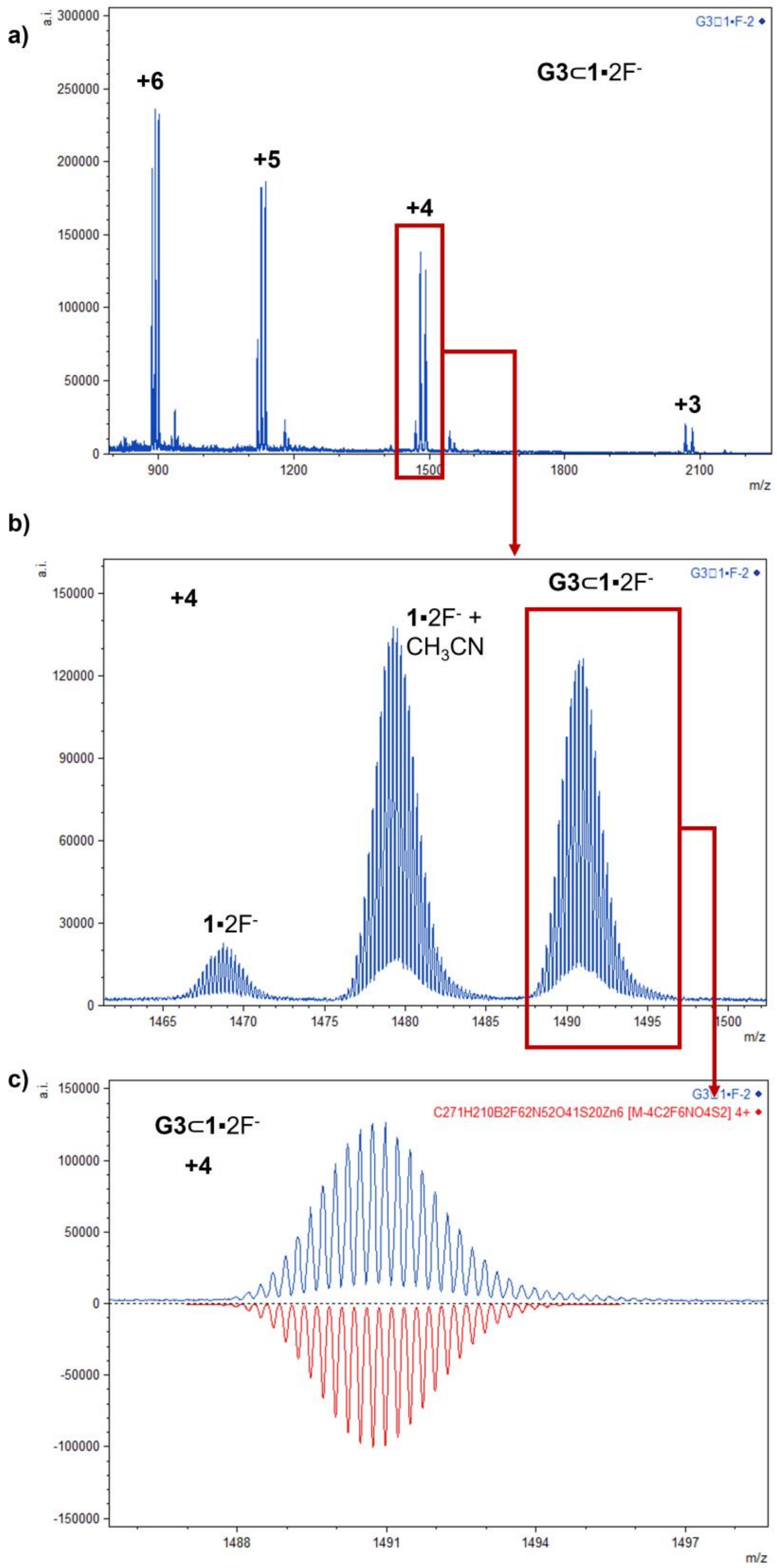

Figure S58. a) High-resolution ESI-mass spectrometry analysis of **1**·F^{-}_{2} mixed with 75 equiv **G3**, showing the +6, +5, +4 and +3 peaks ; b) Expanded view of at the +4 peaks ; c) Observed (blue) and theoretical (red) isotope patterns for the +4 peak of the **G3⊂1**·$2F^{-}$ complex.

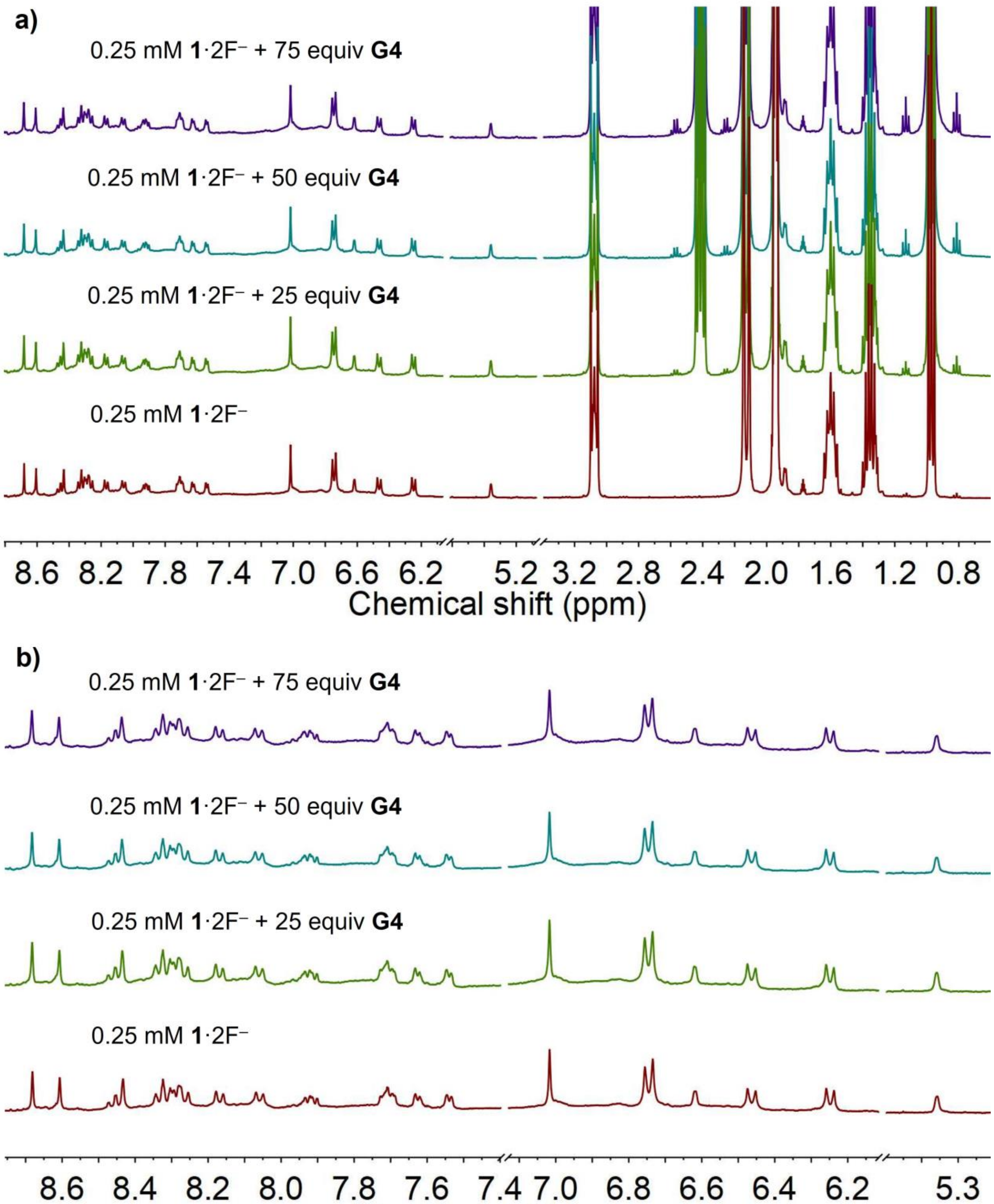

Figure S59. ^{1}H NMR spectra (500 MHz, CD_3CN, 298 K) of a) 0.25 mM **1**·$2F^–$ with different amounts of **G4** (3-pentanone, 25 equiv, 50 equiv and 75 equiv) ; b) Expanded view of the aromatic region of a). No peaks were observed for **G4⊂1**·$2F^–$, indicating a binding constant below 1.

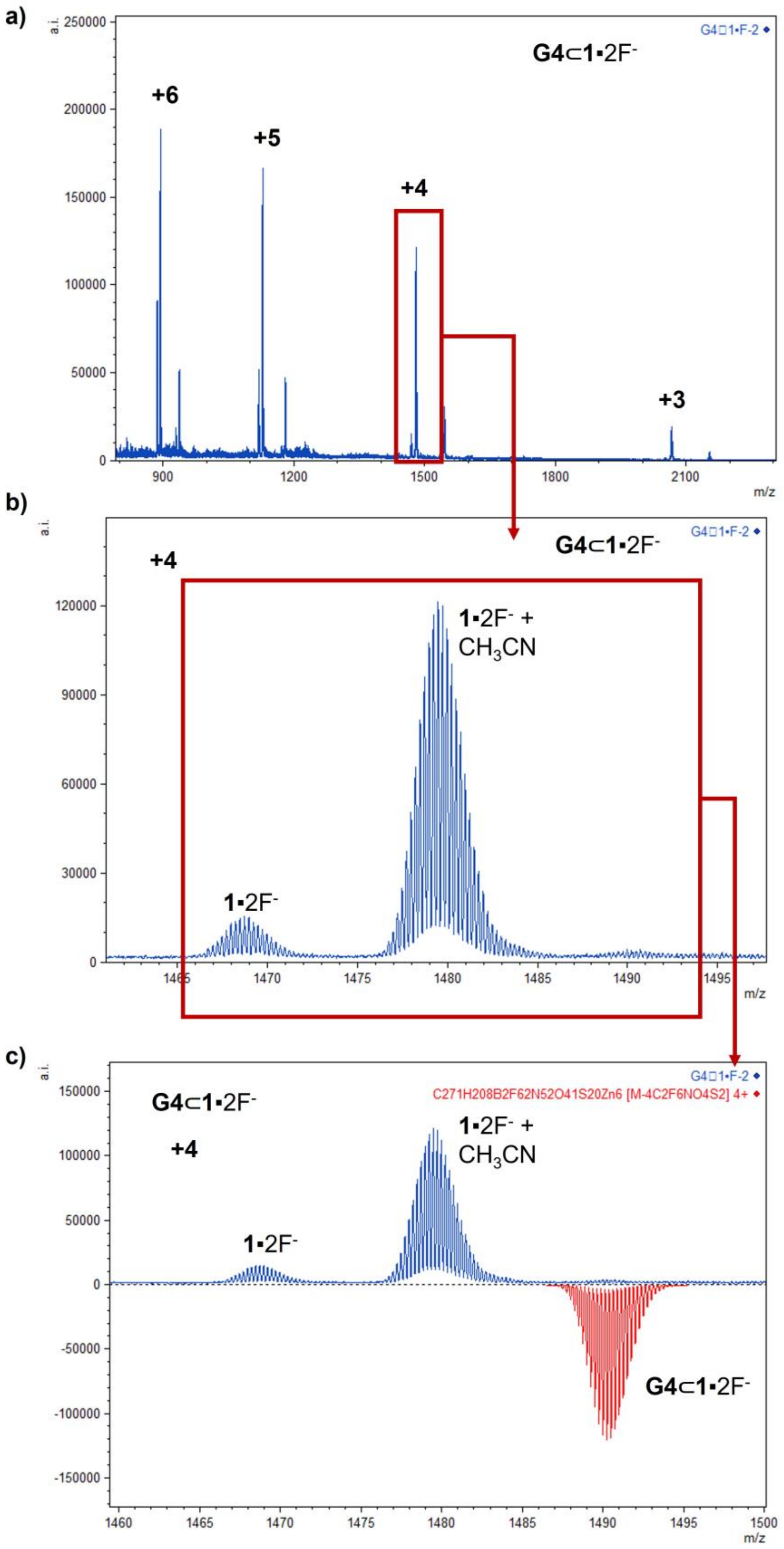

Figure S60. a) High-resolution ESI-mass spectrometry analysis of **1**·2F⁻ mixed with 75 equiv **G4**, showing the +6, +5, +4 and +3 peaks ; b) Expanded view of the +4 peaks ; c) Observed (blue) data and theoretical (red) isotope patterns for the +4 peak of the hypothetical host-guest complex. No clear signal was observed for **G4⊂1**·2F⁻, which is consistent with the NMR results.

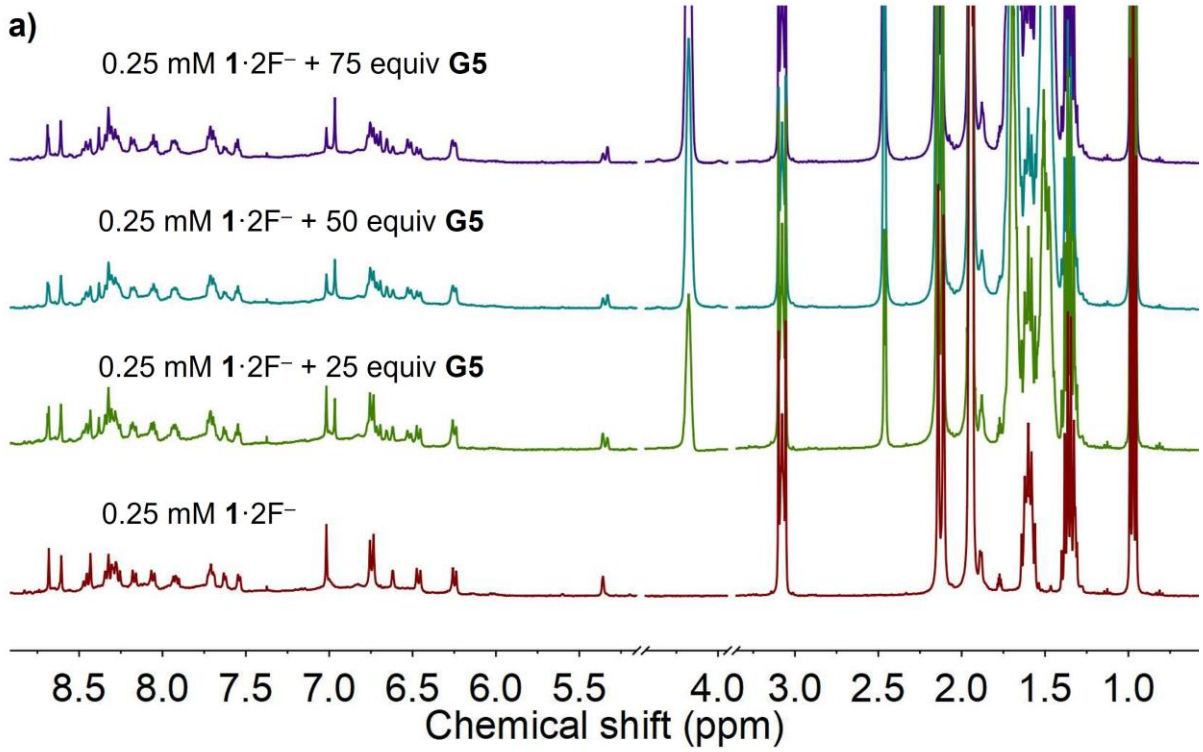

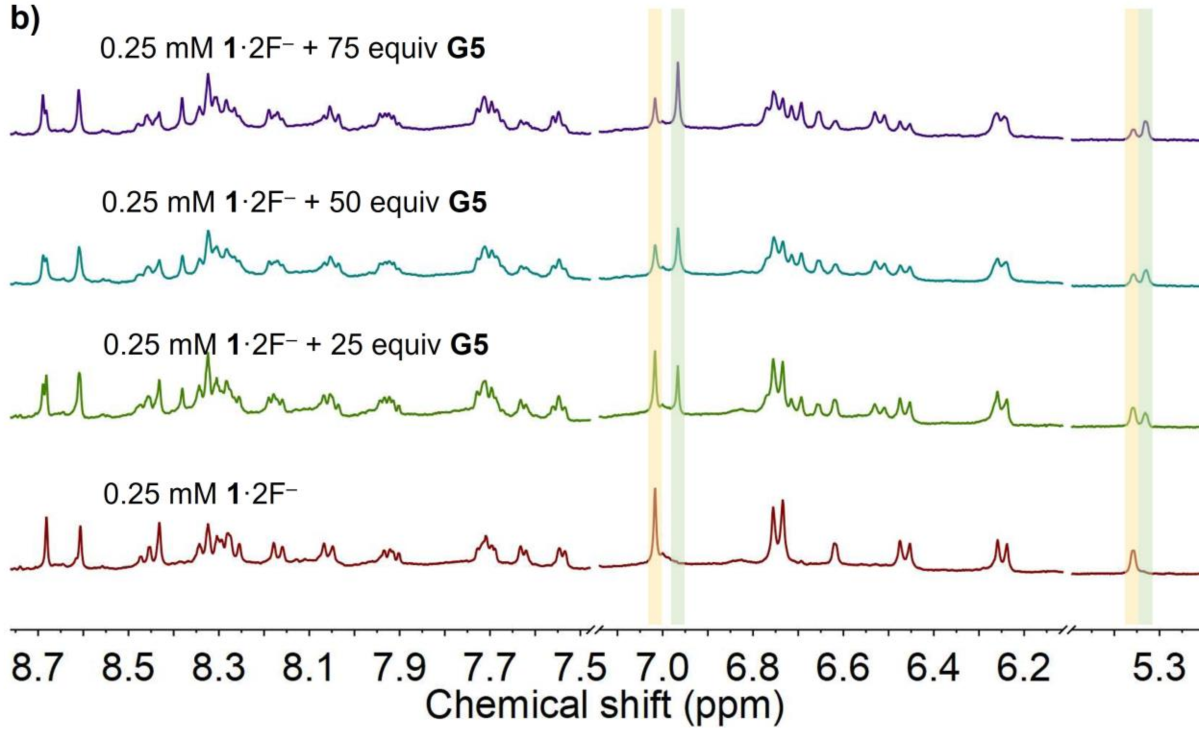

Figure S61. ^{1}H NMR spectra (500 MHz, CD_3CN, 298 K) of a) 0.25 mM **1**·$2F^-$ with different amounts of **G5** (cyclopentanol, 25 equiv, 50 equiv and 75 equiv) ; b) Expanded view of the aromatic region of a). Selected peaks for **G5⊂1**·$2F^-$ and free **1**·$2F^-$ are highlighted by light green and light yellow shading respectively. The binding constant was calculated to be 286 ± 48 M^{-1}.

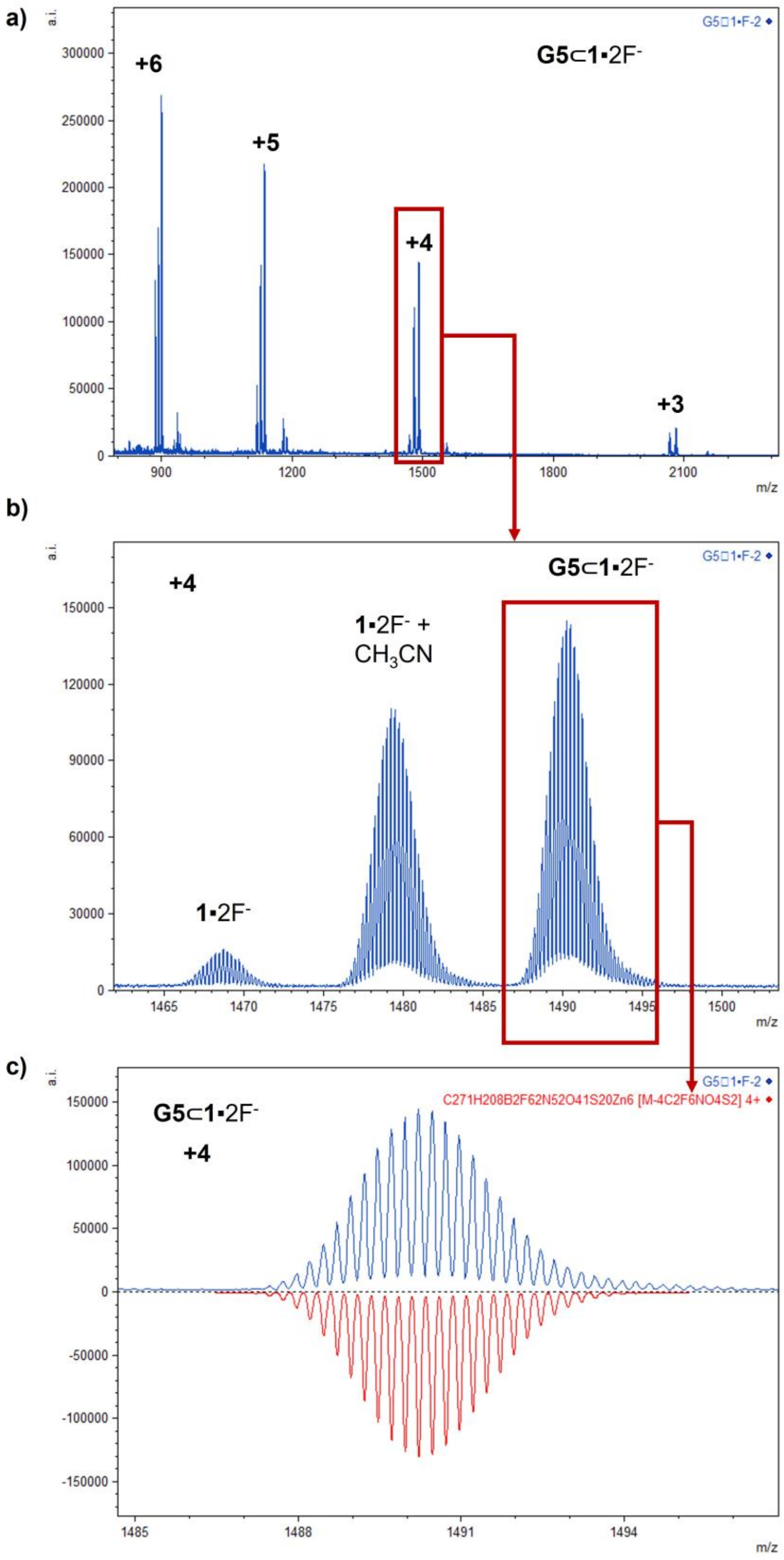

Figure S62. a) High-resolution ESI-mass spectrometry analysis of **1**·2F⁻ mixed with 75 equiv **G5**, showing the +6, +5, +4 and +3 peaks ; b) Expand view of the +4 peaks ; c) Observed (blue) and theoretical (red) isotope patterns for the +4 peak of the **G5⊂1**·2F⁻ complex.

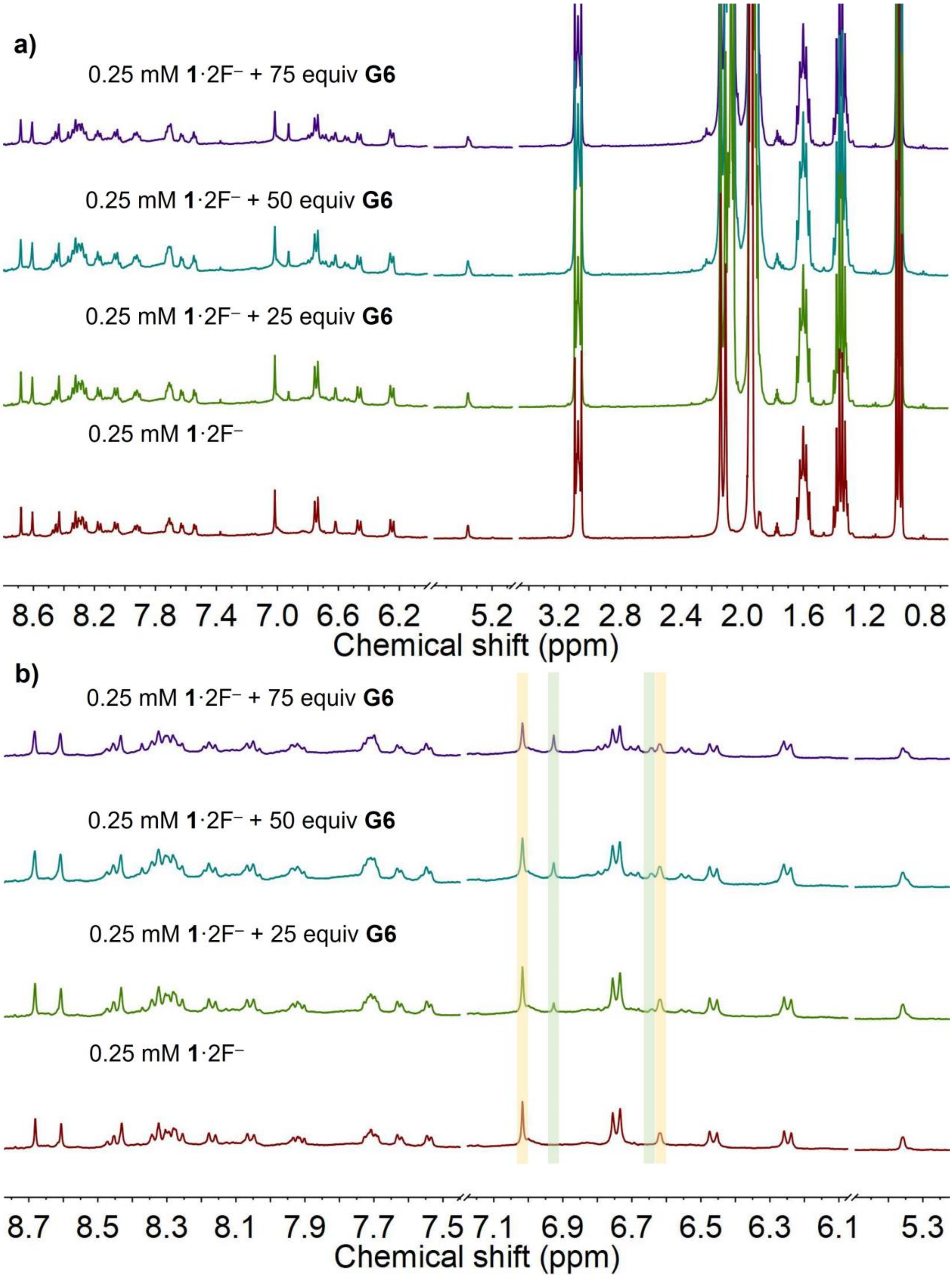

Figure S63. ^{1}H NMR spectra (500 MHz, CD_3CN, 298 K) of a) 0.25 mM **1**·$2F^-$ with different amounts of **G6** (cyclopentanone, 25 equiv, 50 equiv and 75 equiv) ; b) Expanded view of the aromatic region of a). Selected peaks for **G6**⊂**1**·$2F^-$ and free **1**·$2F^-$ are highlighted by light green and light yellow shading respectively. The binding constant was calculated to be 52.5 ± 8.9 M^{-1}.

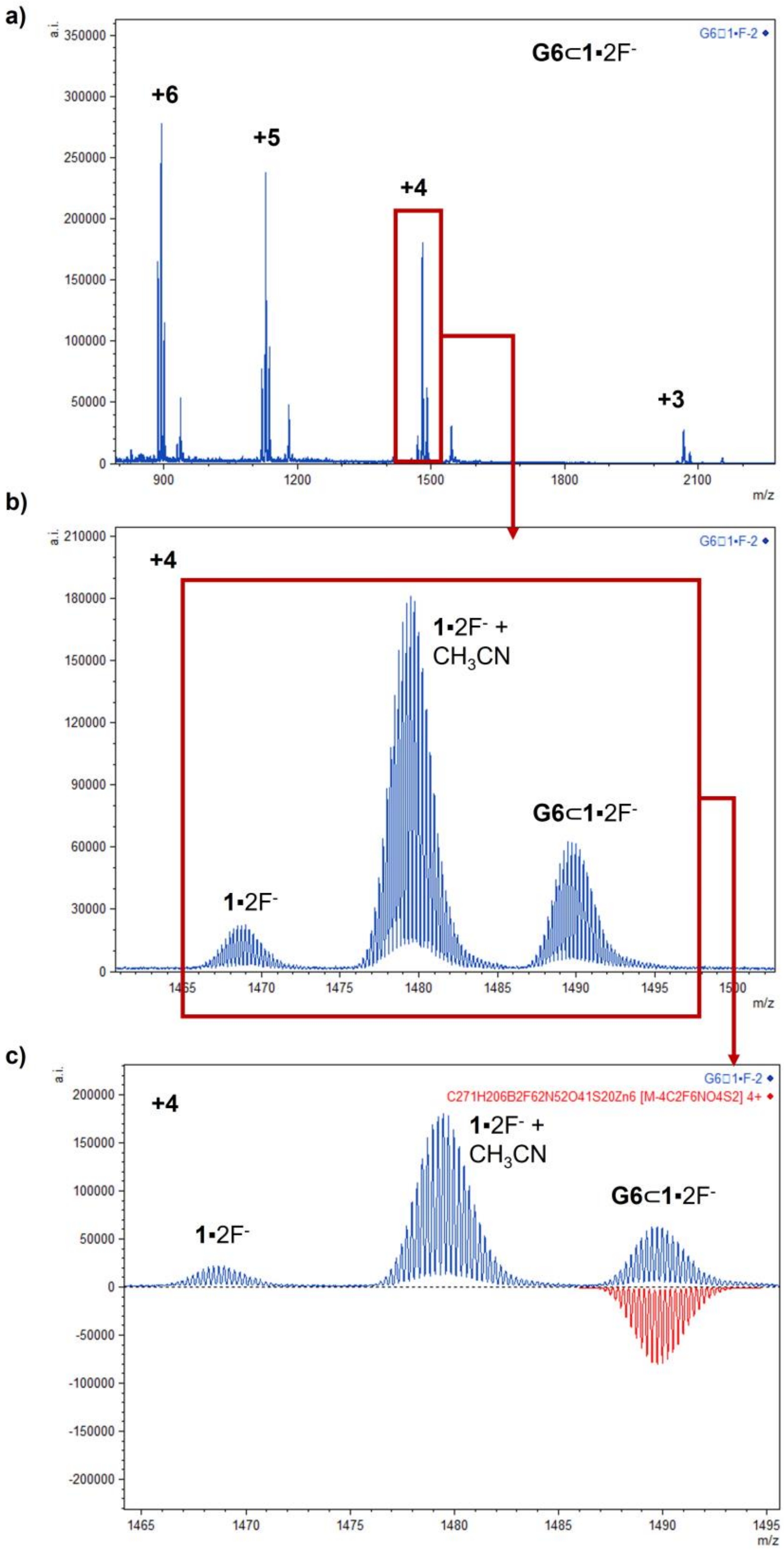

Figure S64. a) High-resolution ESI-mass spectrometry analysis of **1**·2F⁻ mixed with 75 equiv **G6**, showing the +6, +5, +4 and +3 peaks ; b) Expanded view the +4 peaks ; c) Observed (blue) and theoretical (red) isotope patterns for the +4 peak of the **G6⊂1**·2F⁻ complex.

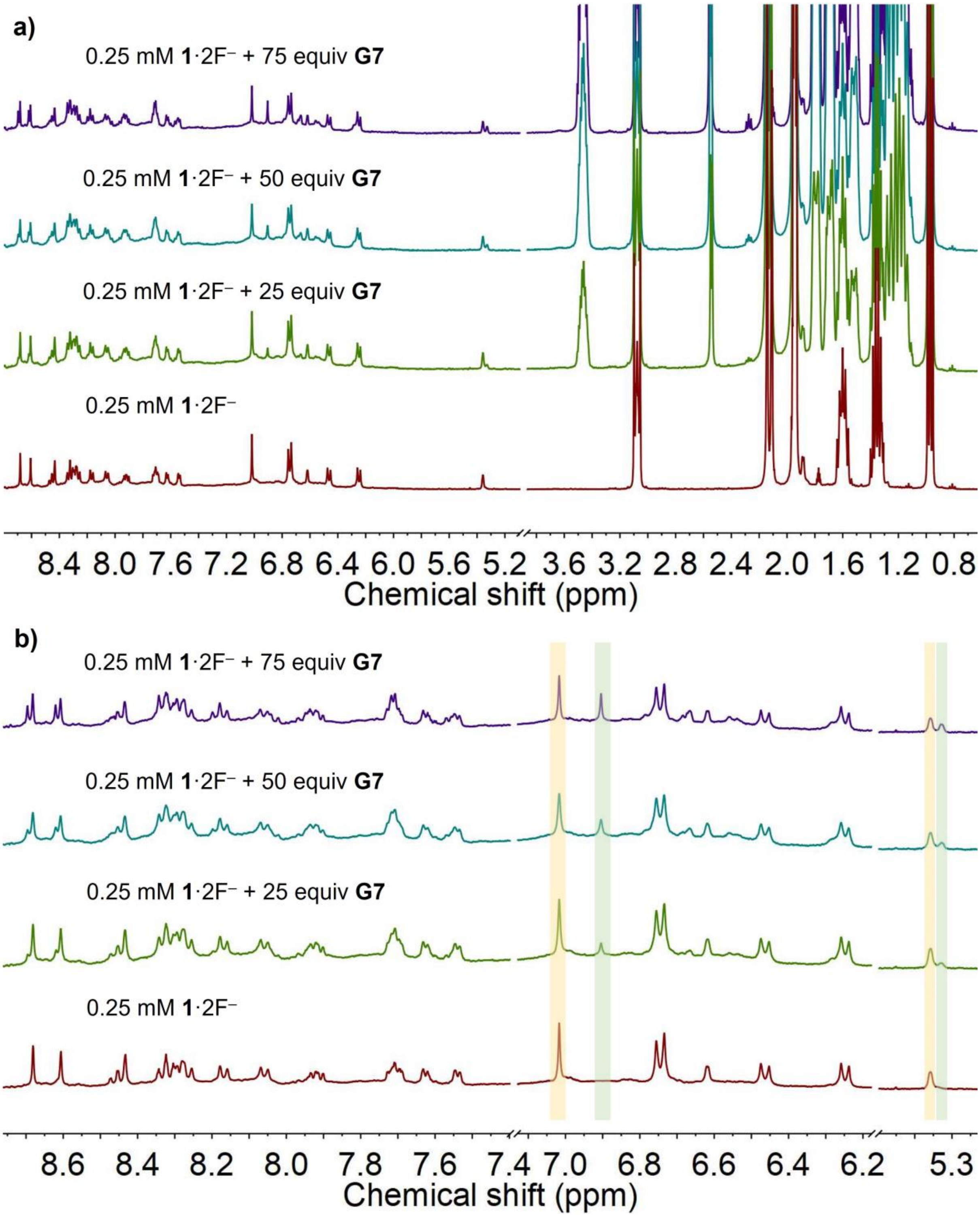

Figure S65. ^{1}H NMR spectra (500 MHz, CD_3CN, 298 K) of a) 0.25 mM **1**·2F⁻ with different amounts of **G7** (cyclohexanol, 25 equiv, 50 equiv and 75 equiv); b) Expanded view of the aromatic region of a). Selected peaks for **G7⊂1**·2F⁻ and free **1**·2F⁻ are highlighted by light green and light yellow shading respectively. The binding constant was calculated to be 79.4 ± 8.8 M^{-1}.

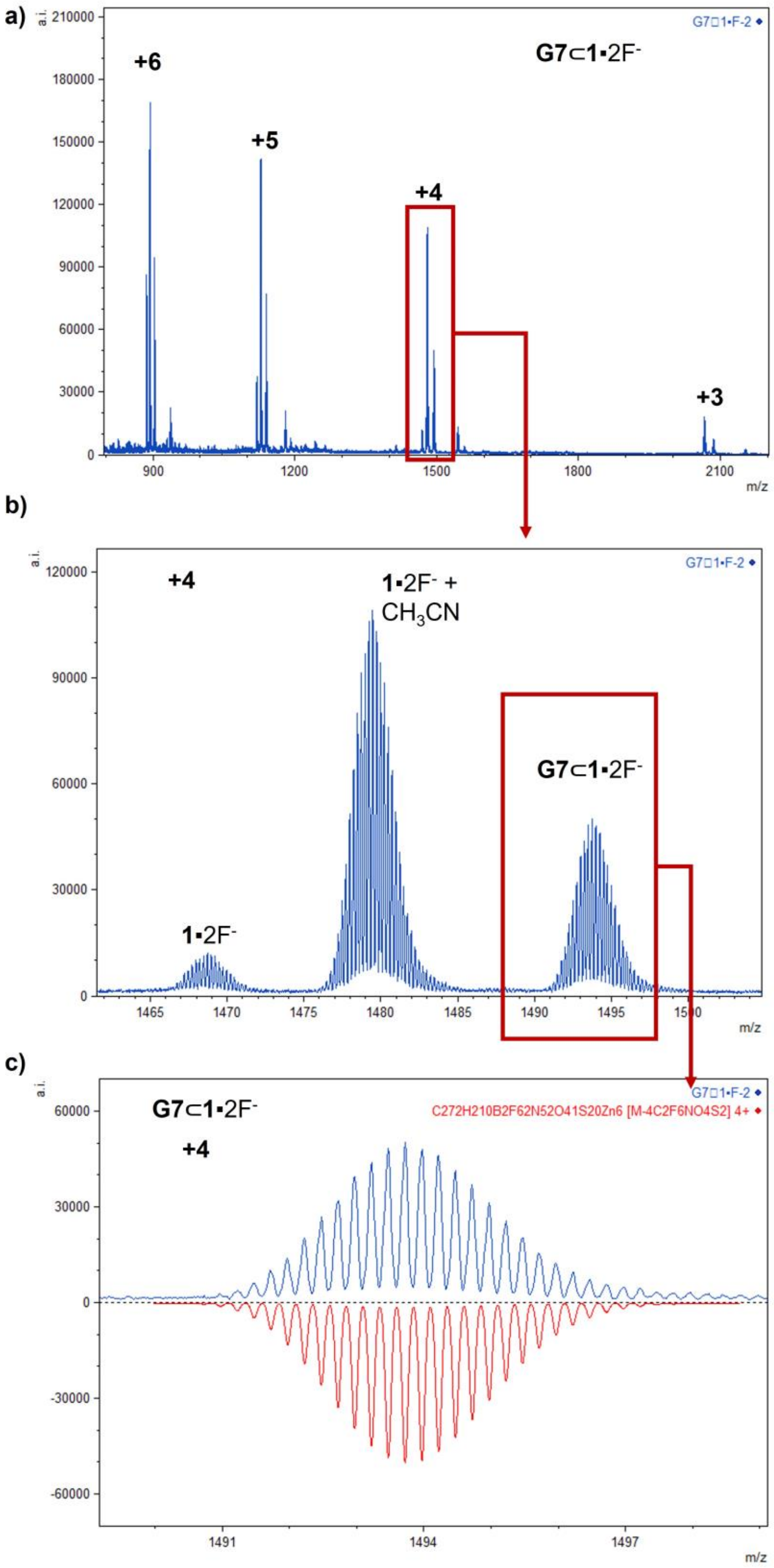

Figure S66. a) High-resolution ESI-mass spectrometry analysis of **1**·2F⁻ mixed with 75 equiv **G7**, showing the +6, +5, +4 and +3 peaks ; b) Expanded view the +4 peaks ; c) Observed (blue) and theoretical (red) isotope patterns for the +4 peak of the **G7⊂1**·2F⁻ complex.

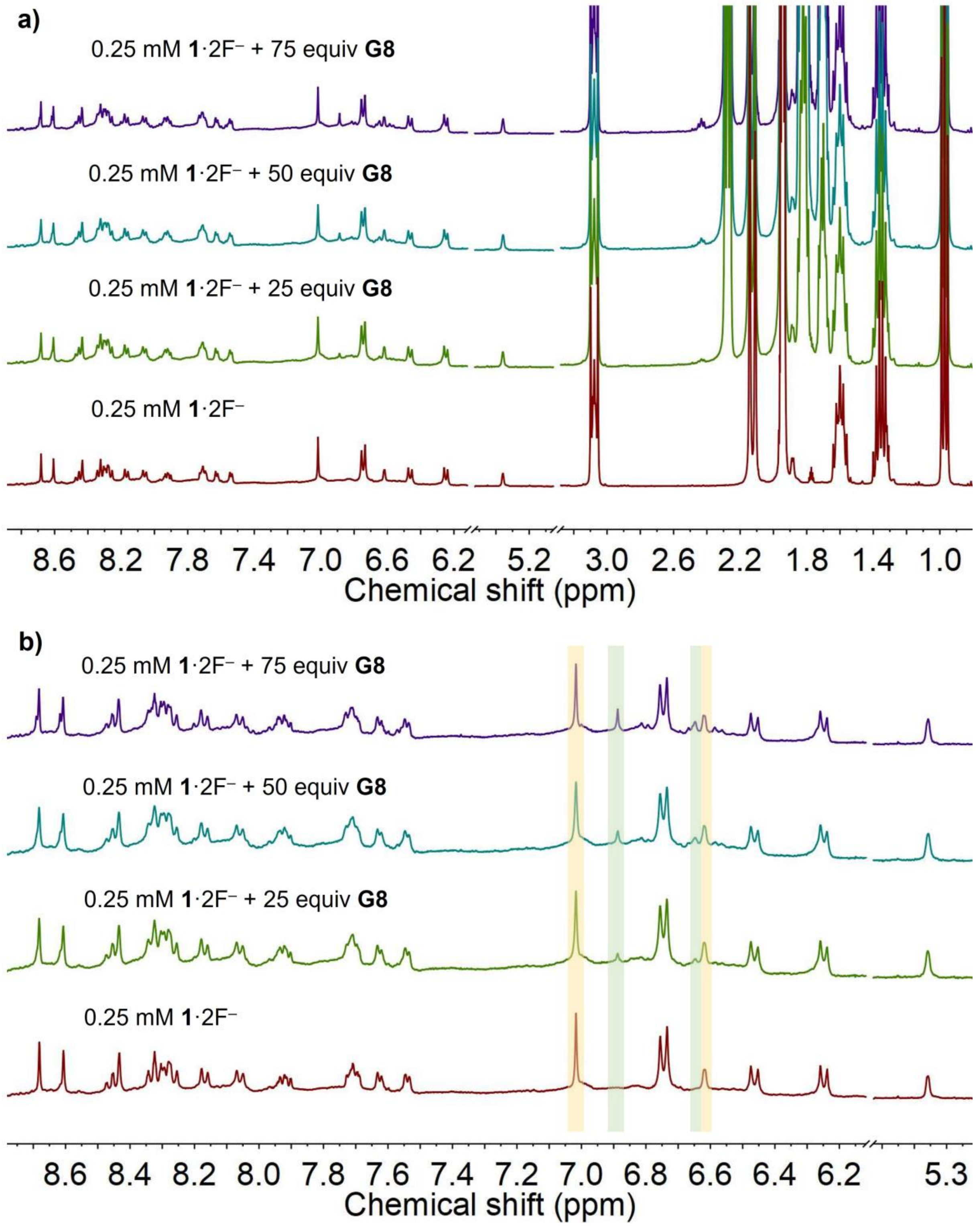

Figure S67. ^{1}H NMR spectra (500 MHz, CD_3CN, 298 K) of a) 0.25 mM **1**·$2F^{-}$ with different amounts of **G8** (cyclohexanone, 25 equiv, 50 equiv and 75 equiv) ; b) Expanded view of the aromatic region of a). Selected peaks for **G8**⊂**1**·$2F^{-}$ and free **1**·$2F^{-}$ are highlighted by light green and light yellow shading respectively. The binding constant was calculated to be 19.5 ± 4.9 M^{-1}.

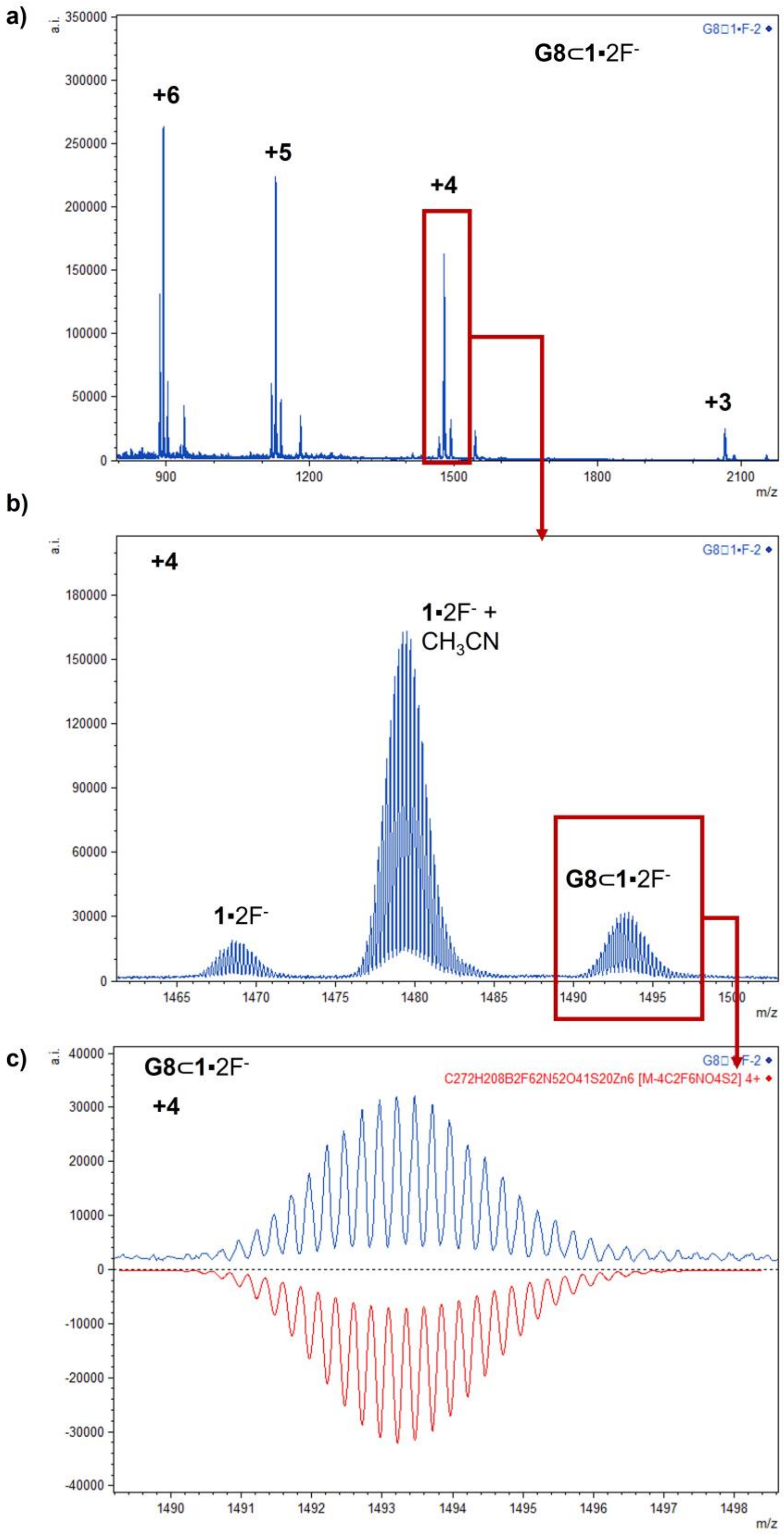

Figure S68. a) High-resolution ESI-mass spectrometry analysis of **1**·2F⁻ mixed with 75 equiv **G8**, showing the +6, +5, +4 and +3 peaks ; b) Expand view of the +4 peaks ; c) Observed (blue) and theoretical (red) isotope patterns for the +4 peak of the **G8⊂1**·2F⁻ complex.

3.4 Host-guest interactions of 1 and 1·2F⁻ with different guest molecules (G9-G14)

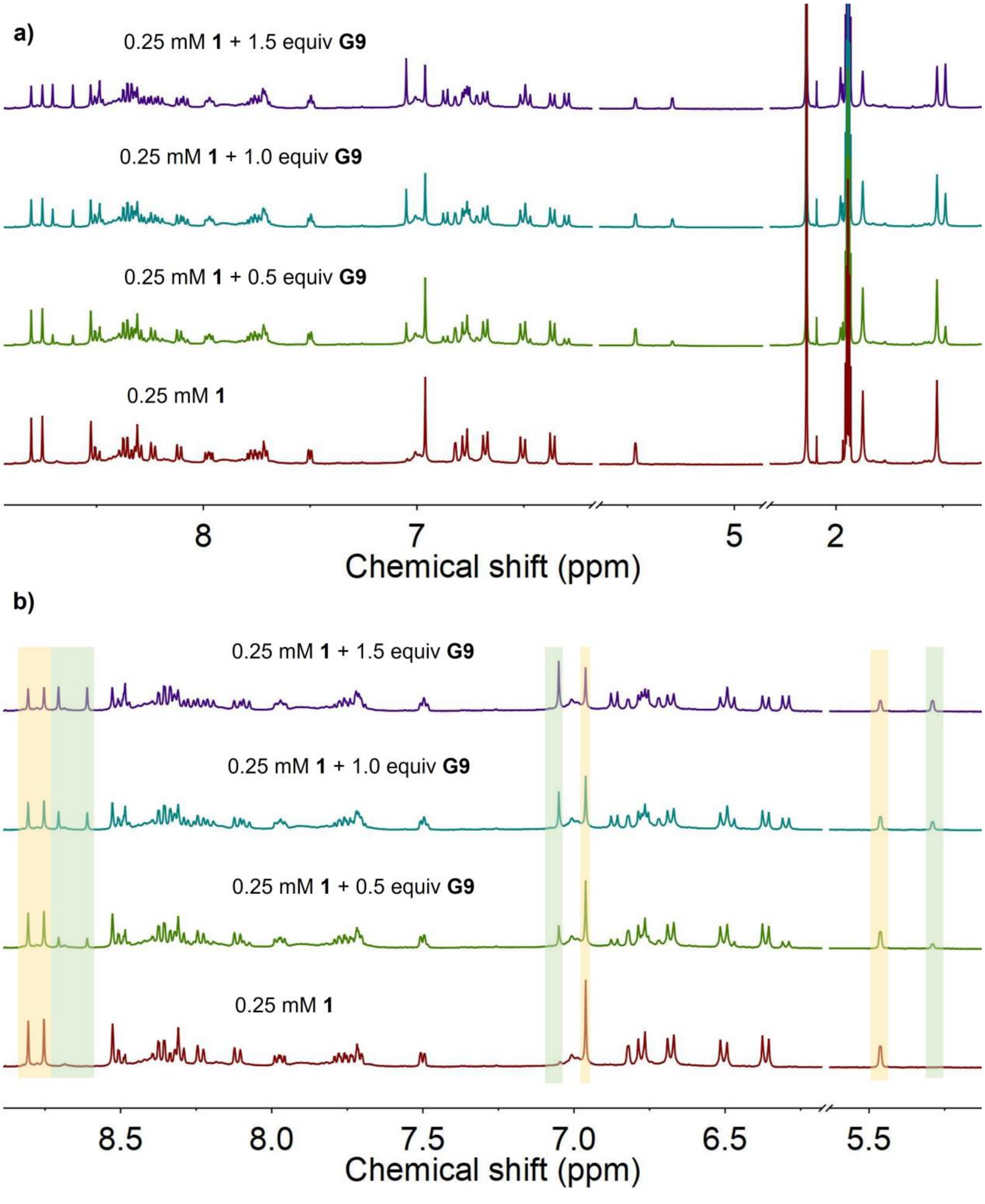

Figure S69. ^{1}H NMR spectra (500 MHz, CD_3CN, 298 K) of a) 0.25 mM **1** with different amounts of **G9** ($KSbF_6$, 0.5 equiv, 1.0 equiv, 1.5 equiv); b) Selected peaks for the **G9⊂1** and free **1** are highlighted by light green and light yellow shading respectively. The binding constants was calculated to be $(7.05 \pm 0.72) \times 10^3$ M^{-1}.

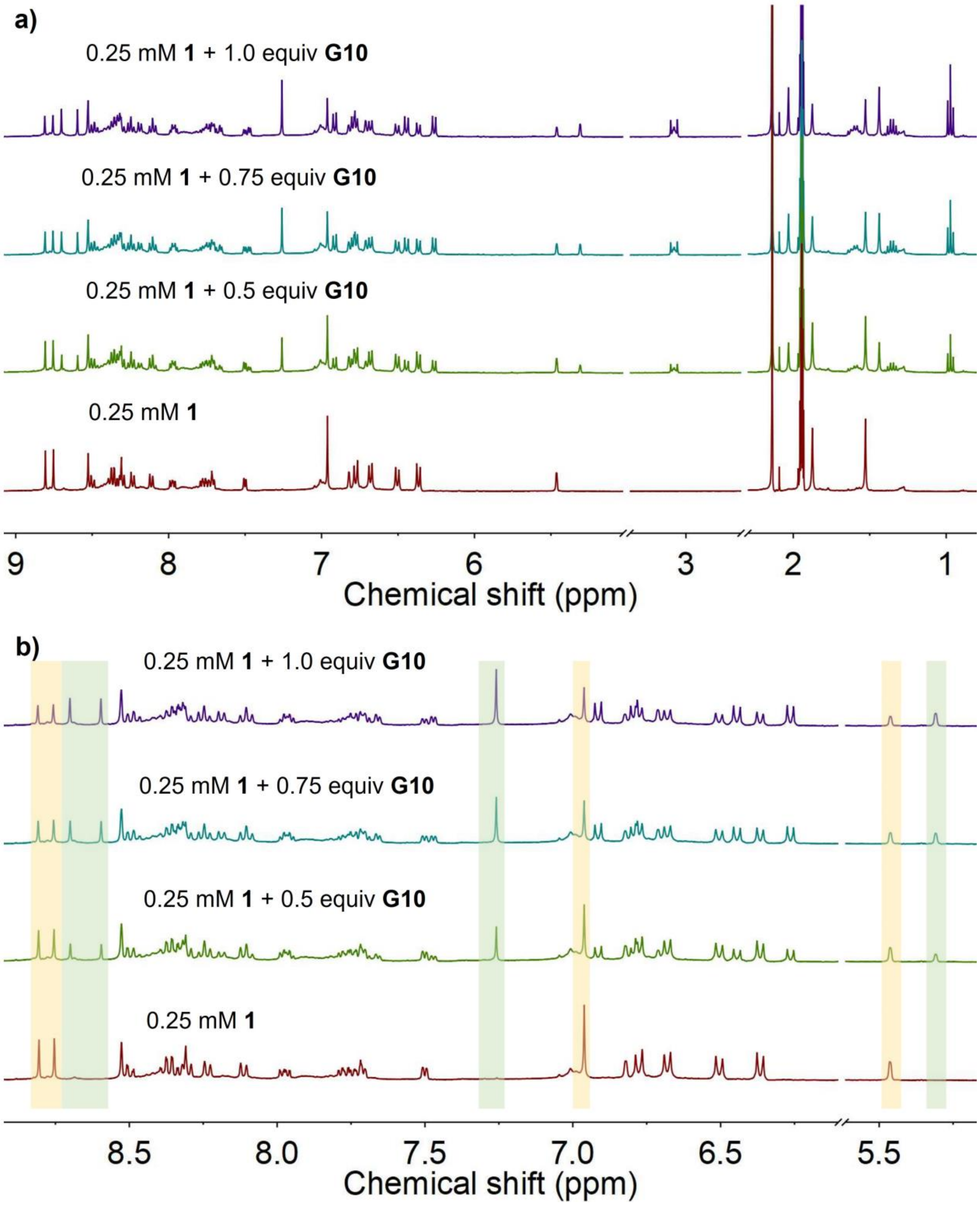

Figure S70. ^{1}H NMR spectra (500 MHz, CD_3CN, 298 K) of a) 0.25 mM **1** with different amounts of **G10** ($TBAReO_4$, 0.5 equiv, 0.75 equiv, 1.0 equiv); b) Selected peaks for the **G10⊂1** and free **1** are highlighted by light green and light yellow shading respectively. The binding constants was calculated to be $(1.31 \pm 0.14) \times 10^4\ M^{-1}$.

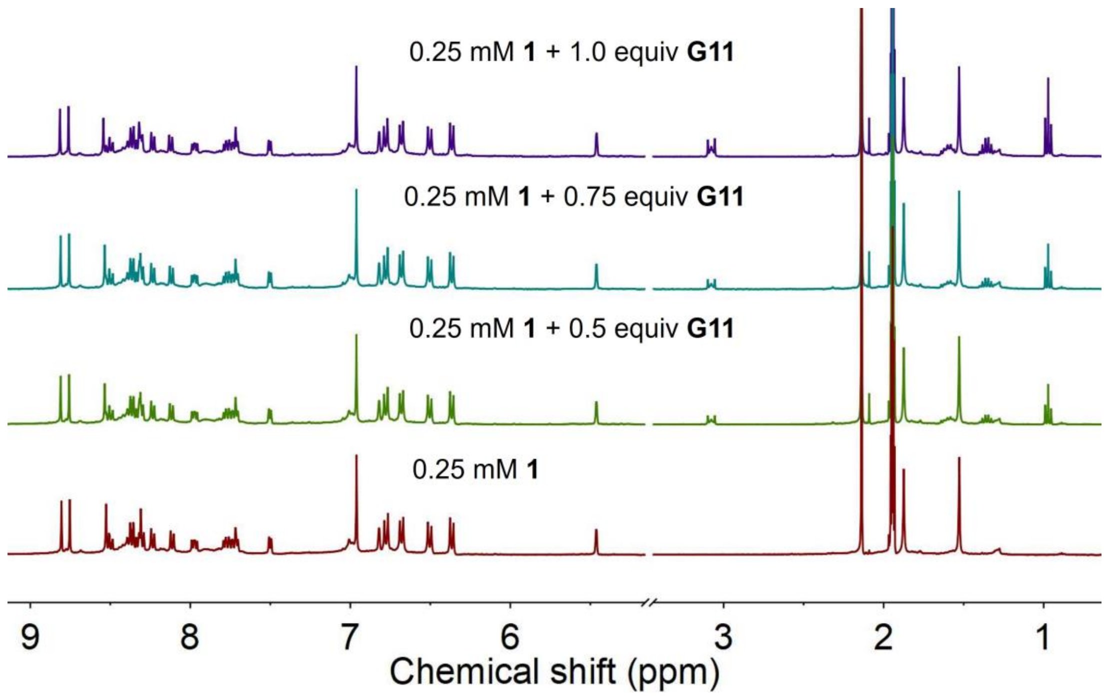

Figure S71. ^{1}H NMR spectra (500 MHz, CD_3CN, 298 K) of 0.25 mM **1** with different amounts of **G11** ($TBAClO_4$, 0.5 equiv, 0.75 equiv, 1.0 equiv), no new peaks were observed for **G11⊂1**, in contrast to **G10⊂1** shown in Figure S70.

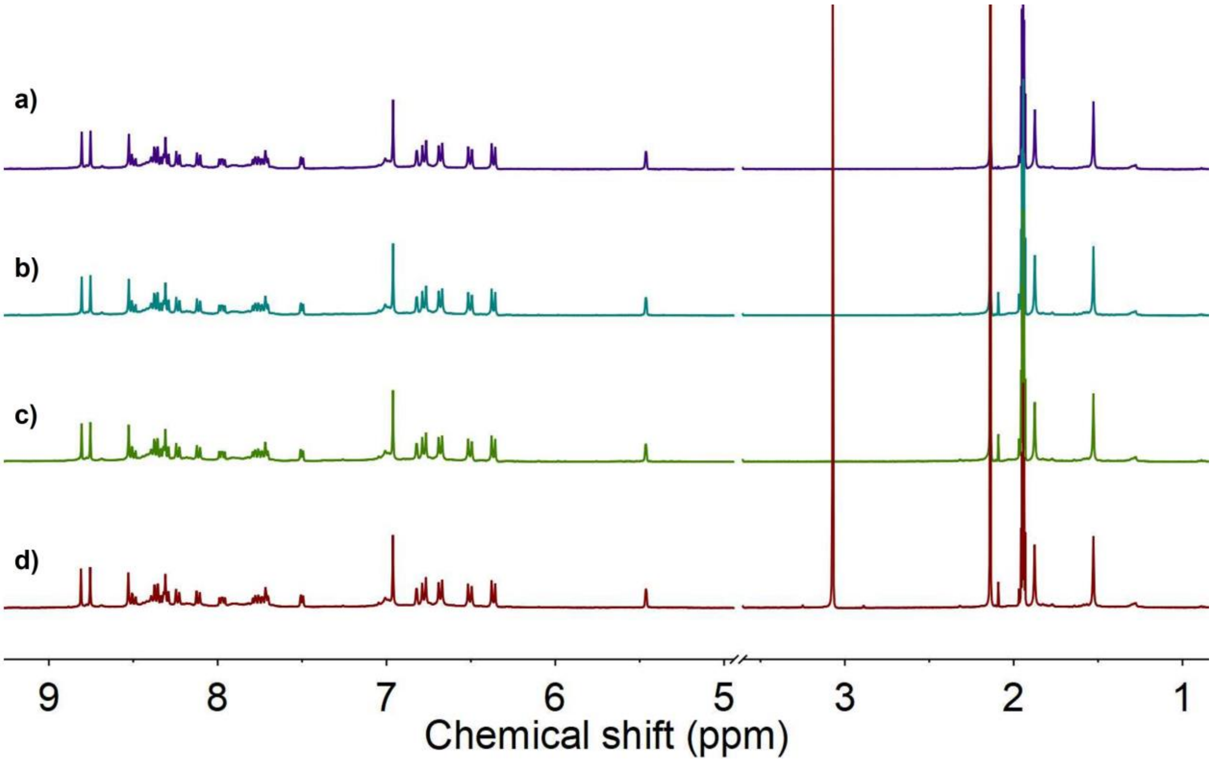

Figure S72. ^{1}H NMR spectra (500 MHz, CD_3CN, 298 K) of 0.25 mM **1** with a) 20 equiv **G12** (hexafluorobenzene); b) 20 equiv **G13** (octafluorocyclopentene) and c) 2.5 equiv **G14** (tetramethylammonium bis(trifluoromethanesulfonyl)imide), no peaks indicative of host-guest binding were observed.

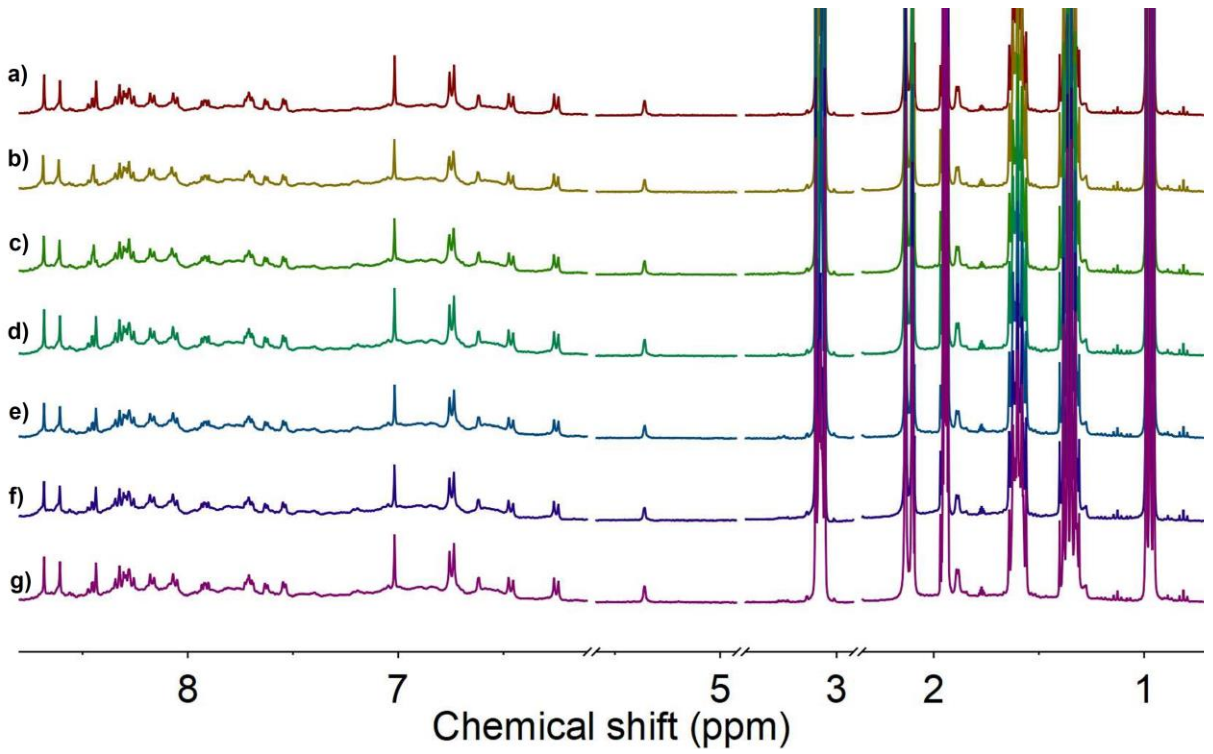

Figure S73. ^{1}H NMR spectra (500 MHz, CD_3CN, 298 K) of 0.25 mM **1**$\cdot 2F^-$ with a) control group; b) 1 equiv **G9** ($KSbF_6$); c) 1 equiv **G10** ($TBAReO_4$); d) 1 equiv **G11** ($TBAClO_4$); e) 20 equiv **G12** (hexafluorobenzene); f) 20 equiv **G13** (octafluorocyclopentene) and g) 2.5 equiv **G14** (tetramethylammonium bis(trifluoromethanesulfonyl)imide), no peaks indicative of host-guest binding were observed

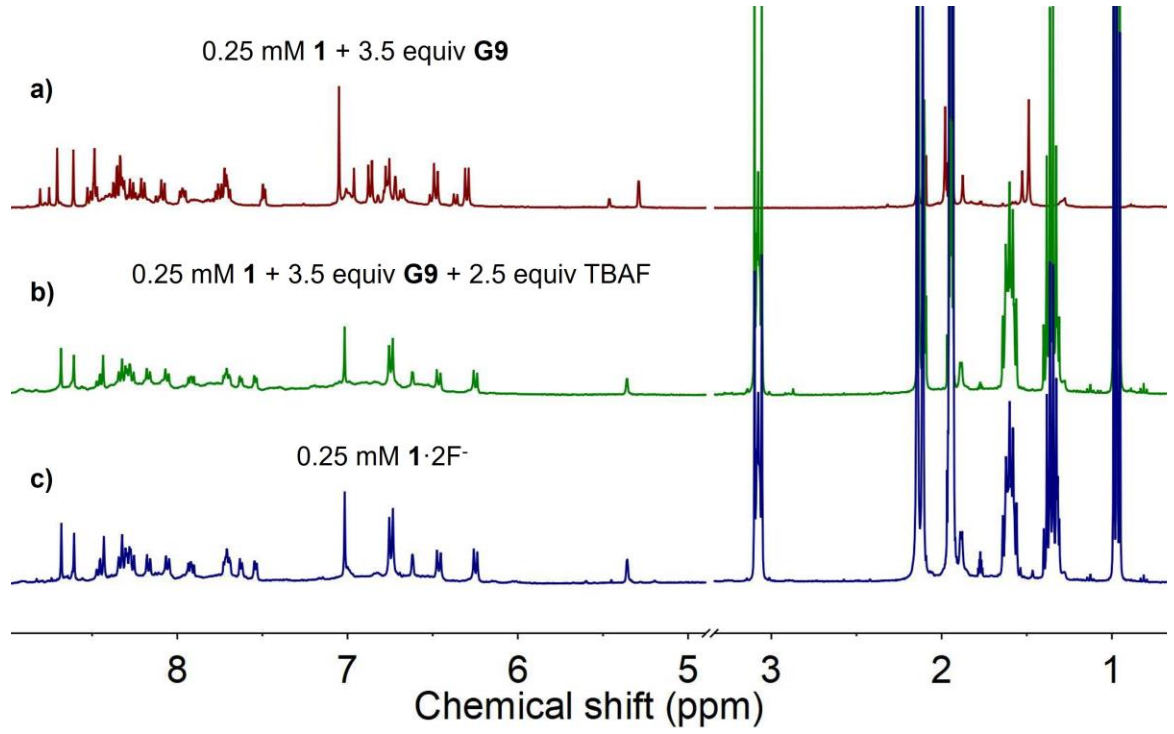

Figure S74. ^{1}H NMR spectra (500 MHz, CD_3CN, 298 K) of a) 0.25 mM **1** with 3.5 equiv **G9**; b) a) with addition of 2.5 equiv TBAF ; c) Control group of 0.25 mM **1**$\cdot 2F^-$. Cage signals in b) and c) appear at the same position, indicating the ejection of **G9** from the cavity.

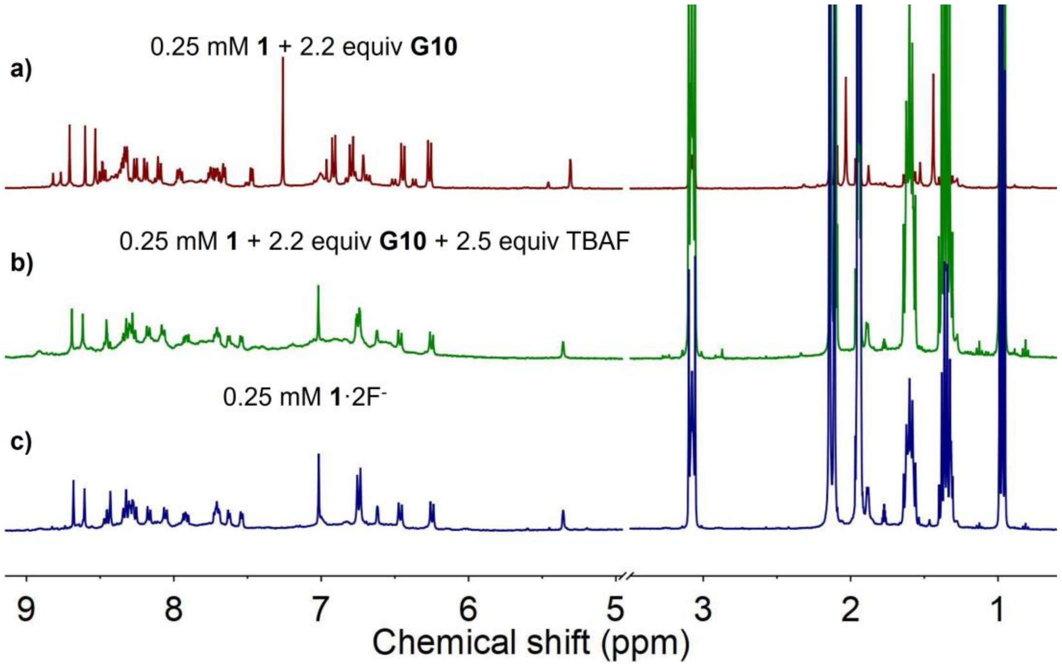

Figure S75. ^{1}H NMR spectra (500 MHz, CD_3CN, 298 K) of a) 0.25 mM **1** with 2.2 equiv **G10**; b) a) with addition of 2.5 equiv TBAF ; c) Control group of 0.25 mM **1**·$2F^{-}$. Cage signals in b) and c) appear at the same position, indicating the ejection of **G10** from the cavity.

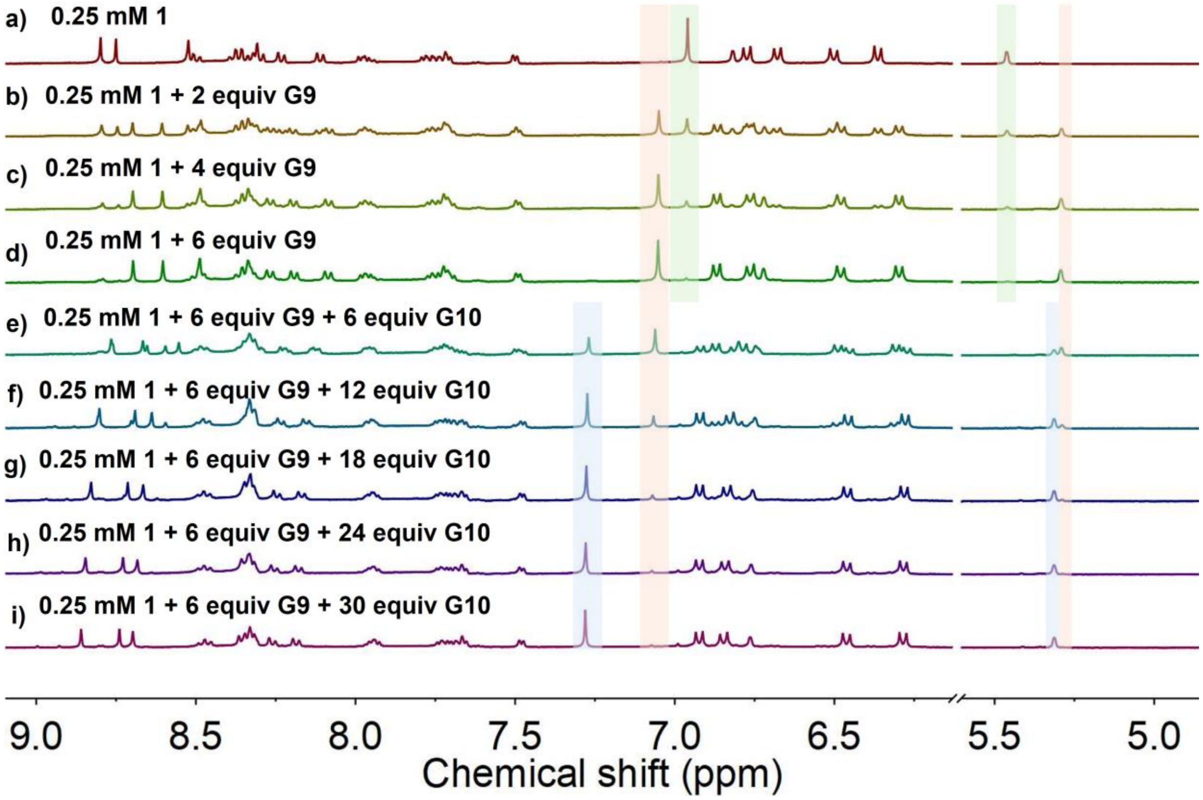

Figure S76. ^{1}H NMR spectra (500 MHz, CD_3CN, 298 K) of competition titration of **G9** (SbF_6^{-}) and **G10** (ReO_4^{-}). a) 0.25 mM **1**; b) 0.25 mM with 2 equiv **G9**; c) 0.25 mM with 4 equiv **G9**; d) 0.25 mM with 6 equiv **G9**; e) d) with addition of 6 equiv **G10**; f) d) with addition of 12 equiv **G10**; g) d) with addition of 18 equiv **G10**; h) d) with addition of 24 equiv **G10**; i) d) with addition of 30 equiv **G10**. Selected peaks for free **1**, **G9⊂1** and **G10⊂1** are highlighted by light green, light orange and light blue shading respectively.

4 X-ray crystallography

The crystals of $[Zn_6L^A{}_3L^C{}_2]\cdot 12NTf_2\cdot CH_3CN$ [+ solvent] were grown by diffusion of diethyl ether into an acetonitrile solution of the complex. Data were collected at Beamline I19 of Diamond Light Source employing silicon double crystal monochromated synchrotron radiation (0.6889 Å) with ω and ψ scans at 100(2) K.[9] Data integration and reduction were undertaken with Xia2.[10–12] Subsequent computations were carried out using the WinGX-32 graphical user interface.[13] Multi-scan empirical absorption corrections were applied to the data using the AIMLESS[14] tool in the CCP4 suite.[15] The structures were solved by direct methods using SHELXT[16] then refined and extended with SHELXL.[17] In general, non-hydrogen atoms with occupancies greater than 0.5 were refined anisotropically. Carbon-bound hydrogen atoms were included in idealised positions and refined using a riding model. Disorder was modelled using standard crystallographic methods including constraints, restraints and rigid bodies where necessary. Crystallographic data along with specific details pertaining to the refinement follow. Crystallographic data have been deposited with the CCDC (2407241).

The crystals employed immediately lost solvent after removal from the mother liquor and rapid handling prior to flash cooling in liquid nitrogen was required to collect data. Despite these measures and the use of synchrotron radiation few reflections at greater than 1.0 Å resolution were observed and the data were trimmed accordingly. Nevertheless, the quality of the data is far more than sufficient to establish the connectivity of the structure. The asymmetric unit was found to contain one complete $Zn_6L^A{}_3L^C{}_2$ assembly and associated counterions and solvent molecules. Due to the limited resolution bond lengths and angles within pairs of chemically identical organic ligands were restrained to be similar to each other and thermal parameter restraints (SIMU, RIGU) were applied to all atoms except for zinc.

The anions within the structure show evidence of substantial disorder. Four of the located triflimide anions were modelled as disordered over two or three locations. Substantial bond length and thermal parameter restraints were applied to facilitate stable refinement of the disordered anions and most low occupancy anions were modelled with isotropic thermal parameters. The occupancies of all located anions were allowed to freely refine which resulted in a discrepancy of ca. 2.9 anions per $Zn_6L^A{}_3L^C{}_2$ assembly.

Further reflecting the solvent loss and poor diffraction properties there is a significant amount of void volume in the lattice containing smeared electron density from disordered solvent and the remaining anions. Consequently the SQUEEZE[18] function of PLATON[19] was employed to remove the contribution of the electron density associated with these remaining anions and further highly disordered solvent, which gave a potential solvent accessible void of 3840 $Å^3$ per unit cell (a total of approximately 1180 electrons). Diffuse solvent molecules could not be assigned to acetonitrile or diethyl ether and

were therefore not included in the formula. Consequently, the molecular weight and density given above are underestimated.

CheckCIF gives one A alerts resulting from the limited resolution.

Formula $C_{272}H_{201}B_2F_{72}N_{55}O_{48}S_{24}Zn_6$, *M* 7559.15, Triclinic, space group P-1 (#2), *a* 24.8144(3), *b* 26.0438(4), *c* 29.5031(4) Å, α 103.4900(10), β 91.1400(10), γ 106.1250(10)°, *V* 17737.9(4) Å^3, D_c 1.415 g cm^{-3}, *Z* 2, crystal size 0.050 by 0.040 by 0.030 mm, colour red-brown, habit block, temperature 100(2) Kelvin, λ(Synchrotron) 0.6889 Å, μ(Synchrotron) 0.593 mm^{-1}, T(Analytical)$_{min,max}$ 0.8158102462359081, 1.0, $2\theta_{max}$ 40.30, *hkl* range -24 24, -26 26, -29 29, *N* 92867, N_{ind} 36504(R_{merge} 0.0583), N_{obs} 16928(I > 2σ(I)), N_{var} 4108, residuals* $R1(F)$ 0.0894, $wR2(F^2)$ 0.2499, GoF(all) 1.006, $\Delta\rho_{min,max}$ -0.441, 0.743 e$^-$ Å^{-3}.

*$R1 = \Sigma||F_o| - |F_c||/\Sigma|F_o|$ for $F_o > 2\sigma(F_o)$; $wR2 = (\Sigma w(F_o^2 - F_c^2)^2/\Sigma(wF_c^2)^2)^{1/2}$ all reflections

$w=1/[\sigma^2(F_o^2)+(0.1309P)^2]$ where $P=(F_o^2+2F_c^2)/3$

5 DFT calculations

5.1 Calculated energies of 1 with outward and inward fluoride addition

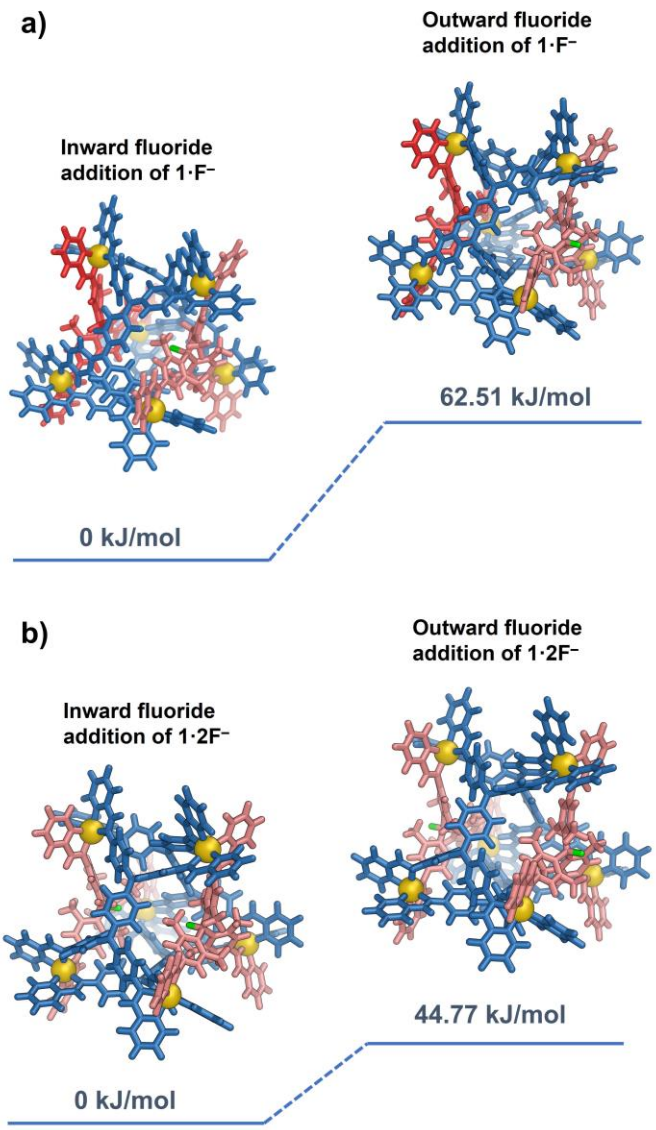

Figure S77. Energy comparisons between outward and inward fluoride addition for a) **1**·F^- and b) **1**·$2F^-$ (The D_3 symmetric structure of **1**·$2F^-$ excludes the possibility of inward fluoride addition on one side and outward fluoride addition on the other.). Inward fluoride binding was found to be energetically more favorable than outward binding.

5.2 Cavity volume calculation

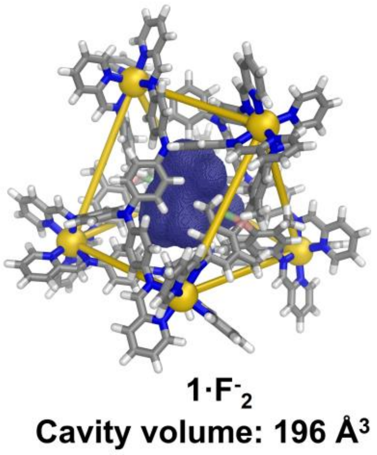

Figure S78 DFT-minimized[6] structure of **1**·2F$^-$, and its cavity volume[7] depicted in deep blue mesh.

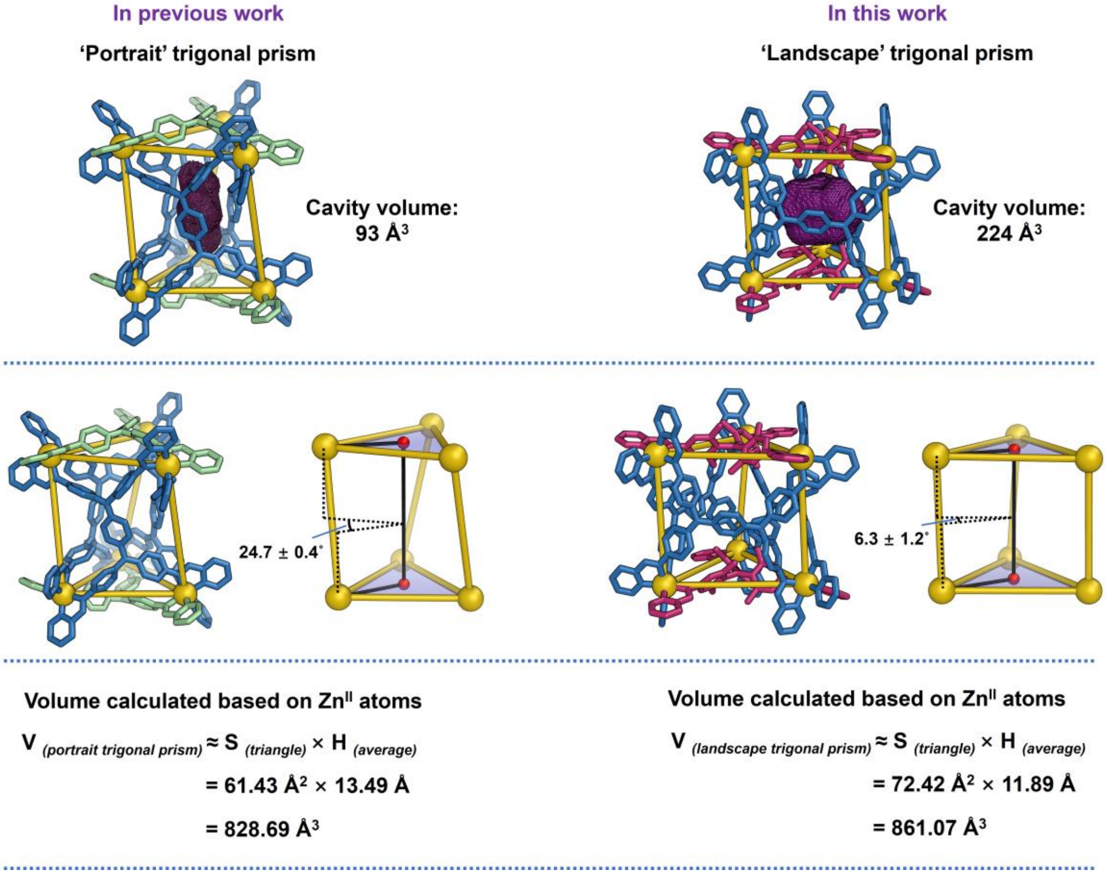

Figure S79 Illustration of the rationale behind the larger cavity volume of the 'landscape' trigonal prismatic capsule. In the 'landscape' trigonal prismatic capsule, the longer side of the rectangular ligand increases the triangular face area, thereby slightly enlarging the overall volume compared to the 'portrait' trigonal prismatic capsule. Additionally, the shorter side of the rectangular ligand reduces structural twisting in the 'landscape' configuration. Together, these factors contribute to the significantly larger internal cavity volume of the 'landscape' capsule (224 Å^3) relative to the 'portrait' one (93 Å^3).

5.3 DFT-minimized host-guest structure of G1⊂1·2F⁻

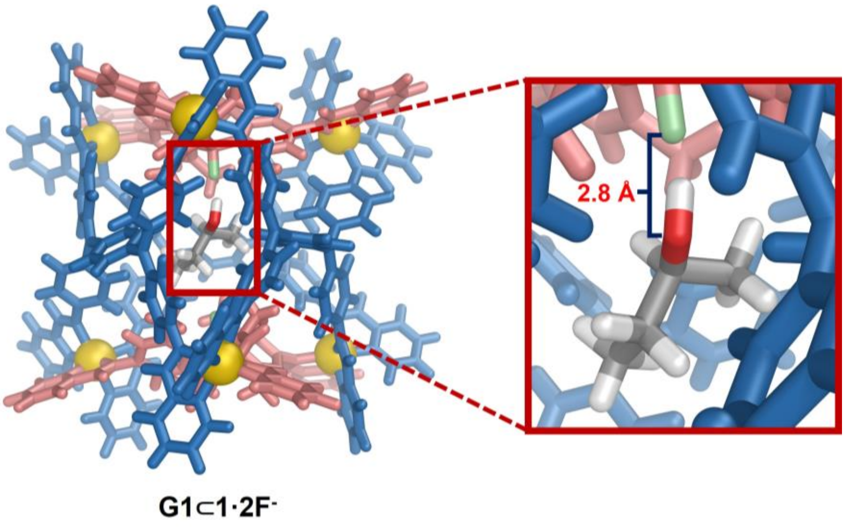

Figure S80 DFT-minimized[6] host-guest structure of **G1⊂1**·2F⁻.

5.4 Cartesian coordinates of 1·2F⁻, G1⊂1·2F⁻ and 1·F⁻

Table S1. Cartesian coordinates (in Å) for the B3LYP 6-31G(d) model of **1**·2F⁻.

Zn	6.08870	7.21271	2.53145	C	6.70786	9.38761	4.85979
N	6.44754	5.86030	0.74783	C	7.14008	7.96059	-0.22332
N	5.81105	-7.90609	5.73028	C	7.24502	9.88255	1.06033
N	3.90305	-5.85460	5.91013	C	6.96101	3.53417	1.33090
N	3.90459	8.04546	2.11424	C	5.31068	3.96893	-0.35362
N	5.81336	8.91523	3.98007	C	4.33399	-9.34127	6.98367
N	6.94708	8.58005	0.96611	C	6.48154	-10.15180	6.29219
C	6.22248	4.45169	0.58634	C	3.35925	4.82808	3.24282
C	6.84878	6.52130	-0.28057	C	3.83560	4.59899	5.60468
C	4.63334	-8.12379	6.36429	C	1.54095	7.41303	1.84540
C	6.70509	-8.90455	5.69928	C	3.02315	7.24363	-0.04303
C	3.66300	-7.02788	6.39208	C	4.33612	10.71755	4.59795
C	4.24965	4.98316	4.31470	C	6.48447	10.52437	5.64404
C	2.82827	7.57683	1.30736	C	7.63356	8.63541	-1.34207
C	3.66466	9.04907	2.88994	C	7.74651	10.62931	-0.01152
C	4.63535	9.57280	3.85264	C	6.81975	2.15204	1.12383

C	5.10915	2.59181	-0.52533	C	-2.48697	5.21465	-0.11822
C	5.27424	-10.37356	6.95085	C	-2.28652	7.59007	-0.52120
C	2.08419	4.31209	3.45346	C	6.95972	-0.61524	-3.72764
C	2.56427	4.06437	5.80914	C	7.77783	1.01800	-2.06777
C	0.49486	6.92096	1.07618	C	5.30999	-2.29179	-3.26092
C	1.96844	6.79124	-0.83013	C	4.07027	-2.46279	-1.13414
C	5.27684	11.20528	5.50786	C	6.95977	-2.92152	2.39495
C	7.94342	9.99417	-1.23531	C	7.77753	-2.30007	0.15058
C	5.85866	1.62655	0.21200	C	5.30985	-1.67960	3.61382
C	7.77855	1.27976	1.91499	C	4.06952	0.24756	2.69900
C	4.06965	2.21310	-1.56452	C	-1.00965	0.19991	6.24632
C	1.66989	3.92933	4.73649	C	0.77196	-0.26470	4.69176
C	0.67917	6.59950	-0.28488	C	-2.92299	4.90911	3.84722
B	5.46817	-0.00057	-0.00035	C	-1.63601	6.58896	4.99559
N	0.33425	3.43750	4.93818	C	0.93717	3.32635	-3.19269
N	-0.39222	6.10538	-1.05571	C	-0.84838	4.43798	-4.37047
C	5.85830	-0.63031	-1.51569	C	-3.75199	5.40905	0.43051
C	5.85827	-0.99808	1.30262	C	-3.56333	7.78819	0.00937
C	0.13326	2.04010	5.14009	C	6.22118	-1.71899	-4.14952
C	-0.73302	4.33794	4.73842	C	6.22126	-2.73535	3.56183
C	-0.19824	5.20848	-2.15305	C	-0.19851	-0.73966	5.58668
C	-1.73259	6.30408	-0.57798	C	-2.79834	6.24142	4.27276
C	6.81897	-0.10339	-2.42715	C	0.13226	3.43157	-4.33707
C	5.10895	-1.75184	-1.98241	C	-4.29566	6.69879	0.50103
C	6.81889	-2.05089	1.30168	N	6.44571	-2.28335	-5.45027
C	5.10868	-0.84206	2.50718	N	6.44616	-3.57967	4.70087
C	-0.84762	1.56620	6.02830	N	-0.39295	-2.13838	5.81472
C	0.93780	1.10135	4.47675	N	5.52748	5.54683	4.04201
C	-1.91725	3.97333	4.07064	N	0.33269	2.55796	-5.44634
C	-0.62641	5.66417	5.21238	N	-5.57780	6.87133	1.11294
C	0.77197	4.19572	-2.11718	Zn	6.08665	-1.41424	-7.51305
C	-1.00978	5.31007	-3.29626	C	6.84678	-3.50452	-5.50885

Zn	6.08721	-5.80055	4.97990
C	6.84721	-3.01974	5.78769
C	0.67822	-3.05325	5.85735
C	-1.73346	-2.65113	5.74804
Zn	-6.10453	6.83605	3.35562
C	6.55155	5.18683	4.73634
C	1.66817	2.13725	-5.77186
C	-0.73488	1.93488	-6.12594
C	-6.55334	7.31974	0.40742
N	5.52563	0.72686	-6.82550
N	8.01882	-0.28208	-7.53313
N	3.90250	-2.19136	-8.02555
N	5.81129	-1.01049	-9.71169
N	6.94498	-3.45360	-7.91510
C	7.13787	-4.17430	-6.78412
N	5.52595	-6.27541	2.78198
N	8.01912	-6.38457	4.00938
N	6.94620	-5.12906	6.94677
N	-5.57906	-4.39842	5.39427
N	-8.05608	-5.58500	5.09869
C	7.13868	-3.78918	7.00534
C	0.49354	-4.39275	5.45553
C	1.96767	-2.67708	6.29563
C	-2.48773	-2.50490	4.57457
C	-2.28762	-3.34277	6.83357
N	-6.46736	4.61877	3.68923
N	-8.05457	7.20809	2.28888
N	-6.82437	6.71245	5.46509
N	8.02075	6.66392	3.52200
C	7.88882	5.74612	4.51023
C	2.08236	0.83468	-5.46196
C	2.56256	2.99864	-6.42517
C	-1.91867	1.53849	-5.47573
C	-0.62907	1.68275	-7.51162
C	-7.89992	7.49450	0.97334
C	4.24780	1.24492	-6.47379
C	6.54966	1.50821	-6.86128
C	7.88693	1.03269	-7.23252
C	9.23480	-0.74699	-7.84573
C	2.82620	-2.65575	-7.21619
C	3.66252	-2.02131	-9.28253
C	4.63326	-1.44955	-10.21747
C	6.70589	-0.48496	-10.56062
C	7.24291	-4.02296	-9.09036
C	7.63116	-5.48064	-6.80952
C	4.24811	-6.22926	2.15752
C	6.54983	-6.69716	2.12303
C	7.88708	-6.78139	2.72040
C	9.23514	-6.42319	4.56821
C	7.24465	-5.86205	8.02742
C	2.82692	-4.92127	5.90764
C	-6.55430	-4.01177	6.13584
C	-7.90099	-4.58905	6.00458
C	-9.27899	-6.09851	4.91939
C	7.63201	-3.15781	8.14925
C	1.53945	-5.30516	5.49709
C	3.02219	-3.58513	6.29387
C	-3.75288	-3.07705	4.46874
C	-3.56455	-3.90112	6.74000
C	-6.27191	3.50918	2.80458
C	-6.78556	4.37047	4.91162
C	-9.27729	7.30954	2.82366
C	-7.01126	5.43994	5.89160
C	-7.05889	7.71251	6.32488

C	9.23672	7.16722	3.27589	C	-10.23650	8.03013	0.74052
C	8.97489	5.32843	5.28464	C	10.23661	1.42308	-7.60158
C	3.35737	0.39420	-5.80356	C	1.96652	-4.11416	-5.46711
C	3.83382	2.55419	-6.78603	C	0.49290	-2.52790	-6.53229
C	-2.92465	0.87703	-6.17401	C	5.27492	-0.83252	-12.45885
C	-1.63898	1.03266	-8.20372	C	2.56256	-7.06328	0.61435
C	-8.96390	7.91262	0.17166	C	2.08292	-5.14734	2.00726
C	8.97305	1.91213	-7.25803	C	10.23662	-7.29674	2.56661
C	10.37202	0.06792	-7.89464	C	-10.23740	-4.65485	6.58557
C	3.02117	-3.65865	-6.25249	N	-3.83377	7.16328	3.96633
C	1.53890	-2.10780	-7.34316	C	-5.16390	1.41128	2.23929
C	4.33409	-1.37652	-11.58152	C	-6.86396	2.30634	0.77000
C	6.48257	-0.37422	-11.93722	C	-7.68385	6.19402	8.07821
C	7.74421	-5.32463	-9.20150	N	-3.83644	-0.14648	-8.18544
C	7.94095	-6.06728	-8.03978	C	0.67724	-3.54607	-5.57355
C	3.83386	-7.15394	1.17953	C	1.66843	-6.06672	1.03401
C	3.35795	-5.22336	2.55945	Zn	-6.10609	-6.32268	4.24249
C	8.97304	-7.24335	1.97139	C	-3.57733	8.43043	3.99303
C	10.37221	-6.87319	3.88680	C	-5.90762	1.28055	1.02686
C	7.74609	-5.30728	9.21013	C	-4.11005	0.41071	2.67937
C	-8.96464	-4.10374	6.76793	C	-7.82186	2.27490	-0.40785
C	-10.39942	-5.66373	5.63912	Zn	-6.10736	-0.51178	-7.59613
C	7.94233	-3.92981	9.27235	C	-3.58030	-0.75721	-9.29609
C	-4.29677	-3.78251	5.55062	N	-0.39399	-3.96642	-4.75994
C	-5.37833	2.48141	3.11858	N	0.33279	-5.99503	0.50731
C	-6.99683	3.41956	1.61735	N	-6.46931	-5.50225	2.15567
C	-10.39803	7.71515	2.08757	N	-3.83499	-7.01576	4.22007
C	-7.43738	5.14498	7.18812	N	-5.85194	-7.56429	6.11405
C	-7.49120	7.50144	7.63863	N	-5.85070	9.07776	3.49463
C	10.37389	6.80215	4.00618	N	-6.82587	-8.08738	3.08028
C	10.23844	5.87059	5.03310	C	-4.58490	9.46732	3.77949
C	-2.80077	0.57988	-7.54075	B	-5.51699	0.00089	0.00054

N	-5.58032	-2.47154	-6.50570
N	-6.46946	0.88600	-5.84274
N	-6.82713	1.37685	-8.54386
N	-8.05743	-1.62153	-7.38435
N	-5.85386	-1.51232	-9.60696
C	-4.58812	-1.46064	-10.08696
C	-0.19958	-4.46835	-3.43461
C	-1.73448	-3.65202	-5.17048
C	-0.73448	-6.27228	1.38692
C	0.13192	-5.47131	-0.80390
C	-6.78803	-6.43646	1.32935
C	-6.27337	-4.18136	1.63712
C	-2.79972	-6.82053	3.26835
C	-3.57858	-7.67247	5.30409
C	-4.58611	-8.00575	6.30894
C	-6.78390	-7.89675	7.01953
C	-6.78271	10.02809	3.32979
C	-7.01369	-7.81998	1.76524
C	-7.06024	-9.33212	3.51619
C	-4.23128	10.81706	3.89593
C	-5.90797	-1.52768	0.59580
C	-5.90826	0.24978	-1.62074
C	-4.29795	-2.91513	-6.05088
C	-6.55585	-3.30677	-6.54095
C	-6.27355	0.67476	-4.43955
C	-6.78729	2.06883	-6.23900
C	-7.01346	2.38263	-7.65514
C	-7.06225	1.62123	-9.83975
C	-7.90259	-2.90405	-6.97477
C	-9.28031	-1.20918	-7.73916
C	-6.78606	-2.13017	-10.34737
C	-4.23477	-2.03489	-11.31406

C	-1.01059	-5.50956	-2.95087
C	0.77068	-3.93063	-2.57570
C	-2.28844	-4.24521	-6.31289
C	-2.48900	-2.70979	-4.45617
C	-0.62766	-7.34552	2.29888
C	-1.91901	-5.51208	1.40455
C	-0.84866	-6.00384	-1.65859
C	0.93632	-4.42730	-1.28509
C	-5.37995	-3.93987	0.58980
C	-6.99761	-3.10799	2.15329
C	-1.63723	-7.62008	3.20821
C	-2.92468	-5.78638	2.32676
C	-4.23244	-8.78139	7.41966
C	-6.51367	-8.67664	8.14808
C	-6.51255	11.39541	3.44101
C	-7.44056	-8.79494	0.86149
C	-7.49317	-10.36399	2.67644
C	-5.20957	11.79809	3.72788
C	-6.86419	-1.81763	1.61285
C	-5.16499	-2.64344	0.10259
C	-5.16506	1.23459	-2.34043
C	-6.86472	-0.48581	-2.38030
C	-3.75426	-2.33198	-4.89839
C	-3.56542	-3.88485	-6.74934
C	-5.37999	1.46088	-3.70677
C	-6.99806	-0.30872	-3.76801
C	-7.43955	3.65293	-8.04797
C	-7.49454	2.86451	-10.31383
C	-8.96649	-3.80747	-6.93579
C	-10.40097	-2.04955	-7.72216
C	-6.51617	-2.71771	-11.58707
C	-5.21326	-2.67089	-12.07942

C	-5.21066	-9.12633	8.35336
C	-7.68676	-10.09046	1.32463
C	-7.82140	-0.78136	2.17477
C	-4.11140	-2.52493	-0.98428
C	-4.11120	2.11630	-1.69440
C	-7.82228	-1.49014	-1.76362
C	-7.68657	3.89909	-9.40144
C	-10.23923	-3.37365	-7.32159
H	7.63797	-8.70116	5.18515
H	2.70526	9.57018	2.84420
H	7.64100	8.84098	4.93980
H	7.08150	10.34710	2.02732
H	7.71328	3.89752	2.02574
H	4.72611	4.67558	-0.93864
H	3.38285	-9.47300	7.49029
H	7.24360	-10.92218	6.23642
H	3.67789	5.08672	2.24005
H	4.48201	4.76173	6.46206
H	1.37061	7.60309	2.90100
H	3.99752	7.39439	-0.49573
H	3.38472	11.22190	4.45954
H	7.24693	10.86129	6.33864
H	7.78251	8.10274	-2.27624
H	7.97704	11.68116	0.12172
H	5.06862	-11.32590	7.42994
H	1.39989	4.20738	2.61802
H	2.24720	3.78665	6.80973
H	-0.46919	6.76174	1.54441
H	2.14151	6.58902	-1.88111
H	5.07132	12.09607	6.09352
H	8.33465	10.54119	-2.08757
H	8.61553	1.87632	2.29335
H	7.29189	0.80391	2.77256
H	8.19915	0.47430	1.30852
H	3.82293	3.08150	-2.18601
H	4.42138	1.41276	-2.21994
H	3.15611	1.84191	-1.09187
H	-1.46524	2.27135	6.57579
H	1.68954	1.43639	3.77117
H	-2.03999	2.96046	3.70146
H	0.24435	5.95323	5.79132
H	1.39985	4.07385	-1.24068
H	-1.75480	6.09718	-3.35613
H	-2.07128	4.21347	-0.17304
H	-1.71459	8.43742	-0.88691
H	7.71160	-0.19516	-4.39018
H	8.61440	1.04759	-2.77406
H	7.29097	1.99851	-2.08404
H	8.19898	0.89543	-1.06723
H	4.72549	-3.15195	-3.57996
H	3.82341	-3.43522	-1.57536
H	4.42285	-2.63031	-0.11361
H	3.15667	-1.86809	-1.04819
H	7.71173	-3.70524	2.36217
H	8.19899	-1.37224	-0.24312
H	7.29035	-2.80376	-0.69064
H	8.61392	-2.92702	0.47763
H	4.72528	-1.52618	4.51828
H	3.82291	0.35153	3.76184
H	3.15585	0.02424	2.14126
H	4.42155	1.21526	2.33372
H	-1.75483	-0.14152	6.95788
H	1.39959	-0.96308	4.14799
H	-3.80216	4.60823	3.29198

H	-1.53292	7.57166	5.44466	H	-3.98128	-4.44338	7.58404
H	1.68876	2.54762	-3.13022	H	-6.94025	3.34360	5.25141
H	-1.46627	4.55966	-5.25469	H	-9.36490	7.06916	3.87917
H	-4.32686	4.56408	0.79599	H	-6.89888	8.71822	5.95095
H	-3.97992	8.79032	0.05735	H	9.30418	7.89445	2.47241
H	7.02749	-4.07619	-4.59508	H	8.83134	4.59804	6.07500
H	7.02769	-1.94252	5.82586	H	3.67592	-0.60359	-5.52624
H	6.47252	4.42136	5.51178	H	4.48024	3.21529	-7.35570
H	-6.42506	7.55615	-0.65268	H	-3.80338	0.54616	-5.63540
H	-0.47066	-4.71848	5.08374	H	-1.53661	0.93072	-9.27942
H	2.14102	-1.66573	6.64568	H	-8.80001	8.14452	-0.87637
H	-2.07190	-1.95735	3.73466	H	8.82955	2.96185	-7.02084
H	-1.71577	-3.44949	7.75033	H	11.33310	-0.35799	-8.16354
H	1.39803	0.16353	-4.95358	H	3.99562	-4.12592	-6.15672
H	2.24555	4.00409	-6.68485	H	1.36856	-1.28855	-8.03543
H	-2.04082	1.72492	-4.41385	H	3.38268	-1.74848	-11.94913
H	0.24124	2.04003	-8.05187	H	7.24509	0.05878	-12.57631
H	6.47063	2.56258	-6.58643	H	7.97476	-5.73490	-10.17916
H	9.30219	-1.80646	-8.07368	H	8.33200	-7.07892	-8.08766
H	2.70303	-2.32129	-9.71091	H	3.67672	-4.48456	3.28508
H	7.63902	-0.14237	-10.12719	H	8.82939	-7.56257	0.94367
H	7.07958	-3.41753	-9.97601	H	11.33332	-6.89341	4.39003
H	7.77997	-6.02361	-5.88127	H	7.97706	-5.94873	10.05421
H	6.47066	-6.98602	1.07244	H	-8.80037	-3.31219	7.49282
H	9.30272	-6.09101	5.59975	H	-11.36826	-6.11863	5.46035
H	7.08164	-6.93183	7.94601	H	8.33345	-3.46530	10.17230
H	-6.42568	-3.21208	6.87077	H	-4.81205	2.53481	4.04572
H	-9.36704	-6.89229	4.18338	H	-7.73091	4.18658	1.38559
H	7.78050	-2.08240	8.15528	H	-11.36671	7.78773	2.57122
H	1.36884	-6.31442	5.13416	H	-7.58300	4.11260	7.49057
H	3.99675	-3.26859	6.65040	H	-7.67272	8.34873	8.29161
H	-4.32776	-2.97121	3.55423	H	11.33495	7.24809	3.77197

H	11.09630	5.57164	5.62755
H	-11.08029	8.36090	0.14272
H	11.09451	2.08731	-7.63991
H	2.13965	-4.92322	-4.76649
H	-0.47110	-2.04260	-6.62819
H	5.06945	-0.77075	-13.52314
H	2.24532	-7.79071	-0.12663
H	1.39878	-4.37141	2.33449
H	11.09440	-7.66216	2.01042
H	-11.08095	-4.30245	7.17124
H	-8.02293	5.99311	9.08979
H	-2.56912	8.80006	4.18561
H	-3.85151	0.56136	3.73393
H	-4.45584	-0.61785	2.55488
H	-3.20309	0.50380	2.07655
H	-7.33720	2.57139	-1.34368
H	-8.66142	2.95654	-0.23596
H	-8.23803	1.27849	-0.57288
H	-2.57216	-0.77552	-9.71264
H	-6.94313	-6.21700	0.27027
H	-2.57043	-8.02430	5.52784
H	-7.78870	-7.53340	6.83366
H	-7.78747	9.68540	3.10793
H	-6.89958	-9.51153	4.57397
H	-3.20684	11.09080	4.12866
H	-6.42742	-4.34308	-6.21579
H	-6.94150	2.87669	-5.51967
H	-6.90273	0.79439	-10.52369
H	-9.36815	-0.17487	-8.05861
H	-7.79080	-2.15067	-9.93960
H	-3.21037	-1.97038	-11.66766
H	-1.75560	-5.95520	-3.60245
H	1.39819	-3.11041	-2.90858
H	-1.71640	-4.98511	-6.86440
H	-2.07334	-2.25692	-3.56158
H	0.24327	-7.99122	2.26006
H	-2.04202	-4.68637	0.71149
H	-1.46612	-6.83072	-1.32175
H	1.68784	-3.98351	-0.64207
H	-4.81424	-4.76980	0.17232
H	-7.73157	-3.29033	2.93365
H	-1.53393	-8.50004	3.83510
H	-3.80410	-5.15545	2.34349
H	-3.20799	-9.11980	7.54031
H	-7.31194	-8.92360	8.84022
H	-7.31084	12.11827	3.30878
H	-7.58697	-8.54027	-0.18359
H	-7.67445	-11.35328	3.08348
H	-4.96150	12.85065	3.82436
H	-4.32932	-1.59360	-4.34873
H	-3.98202	-4.34392	-7.64143
H	-4.81408	2.23721	-4.21681
H	-7.73215	-0.89318	-4.31612
H	-7.58477	4.43118	-7.30519
H	-7.67653	3.00623	-11.37403
H	-8.80243	-4.83105	-6.61273
H	-11.36977	-1.66703	-8.02652
H	-7.31463	-3.19356	-12.14682
H	-4.96538	-3.11378	-13.03919
H	-4.96252	-9.73609	9.21670
H	-8.02638	-10.86578	0.64480
H	-7.33621	-0.11936	2.89931
H	-8.66108	-1.27059	2.67938
H	-8.23744	-0.14007	1.39446

H	-4.45678	-1.90230	-1.81255	H	-8.66155	-1.68311	-2.43996
H	-3.85393	-3.51367	-1.38143	H	-8.02564	4.87560	-9.73323
H	-3.20386	-2.05044	-0.60232	H	-11.08297	-4.05680	-7.30896
H	-3.20333	1.54833	-1.47565	H	4.48007	-7.97789	0.89154
H	-3.85446	2.95521	-2.35161	H	2.70370	-7.24838	6.86667
H	-4.45595	2.52138	-0.74045	F	-4.05756	0.00098	0.00012
H	-8.23888	-1.13416	-0.81864	F	4.00779	-0.00045	-0.00028
H	-7.33722	-2.44838	-1.55139	H	7.02933	6.01556	-1.23244

Table S2. Cartesian coordinates (in Å) for the B3LYP 6-31G(d) model of **G1**$\subset$**1**$\cdot 2F^{-}$.

Zn	6.19019	-5.60195	-5.21910	C	5.17616	-3.74001	-1.34559
N	6.42543	-5.00743	-3.05080	C	4.44767	11.37494	-3.37927
N	5.78350	9.67513	-2.31063	C	6.38702	11.99945	-2.11541
N	4.01091	7.73027	-3.27047	C	4.16231	-1.59078	-5.87744
N	4.07063	-6.64069	-4.90531	C	3.17625	-3.68787	-6.51843
N	5.86314	-6.87711	-7.03702	C	2.61108	-5.22979	-3.57077
N	7.12718	-7.43146	-4.22290	C	2.46598	-7.62977	-3.28398
C	6.11573	-3.77995	-2.37532	C	4.54990	-8.70174	-7.90640
C	6.80296	-5.99593	-2.31764	C	6.47344	-7.93023	-9.11477
C	4.71800	10.03962	-3.06398	C	7.70201	-8.30063	-2.05054
C	6.58807	10.63987	-1.84673	C	8.07321	-9.63599	-4.00815
C	3.81758	8.98103	-3.52450	C	6.63616	-1.44223	-1.93611
C	4.32427	-2.95164	-6.19281	C	4.92578	-2.55622	-0.63720
C	3.06532	-6.51558	-3.89686	C	5.30048	12.37386	-2.90202
C	3.90140	-7.54955	-5.80338	C	2.90651	-0.99595	-5.87736
C	4.80820	-7.72225	-6.94346	C	1.91822	-3.10111	-6.52201
C	6.66576	-6.98029	-8.10381	C	1.49038	-5.06749	-2.75824
C	7.20572	-7.29093	-2.87894	C	1.37544	-7.45749	-2.42996
C	7.55542	-8.57938	-4.76507	C	5.40118	-8.81276	-9.00986
C	6.82501	-2.61926	-2.67845	C	8.14380	-9.49550	-2.62411

C	5.65320	-1.35752	-0.90581	C	6.76762	3.70139	-0.85979
C	7.58214	-0.30521	-2.28206	C	7.51092	2.17613	0.93026
C	3.86240	-2.67094	0.44205	C	5.21653	3.09116	-2.58117
C	1.74493	-1.73857	-6.18668	C	3.97481	0.96955	-2.65890
C	0.84987	-6.17939	-2.19557	C	0.09901	1.84816	-3.90922
B	5.25798	0.02159	-0.03206	C	-0.12561	2.53865	-6.21314
N	0.47247	-1.15273	-6.13451	C	-2.64843	-3.14186	-5.58953
N	-0.34804	-6.02033	-1.40906	C	-2.04861	-3.25653	-7.93088
C	5.60452	-0.08299	1.61498	C	0.70782	-4.45811	1.82554
C	5.66510	1.48816	-0.75616	C	-0.62103	-6.41239	2.31180
C	0.28727	0.20512	-5.67867	C	-3.98668	-5.50043	-2.02513
C	-0.70142	-1.93704	-6.39970	C	-2.96821	-6.31836	-4.04567
C	-0.20459	-5.77836	0.00061	C	5.95854	-0.24052	4.46376
C	-1.59690	-5.99200	-2.05061	C	6.09733	4.02709	-2.03713
C	6.55151	-0.96668	2.21500	C	-0.10974	2.86349	-4.85116
C	4.86161	0.73396	2.51897	C	-2.86299	-3.68441	-6.86496
C	6.59880	2.44390	-0.25443	C	0.13522	-5.32294	2.76848
C	4.97704	1.86302	-1.94957	C	-4.12117	-5.89410	-3.36819
C	0.30935	0.52790	-4.31437	N	6.23923	-0.24028	5.87329
C	0.06729	1.21747	-6.62222	N	6.39031	5.25382	-2.72645
C	-1.57039	-2.28910	-5.35824	N	-0.29398	4.23553	-4.43559
C	-0.98246	-2.39129	-7.69800	N	5.58825	-3.59973	-6.17046
C	0.54157	-4.68384	0.46052	N	0.29872	-5.10164	4.17901
C	-0.78598	-6.63910	0.94433	N	-5.37613	-5.86722	-4.03785
C	-2.75510	-5.53764	-1.38450	Zn	5.98596	-1.82926	7.47847
C	-1.73800	-6.38333	-3.40175	C	6.61835	0.88334	6.37648
C	6.68391	-1.06885	3.61012	Zn	6.09213	7.44268	-2.15692
C	7.52240	-1.82245	1.41944	C	6.80830	5.14053	-3.93980
C	5.05606	0.66558	3.90575	C	0.87547	5.04649	-4.23502
C	3.84651	1.76623	2.06291	C	-1.56607	4.73834	-4.12727

Zn	-5.98925	-4.91576	-6.03894	C	-7.76378	-6.25660	-3.99036
C	6.62339	-2.97752	-6.62539	C	4.12658	-4.16710	5.79805
C	1.56406	-4.83749	4.73712	C	6.41504	-4.50880	6.14409
C	-0.88131	-4.84711	4.95554	C	7.75562	-4.12675	6.59341
C	-6.41699	-6.30288	-3.41125	C	9.11131	-2.54295	7.60430
N	5.37892	-3.76098	6.33213	C	2.90222	-0.13702	7.59418
N	7.89344	-2.91564	7.18756	C	3.72100	-1.24672	9.46306
N	3.88600	-0.96853	8.21525	C	4.59815	-2.16434	10.19756
N	5.62802	-2.72318	9.51729	C	6.40077	-3.61047	10.15602
N	6.93691	-0.05769	8.56966	C	7.36789	0.04185	9.83456
C	7.01354	1.03907	7.78071	C	7.50799	2.26061	8.24582
N	5.41025	7.37224	0.10141	C	4.15052	7.10625	0.70269
N	7.95805	7.70350	-1.00911	C	6.42704	7.63159	0.85557
N	7.07319	7.51905	-4.20831	C	7.77965	7.82548	0.32932
N	-5.42700	6.27890	-3.14359	C	9.19164	7.86240	-1.50842
N	-7.99203	7.12710	-2.44152	C	7.48355	8.57837	-4.91867
C	7.19496	6.29083	-4.76412	C	3.02835	6.78973	-3.69943
C	1.74089	4.80301	-3.16101	C	-6.44144	5.96847	-3.88026
C	1.15730	6.11874	-5.09710	C	-7.80510	6.41336	-3.57832
C	-2.72360	3.94357	-4.28348	C	-9.22992	7.54111	-2.13897
C	-1.74201	6.04459	-3.61656	C	7.71860	6.09966	-6.04535
N	-6.28340	-2.76577	-5.33890	C	2.81492	5.65384	-2.90557
N	-7.90284	-5.65158	-5.19495	C	2.21901	6.98166	-4.83615
N	-6.85809	-3.84635	-7.83781	C	-3.98206	4.43419	-3.96033
N	8.10666	-4.75766	-5.96319	C	-3.00322	6.52490	-3.28988
C	7.96900	-3.55709	-6.57807	C	-6.04248	-2.14398	-4.06750
C	2.75471	-5.18048	4.05679	C	-6.62737	-1.99651	-6.31142
C	1.69900	-4.19948	5.98953	C	-9.12094	-5.59926	-5.75039
C	-1.73379	-3.78300	4.62686	C	-6.95968	-2.50972	-7.64660
C	-1.17939	-5.63123	6.08107	C	-7.22165	-4.34598	-9.02668

C	9.32450	-5.31402	-5.91133	C	10.31800	-3.48727	-7.11315
C	9.05315	-2.89604	-7.16282	C	-3.04764	-4.17385	6.63062
C	4.00005	-4.84015	4.56784	C	-10.11451	-6.78304	-3.91232
C	2.94981	-3.87595	6.50131	C	10.10336	-4.60184	6.86495
C	-2.81001	-3.45448	5.44941	C	1.31936	1.70495	7.63273
C	-2.25022	-5.30235	6.90737	C	1.26633	0.10380	5.82562
C	-8.84900	-6.83167	-3.32248	C	5.15568	-3.38469	12.20013
C	8.83866	-4.99409	6.41965	C	1.72031	7.14076	0.52631
C	10.24411	-3.35080	7.46284	C	2.75703	6.24893	2.51137
C	2.39381	1.02106	8.20650	C	10.12017	8.30128	0.66260
C	2.37264	-0.54891	6.36279	C	-10.14828	6.55595	-4.12692
C	4.33566	-2.46709	11.53654	N	-3.86312	-4.68817	-7.01355
C	6.20188	-3.97338	11.49423	C	-4.89393	-0.48690	-2.69764
C	7.88384	1.22423	10.37580	C	-6.66102	-1.82545	-1.73319
C	7.95027	2.35679	9.56740	C	-7.79195	-2.17589	-9.88306
C	2.97688	7.42083	0.00364	N	-4.05624	-3.70682	7.52091
C	4.00914	6.51539	1.97475	C	0.71894	1.22654	6.46103
C	8.83897	8.12827	1.19141	C	1.57577	6.52832	1.78842
C	10.30217	8.15877	-0.71203	Zn	-6.10985	7.55092	-1.34736
C	8.02439	8.47312	-6.20468	C	-3.65513	-5.64093	-7.85832
C	-8.86211	6.11229	-4.44324	C	-5.67467	-0.81305	-1.54803
C	-10.33825	7.27901	-2.95035	C	-3.80683	0.57313	-2.69602
C	8.14034	7.21019	-6.78069	C	-7.64613	-2.23119	-0.65056
C	-4.15270	5.74088	-3.46671	Zn	-6.15838	-2.70397	7.12297
C	-5.10290	-1.12150	-3.93040	C	-3.88840	-3.88986	8.78765
C	-6.80393	-2.50244	-2.95620	N	-0.45702	1.86504	5.92767
C	-10.25527	-6.15148	-5.14679	N	0.30404	6.19043	2.29669
C	-7.41680	-1.64685	-8.64557	N	-6.38115	5.85719	0.14601
C	-7.69632	-3.55201	-10.07608	N	-4.01935	8.43117	-0.59723
C	10.45812	-4.71657	-6.47169	N	-5.89820	9.55351	-2.35273

N	-5.71497	-6.76296	-7.29876
N	-7.04244	8.53901	0.45773
C	-4.59274	-6.74881	-8.05792
B	-5.30200	-0.03652	-0.09986
N	-5.51684	-0.51323	6.90921
N	-6.42084	-3.16016	4.89756
N	-7.05617	-4.76729	7.08230
N	-8.07079	-1.57712	7.25637
N	-5.96116	-2.79780	9.36307
C	-4.85104	-3.44285	9.79646
C	-0.27320	2.95693	5.01659
C	-1.71387	1.27226	6.15296
C	-0.86183	6.74957	1.67698
C	0.12389	5.10405	3.21225
C	-6.76805	6.29602	1.29212
C	-6.10378	4.45249	0.04002
C	-3.01414	7.89194	0.25514
C	-3.86517	9.62916	-1.05360
C	-4.81671	10.27062	-1.96209
C	-6.75585	10.12103	-3.21095
C	-6.56132	-7.79097	-7.43901
C	-7.14319	7.69860	1.51460
C	-7.44244	9.80783	0.61498
C	-4.30614	-7.75577	-8.98457
C	-5.67854	1.60957	-0.07426
C	-5.72847	-0.88569	1.29891
C	-4.24652	0.06337	6.62690
C	-6.54891	0.26152	6.91783
C	-6.14805	-2.38076	3.72368
C	-6.80320	-4.37625	4.71745
C	-7.16509	-5.26616	5.82807
C	-7.44090	-5.53815	8.10853
C	-7.91062	-0.23680	7.13997
C	-9.30254	-2.06042	7.46468
C	-6.83506	-2.35833	10.27778
C	-4.60409	-3.67301	11.15352
C	-0.98058	4.16097	5.16540
C	0.64033	2.85039	3.95770
C	-1.89803	0.33478	7.19424
C	-2.82823	1.56500	5.33934
C	-1.12308	8.12637	1.76680
C	-1.73894	5.94037	0.94064
C	-0.78644	5.21764	4.27554
C	0.83111	3.90228	3.06561
C	-5.16808	3.83592	0.87084
C	-6.82314	3.65994	-0.85271
C	-2.18845	8.69059	1.07236
C	-2.80603	6.50388	0.24345
C	-4.58379	11.57427	-2.41200
C	-6.59475	11.41976	-3.70810
C	-6.34746	-8.83916	-8.34257
C	-7.63795	8.11550	2.75256
C	-7.95526	10.30264	1.81886
C	-5.20222	-8.81778	-9.13481
C	-6.65222	2.26534	-0.88457
C	-4.93035	2.45592	0.79806
C	-5.02937	-2.10068	1.57492
C	-6.66926	-0.47778	2.28952
C	-4.06717	0.98427	5.58019
C	-3.13138	-0.26939	7.41027

C	-5.25470	-2.83093	2.75017	H	7.75287	-8.14644	-0.97717
C	-6.83870	-1.19283	3.48858	H	8.41359	-10.54046	-4.50158
C	-7.65409	-6.55084	5.57869	H	5.11801	13.41762	-3.13876
C	-7.94626	-6.83193	7.94343	H	2.82194	0.04910	-5.60622
C	-8.98786	0.64853	7.24050	H	1.06211	-3.71213	-6.77694
C	-10.43086	-1.24137	7.57804	H	1.10199	-4.07005	-2.57677
C	-6.66187	-2.54790	11.65409	H	0.90959	-8.32135	-1.96568
C	-5.52824	-3.22292	12.10015	H	5.22728	-9.56929	-9.76896
C	-5.49128	12.16298	-3.29630	H	8.54121	-10.29382	-2.00493
C	-8.05133	9.44101	2.90905	H	8.43193	-0.67924	-2.86265
C	-7.60052	1.52296	-1.80979	H	7.09373	0.47771	-2.87100
C	-3.84758	1.94292	1.72795	H	7.98459	0.18191	-1.39056
C	-4.03839	-2.72504	0.61115	H	3.73807	-3.71972	0.73635
C	-7.58947	0.71895	2.11921	H	4.11314	-2.09386	1.33301
C	-8.05180	-7.34899	6.65423	H	2.89399	-2.30740	0.08459
C	-10.26855	0.13842	7.46823	H	0.49468	-0.23179	-3.55924
H	7.43028	10.31851	-1.24339	H	0.05686	0.96947	-7.67920
H	3.02728	-8.20604	-5.78270	H	-1.38678	-1.91996	-4.35445
H	7.49724	-6.28478	-8.15157	H	-0.35103	-2.07849	-8.52405
H	7.48892	-8.65873	-5.84528	H	0.98146	-3.98690	-0.24381
H	7.59896	-2.65476	-3.44079	H	-1.35301	-7.50148	0.60703
H	4.61310	-4.63899	-1.10575	H	-2.68501	-5.18438	-0.36396
H	3.58717	11.62656	-3.99184	H	-0.87890	-6.74621	-3.95270
H	7.07501	12.73754	-1.71621	H	7.43125	-1.73755	4.02981
H	5.02152	-0.99880	-5.57637	H	8.35618	-2.14052	2.05410
H	3.27419	-4.73397	-6.78708	H	7.04831	-2.72263	1.01555
H	3.09016	-4.36322	-4.00852	H	7.94620	-1.28323	0.56940
H	2.84901	-8.62948	-3.46827	H	4.48364	1.31731	4.56214
H	3.69900	-9.36671	-7.79302	H	3.68802	2.51261	2.85015
H	7.15721	-7.97133	-9.95636	H	4.16653	2.28642	1.15852

H	2.88285	1.30258	1.83383
H	7.49800	4.39734	-0.45504
H	7.93351	1.16908	0.90448
H	6.98781	2.27247	1.88704
H	8.34725	2.88294	0.93445
H	4.68853	3.33216	-3.50076
H	3.88567	1.26744	-3.71016
H	2.97769	1.04364	-2.21500
H	4.26087	-0.08304	-2.62677
H	0.09349	2.08457	-2.85081
H	-0.28782	3.31888	-6.95060
H	-3.28433	-3.43841	-4.76343
H	-2.25322	-3.58208	-8.94654
H	1.28408	-3.60017	2.15646
H	-1.05707	-7.10157	3.02854
H	-4.84401	-5.10001	-1.49238
H	-3.04524	-6.64450	-5.07724
H	6.71846	1.76535	5.73971
H	6.94190	4.15253	-4.38648
H	6.52853	-1.99476	-7.09267
H	-6.32614	-6.75342	-2.42022
H	1.56022	3.96254	-2.49980
H	0.52880	6.28501	-5.96663
H	-2.63403	2.92484	-4.63987
H	-0.88598	6.69145	-3.47160
H	2.70328	-5.69389	3.10450
H	0.81977	-3.93533	6.56308
H	-1.53309	-3.18658	3.74282
H	-0.56214	-6.49395	6.31253
H	6.32756	-5.48024	5.65295
H	9.18200	-1.56626	8.07179
H	2.87006	-0.84584	10.02071
H	7.21424	-4.04376	9.58347
H	7.30663	-0.85717	10.43892
H	7.55771	3.11557	7.57871
H	6.31849	7.73013	1.93746
H	9.29294	7.75553	-2.58361
H	7.38385	9.54815	-4.44266
H	-6.31623	5.36683	-4.78303
H	-9.33736	8.10597	-1.21794
H	7.80459	5.09719	-6.45290
H	3.45001	5.46882	-2.04732
H	2.41974	7.79368	-5.52863
H	-4.83748	3.77106	-4.04683
H	-3.10388	7.53836	-2.91957
H	-6.74030	-0.92086	-6.15517
H	-9.19092	-5.10092	-6.71249
H	-7.13620	-5.42113	-9.14581
H	9.39506	-6.27222	-5.40630
H	8.90469	-1.94131	-7.65791
H	4.88232	-5.06868	3.97771
H	3.01676	-3.38974	7.46782
H	-3.42913	-2.59772	5.20618
H	-2.47392	-5.94138	7.75625
H	-8.70077	-7.31871	-2.36363
H	8.68965	-5.96264	5.95224
H	11.20691	-3.00190	7.82122
H	2.83211	1.38953	9.12971
H	2.78248	-1.41855	5.86461
H	3.50535	-1.99215	12.05045

H	6.86132	-4.69666	11.96278	H	-2.89290	0.20532	-2.22172
H	8.22670	1.24475	11.40509	H	-7.18940	-2.88271	0.10194
H	8.34610	3.29130	9.95295	H	-8.49526	-2.76824	-1.08641
H	4.88521	6.22804	2.54760	H	-8.04564	-1.36871	-0.11187
H	8.65913	8.23335	2.25696	H	-2.98545	-4.36778	9.17442
H	11.27931	8.28046	-1.16757	H	-6.88684	5.61347	2.13726
H	8.34781	9.36515	-6.73105	H	-2.97928	10.21923	-0.80885
H	-8.67633	5.54901	-5.35253	H	-7.60832	9.52058	-3.50914
H	-11.31966	7.64094	-2.66228	H	-7.44697	-7.77594	-6.81253
H	8.55709	7.09013	-7.77594	H	-7.35598	10.45308	-0.25325
H	-4.50110	-0.83674	-4.79038	H	-3.40031	-7.70569	-9.58120
H	-7.57825	-3.25748	-3.06421	H	-6.43892	1.34005	6.78302
H	-11.21886	-6.08688	-5.64125	H	-6.92672	-4.77586	3.70796
H	-7.48877	-0.58124	-8.45122	H	-7.34748	-5.10558	9.09936
H	-7.98493	-4.01150	-11.01571	H	-9.38964	-3.13956	7.54726
H	11.42101	-5.21220	-6.40580	H	-7.71045	-1.84137	9.89946
H	11.17398	-2.99885	-7.56862	H	-3.70728	-4.20181	11.46186
H	-10.97117	-7.23171	-3.41886	H	-1.67173	4.27946	5.99419
H	10.95826	-5.26105	6.74938	H	1.20095	1.93383	3.81383
H	0.92309	2.59406	8.11362	H	-1.07159	0.08545	7.84903
H	0.81150	-0.26760	4.91232	H	-2.72063	2.23777	4.49837
H	4.97802	-3.63348	13.24194	H	-0.48470	8.75332	2.38168
H	0.84430	7.38276	-0.06269	H	-1.56448	4.87045	0.88417
H	2.69300	5.79840	3.49473	H	-1.32652	6.14768	4.42345
H	10.95653	8.54449	1.31080	H	1.52944	3.78042	2.24499
H	-10.98344	6.34335	-4.78724	H	-4.59605	4.44407	1.56797
H	-8.15763	-1.52869	-10.67428	H	-7.58921	4.11979	-1.47117
H	-2.73260	-5.68696	-8.44194	H	-2.38430	9.75206	1.18903
H	-3.56374	0.86349	-3.72504	H	-3.44330	5.86929	-0.36197
H	-4.10333	1.46827	-2.14543	H	-3.70978	12.12007	-2.06971

H	-7.32577	11.82999	-4.39708	H	-8.02770	0.76044	1.11919
H	-7.07040	-9.64482	-8.41817	H	-7.06821	1.66968	2.27011
H	-7.70906	7.40988	3.57446	H	-8.41576	0.67106	2.83656
H	-8.27266	11.33792	1.88809	H	-8.44196	-8.34831	6.48761
H	-5.00790	-9.60853	-9.85293	H	-11.12018	0.80534	7.56092
H	-4.89205	1.20259	4.90832	H	3.05166	7.89687	-0.96564
H	-3.24567	-0.96602	8.23396	H	2.92914	9.31809	-4.06329
H	-4.70957	-3.75691	2.91969	F	-3.83968	-0.06363	-0.06638
H	-7.58540	-0.86298	4.20570	F	3.76921	0.03798	-0.07461
H	-7.73245	-6.91314	4.55837	H	6.89071	-5.87958	-1.23480
H	-8.25072	-7.40970	8.80991	C	0.51889	1.36732	0.08757
H	-8.82251	1.71797	7.15369	C	0.69840	-0.14221	0.27155
H	-11.40649	-1.68290	7.75240	C	-0.61000	-0.85178	0.62872
H	-7.40685	-2.17584	12.34977	C	-1.01874	-0.64450	2.09179
H	-5.36489	-3.39653	13.15935	O	1.23161	-0.72645	-0.92708
H	-5.33802	13.17664	-3.65371	H	-0.20178	1.55847	-0.71636
H	-8.44647	9.78949	3.85829	H	0.14307	1.84725	0.99833
H	-7.09981	1.16506	-2.71487	H	1.47586	1.83357	-0.17183
H	-8.42517	2.17470	-2.11720	H	1.41948	-0.31655	1.08421
H	-8.04049	0.64546	-1.32975	H	-1.42048	-0.50936	-0.02516
H	-4.14708	1.03073	2.24876	H	-0.47500	-1.92260	0.43694
H	-3.60202	2.70825	2.47408	H	-0.27301	-1.07790	2.77332
H	-2.93805	1.69515	1.17568	H	-1.12146	0.41651	2.34446
H	-3.10510	-2.16316	0.56471	H	-1.98521	-1.11394	2.30035
H	-3.81264	-3.75153	0.92195	H	2.18597	-0.51740	-0.90597
H	-4.42739	-2.75040	-0.40951				

Table S3. Cartesian coordinates (in Å) for the B3LYP 6-31G(d) model of **1**·F^{-}.

Zn	6.03424	-3.68681	-6.83670	N	3.88877	8.37359	-0.51096
N	6.51417	-3.76210	-4.52170	N	3.88672	-4.63086	-6.99721
N	5.73971	9.85157	0.99678	N	5.73826	-4.06471	-9.03054

N	6.94731	-5.70189	-6.48165
C	6.39637	-2.80177	-3.45597
C	7.04075	-4.89399	-4.21158
C	4.56974	10.41776	0.60953
C	6.60453	10.60562	1.69092
C	3.63073	9.58522	-0.14259
C	4.23569	-0.81492	-6.66367
C	2.82541	-4.80753	-6.06072
C	3.62844	-4.91675	-8.23086
C	4.56766	-4.68174	-9.32774
C	6.60344	-3.84096	-10.03039
C	7.28239	-5.95893	-5.19414
C	7.18358	-6.64999	-7.39775
C	7.26801	-1.71354	-3.39845
C	5.44594	-2.96161	-2.44567
C	4.25113	11.74765	0.90070
C	6.35922	11.94220	2.02571
C	3.32142	-1.46394	-5.82151
C	3.84345	0.39003	-7.28208
C	1.53012	-4.34717	-6.35077
C	3.03827	-5.42703	-4.81832
C	4.24872	-5.09335	-10.62534
C	6.35787	-4.21823	-11.35556
C	7.86468	-7.16525	-4.80006
C	7.76347	-7.88646	-7.08514
C	7.23055	-0.81017	-2.32672
C	5.39009	-2.07559	-1.36011
C	5.16212	12.52686	1.61892
C	2.05099	-0.93846	-5.60821
C	2.57986	0.92783	-7.04836

C	0.49319	-4.48402	-5.43800
C	1.99148	-5.60772	-3.91739
C	5.16007	-4.86129	-11.65903
C	8.11154	-8.14933	-5.76327
C	6.29379	-0.97984	-1.26433
C	8.25838	0.30606	-2.34306
C	4.32212	-2.34269	-0.31598
C	1.66073	0.26131	-6.22011
C	0.69168	-5.12547	-4.19662
B	6.28511	-0.00143	0.00092
N	0.33487	0.76649	-6.02348
N	-0.37639	-5.27025	-3.29056
C	6.29377	-0.60776	1.48092
C	6.29472	1.58348	-0.21388
C	0.14484	1.93992	-5.23906
C	-0.74711	-0.03439	-6.47027
C	-0.18308	-5.35035	-1.87627
C	-1.72110	-5.10851	-3.77938
C	7.22929	-1.61396	1.86495
C	5.39129	-0.14123	2.47809
C	7.23219	2.41821	0.46392
C	5.39108	2.21475	-1.11464
C	-0.84415	2.88439	-5.56486
C	0.95968	2.19340	-4.12400
C	-1.88458	-0.27526	-5.68019
C	-0.69182	-0.62615	-7.74889
C	0.79331	-4.58630	-1.21869
C	-1.00417	-6.18465	-1.09768
C	-2.48167	-3.99531	-3.39364
C	-2.26915	-6.03020	-4.68119

C	7.26666	-2.09017	3.18323
C	8.25568	-2.18804	0.90601
C	5.44702	-0.63815	3.78826
C	4.32471	0.89808	2.18763
C	7.27024	3.79802	0.21746
C	8.26015	1.87363	1.43829
C	5.44747	3.59788	-1.33912
C	4.32255	1.44455	-1.86773
C	-1.00211	4.04226	-4.80697
C	0.79491	3.34767	-3.36174
C	-2.89812	-1.11686	-6.13410
C	-1.70689	-1.45845	-8.19941
C	0.95769	-4.66901	0.16214
C	-0.84657	-6.26181	0.28407
C	-3.74834	-3.78052	-3.93189
C	-3.54501	-5.82672	-5.21304
C	6.39614	-1.59442	4.15472
C	6.39849	4.39240	-0.69583
C	-0.18134	4.29924	-3.69488
C	-2.81894	-1.75431	-7.38210
C	0.14238	-5.50747	0.93923
C	-4.28493	-4.69201	-4.85175
N	6.51359	-2.03737	5.51921
N	6.51666	5.79557	-0.99444
N	-0.37461	5.48402	-2.91841
N	5.50180	-1.43249	-6.86305
N	0.33196	-5.59976	2.34773
N	-5.56742	-4.41752	-5.42460
Zn	6.03161	-4.07946	6.61177
C	7.04027	-1.20311	6.34461
Zn	6.03592	7.76250	0.22795
C	7.04380	6.09297	-2.12947
C	0.69356	6.19606	-2.33987
C	-1.71933	5.82710	-2.53454
Zn	-6.13091	-2.88052	-7.06272
C	6.52673	-0.72549	-7.20269
C	1.65774	-5.51819	2.88392
C	-0.75021	-5.58608	3.26447
C	-6.53764	-5.22999	-5.19867
N	5.49849	-5.23004	4.67332
N	7.97018	-4.96839	5.93165
N	3.88406	-3.74509	7.50940
N	5.73459	-5.78995	8.03630
N	6.94515	-2.76482	8.17953
C	7.28116	-1.52155	7.75830
N	5.50167	6.65896	2.19344
N	7.97412	7.61677	1.33845
N	6.95074	8.46291	-1.69401
N	-5.56583	6.90822	-1.11532
N	-8.05561	7.59347	-0.12375
C	7.28597	7.47634	-2.56026
C	0.49506	6.95052	-1.16376
C	1.99349	6.19487	-2.89683
C	-2.48069	4.93680	-1.76371
C	-2.26665	7.06919	-2.88210
N	-6.47167	-1.01749	-5.81753
N	-8.05706	-3.89942	-6.51330
N	-6.89953	-1.38396	-8.53034
N	7.97335	-2.65360	-7.26518
C	7.84536	-1.31614	-7.44742

C	2.04890	-4.38791	3.61580	C	-6.82984	-0.01999	-6.54854
C	2.57581	-6.56992	2.72251	C	-9.28448	-3.63175	-6.97579
C	-1.88771	-4.78155	3.07764	C	-7.09469	-0.15826	-7.98576
C	-0.69505	-6.39714	4.41654	C	-7.17188	-1.55145	-9.83138
C	-7.88727	-4.99482	-5.73270	C	9.17390	-3.21228	-7.46868
C	4.23237	-5.36555	4.03865	C	8.91822	-0.51589	-7.85291
C	6.52288	-5.87902	4.23159	C	3.31917	-4.31056	4.17801
C	7.84158	-5.79597	4.86536	C	3.83922	-6.50406	3.30556
C	9.17085	-4.86540	6.51699	C	-2.90135	-4.75359	4.03334
C	2.82304	-2.84555	7.19367	C	-1.71028	-6.37097	5.36241
C	3.62529	-4.67024	8.37397	C	-8.93925	-5.85808	-5.42013
C	4.56404	-5.73796	8.71929	C	8.91391	-6.54848	4.37578
C	6.59932	-6.76800	8.34284	C	10.29256	-5.58586	6.08918
C	7.18066	-3.08402	9.45882	C	3.03612	-1.45988	7.10896
C	7.86364	-0.57730	8.60604	C	1.52779	-3.32679	6.93958
C	4.23519	6.17774	2.62785	C	4.24471	-6.65533	9.72496
C	6.52594	6.59988	2.97641	C	6.35333	-7.72645	9.33258
C	7.84498	7.10673	2.58808	C	7.76069	-2.19524	10.37342
C	9.17504	8.07199	0.95713	C	8.10972	-0.91932	9.94006
C	7.18758	9.73025	-2.05689	C	3.84178	6.11202	3.98030
C	2.82746	7.65094	-1.13222	C	3.32188	5.77184	1.64427
C	-6.53538	7.11958	-1.93250	C	8.91703	7.05831	3.48486
C	-7.88507	7.46527	-1.46260	C	10.29650	8.06108	1.79532
C	-9.28316	7.86069	0.33862	C	7.76817	10.07763	-3.28371
C	7.86894	7.73808	-3.80171	C	-8.93642	7.62697	-2.36711
C	1.53207	7.67239	-0.58876	C	-10.39224	8.03086	-0.50080
C	3.04036	6.88450	-2.28975	C	8.11636	9.06423	-4.17218
C	-3.74737	5.29620	-1.30912	C	-4.28320	6.54884	-1.63877
C	-3.54251	7.42870	-2.44051	C	-5.34932	0.19209	-3.98322
C	-6.25236	-0.78053	-4.42037	C	-6.96950	-1.49816	-3.46479

C	-10.39416	-4.44285	-6.70312	N	0.33375	4.83266	3.67515
C	-7.56674	0.92217	-8.73350	N	-6.47179	5.54884	2.02525
C	-7.65164	-0.52148	-10.64835	N	-3.85220	8.05345	1.58405
C	10.29609	-2.48158	-7.87745	N	-5.84698	9.73675	0.50743
C	10.16350	-1.10924	-8.07987	N	-5.84882	-4.42603	-8.68516
C	-2.82236	-5.51534	5.20960	N	-6.89963	8.08154	3.06393
C	-10.21667	-5.58020	-5.91920	C	-4.59518	-4.47460	-9.19646
C	10.15934	-6.44843	5.00286	B	-5.46718	0.00163	-0.00060
C	1.98952	-0.58921	6.81459	N	-5.56973	-2.48783	6.53726
C	0.49106	-2.46777	6.60133	N	-6.47467	-4.52702	3.78884
C	5.15560	-7.66685	10.04125	N	-6.90437	-6.69320	5.46202
C	2.57802	5.64071	4.32852	N	-8.05997	-3.68857	6.63316
C	2.05126	5.32439	1.99204	N	-5.85264	-5.30756	8.17463
C	10.16272	7.55113	3.08524	C	-4.59911	-5.72637	8.47237
C	-10.21396	7.92084	-1.87764	C	-0.18417	1.04995	5.57042
N	-3.85276	-2.65340	-7.76591	C	-1.72294	-0.71859	6.31259
C	-5.12239	0.41077	-2.61702	C	-0.74788	5.62047	3.20489
C	-6.82237	-1.22713	-2.09337	C	0.14321	3.56673	4.29917
C	-7.85296	0.73813	-10.08952	C	-6.82967	5.68331	3.25468
N	-3.85631	-5.39790	6.18001	C	-6.25249	4.22036	1.53201
C	0.68975	-1.07202	6.53626	C	-2.81890	7.27100	2.17108
C	1.65986	5.25551	3.33674	C	-3.59659	9.27748	1.25819
Zn	-6.13019	7.55879	1.03444	C	-4.59337	10.20339	0.72212
C	-3.59770	-3.54811	-8.66269	C	-6.76782	10.59356	0.04223
C	-5.86046	-0.28943	-1.61483	C	-6.77035	-5.25653	-9.19457
C	-4.06431	1.45102	-2.29309	C	-7.09452	6.99718	3.85335
C	-7.77590	-1.98093	-1.18319	C	-7.17271	9.29203	3.56900
Zn	-6.13438	-4.67459	6.02499	C	-4.24341	-5.35922	-10.22239
C	-3.60154	-5.72749	7.40321	C	-5.86030	1.54516	0.55453
N	-0.37809	-0.21479	6.20823	C	-5.86091	-1.25079	1.05835

C	-4.28698	-1.85502	6.48842	C	-5.12344	-2.46916	0.95284
C	-6.53955	-1.88562	7.12826	C	-6.82306	-1.19619	2.10944
C	-6.25463	-3.43539	2.88542	C	-3.75029	-1.51467	5.23902
C	-6.83291	-5.65862	3.29007	C	-3.54707	-1.60026	7.65169
C	-7.09861	-6.83419	4.12808	C	-5.35134	-3.54321	1.82477
C	-7.17801	-7.73607	6.25730	C	-6.97107	-2.24862	3.02942
C	-7.88941	-2.46512	7.19188	C	-7.57083	-8.02169	3.56581
C	-9.28762	-4.22238	6.63290	C	-7.65810	-8.95826	5.77335
C	-6.77420	-5.33333	9.14856	C	-8.94085	-1.76253	7.78386
C	-4.24748	-6.17271	9.75143	C	-10.39678	-3.58040	7.19968
C	-1.00470	2.14182	5.90356	C	-6.50551	-5.77486	10.44871
C	0.79253	1.23687	4.58014	C	-5.21599	-6.20252	10.75698
C	-2.27110	-1.03821	7.56180	C	-5.20868	12.42032	-0.00815
C	-2.48352	-0.94138	5.15572	C	-7.85349	8.37107	5.68112
C	-0.69236	7.02360	3.33233	C	-7.77507	2.01732	-1.12687
C	-1.88521	5.05718	2.60052	C	-4.06463	1.26231	2.40157
C	-0.84615	3.37701	5.27971	C	-4.06488	-2.70888	-0.10949
C	0.95787	2.47403	3.96157	C	-7.77587	-0.03050	2.30737
C	-5.34968	3.35540	2.15602	C	-7.85833	-9.10392	4.40290
C	-6.96917	3.75172	0.43241	C	-10.21851	-2.33313	7.79306
C	-1.70704	7.83034	2.83671	H	7.53177	10.12901	1.98870
C	-2.89834	5.87152	2.09849	H	2.67378	-5.37135	-8.50529
C	-4.24092	11.53410	0.46944	H	7.53121	-3.34601	-9.76607
C	-6.49829	11.94016	-0.22527	H	6.90845	-6.41459	-8.42031
C	-6.50153	-6.16182	-10.22691	H	8.01879	-1.59345	-4.17385
C	-7.56670	7.10469	5.16285	H	4.74310	-3.78855	-2.50318
C	-7.65271	9.48471	4.86934	H	3.30862	12.16730	0.56255
C	-5.21189	-6.21510	-10.75112	H	7.09802	12.50348	2.58854
C	-6.82195	2.42852	-0.01854	H	3.61399	-2.37881	-5.32097
C	-5.12256	2.06292	1.66234	H	4.49606	0.88751	-7.99307

H	1.34531	-3.81136	-7.27719	H	8.79292	-1.41136	0.35274
H	4.01813	-5.83031	-4.58178	H	4.74500	-0.27336	4.53340
H	3.30569	-5.59488	-10.82011	H	4.75923	1.87909	1.97260
H	7.09701	-4.01183	-12.12283	H	3.70623	0.62618	1.32447
H	8.12920	-7.32892	-3.75985	H	3.65656	1.01128	3.04689
H	7.93912	-8.61526	-7.86989	H	8.02150	4.40910	0.70890
H	4.94173	13.56416	1.85225	H	8.79598	1.00595	1.04078
H	1.34938	-1.46495	-4.96960	H	7.79669	1.55825	2.37903
H	2.28201	1.84490	-7.54743	H	9.01124	2.63287	1.67387
H	-0.47827	-4.07316	-5.68504	H	4.74456	4.06151	-2.02621
H	2.17738	-6.14340	-2.99333	H	4.75533	0.76675	-2.60984
H	4.93944	-5.17699	-12.67424	H	3.65468	2.13264	-2.39505
H	8.56938	-9.09404	-5.48627	H	3.70400	0.83426	-1.19956
H	9.00965	0.12982	-3.11805	H	-1.75462	4.76882	-5.09626
H	7.79481	1.27820	-2.54138	H	1.42137	3.50238	-2.48889
H	8.79398	0.39668	-1.39286	H	-3.74524	-1.31205	-5.48905
H	4.75532	-2.64581	0.64207	H	-1.64967	-1.83193	-9.21690
H	3.70324	-1.45906	-0.12180	H	1.71296	-4.05842	0.64658
H	3.65451	-3.14386	-0.64760	H	-1.47555	-6.93666	0.85610
H	-1.47287	2.72650	-6.43551	H	-4.33040	-2.91375	-3.63490
H	1.71488	1.46851	-3.83744	H	-3.95577	-6.54184	-5.92005
H	-1.96925	0.18002	-4.69858	H	7.36236	-0.21306	6.00946
H	0.14080	-0.39781	-8.40633	H	7.36561	5.30752	-2.81923
H	1.42012	-3.90805	-1.78905	H	6.46143	0.35947	-7.30732
H	-1.75673	-6.79839	-1.58227	H	-6.40130	-6.11408	-4.56934
H	-2.07246	-3.28571	-2.68135	H	-0.47647	6.95930	-0.68460
H	-1.69439	-6.90348	-4.97446	H	2.17943	5.66226	-3.82266
H	8.01646	-2.82280	3.46675	H	-2.07212	3.96490	-1.50521
H	9.00572	-2.77305	1.44571	H	-1.69128	7.75954	-3.49148
H	7.79046	-2.84440	0.16304	H	1.34806	-3.57074	3.75116

H	2.27725	-7.46101	2.17879	H	11.23931	-5.47386	6.60771
H	-1.97230	-4.15932	2.19239	H	4.01594	-1.05345	7.34023
H	0.13762	-7.08052	4.54788	H	1.34288	-4.39699	6.93874
H	6.45709	-6.51293	3.34492	H	3.30174	-6.57255	10.25668
H	9.23800	-4.19254	7.36628	H	7.09212	-8.49439	9.53777
H	2.67053	-4.68013	8.90472	H	7.93570	-2.51037	11.39709
H	7.52706	-6.78728	7.78200	H	8.56767	-0.20722	10.61978
H	6.90466	-4.08701	9.76633	H	3.61533	5.79491	0.60191
H	8.12888	0.40517	8.22762	H	8.77482	6.65536	4.48308
H	6.45974	6.14858	3.96853	H	11.24347	8.45401	1.43950
H	9.24264	8.47142	-0.05006	H	7.94426	11.12160	-3.52238
H	6.91231	10.49817	-1.34186	H	-8.75975	7.52880	-3.43400
H	-6.39844	7.01670	-3.01274	H	-11.36511	8.25352	-0.07452
H	-9.38505	7.95350	1.41580	H	8.57471	9.29660	-5.12861
H	8.13353	6.91901	-4.46347	H	-4.79075	0.76657	-4.71873
H	1.34723	8.20700	0.33836	H	-7.71064	-2.22230	-3.79235
H	4.02034	6.88095	-2.75702	H	-11.36687	-4.18433	-7.10900
H	-4.33013	4.60590	-0.70734	H	-7.71850	1.88681	-8.25918
H	-3.95265	8.39876	-2.70651	H	-7.86324	-0.71455	-11.69497
H	-6.99228	0.96578	-6.10611	H	11.24271	-2.98684	-8.03984
H	-9.38579	-2.74533	-7.59492	H	11.00845	-0.51284	-8.41059
H	-7.00545	-2.54395	-10.23645	H	-11.05125	-6.23908	-5.69956
H	9.24056	-4.28440	-7.31165	H	11.00389	-7.03395	4.65227
H	8.77693	0.55029	-8.00236	H	2.17555	0.47887	6.81642
H	3.61244	-3.41929	4.71911	H	-0.48036	-2.88700	6.36864
H	4.49097	-7.36929	3.23154	H	4.93464	-8.38775	10.82258
H	-3.74838	-4.09732	3.87960	H	2.27928	5.61526	5.37204
H	-1.65305	-7.06513	6.19483	H	1.35033	5.03379	1.21645
H	-8.76318	-6.73297	-4.80154	H	11.00707	7.53975	3.76787
H	8.77213	-7.21182	3.52788	H	-11.04806	8.06065	-2.55855

H	-8.22931	1.55892	-10.69236	H	-7.70993	4.39758	-0.03136
H	-2.59584	-3.65928	-9.08139	H	-1.64959	8.89817	3.02244
H	-3.80726	2.03106	-3.18713	H	-3.74517	5.41080	1.60613
H	-4.40628	2.14433	-1.52119	H	-3.22685	11.87235	0.65895
H	-3.15738	0.98207	-1.90246	H	-7.28827	12.58887	-0.58953
H	-7.28848	-2.81987	-0.67616	H	-7.29205	-6.80093	-10.60663
H	-8.61701	-2.38148	-1.75835	H	-7.71819	6.21168	5.76128
H	-8.19042	-1.34056	-0.40137	H	-7.86491	10.48763	5.22511
H	-2.59983	-6.03489	7.70893	H	-4.96636	-6.90167	-11.55560
H	-6.99205	4.80736	3.88730	H	-4.33244	-1.69092	4.33996
H	-2.59472	9.69539	1.37216	H	-3.95789	-1.85454	8.62460
H	-7.76352	10.19411	-0.11575	H	-4.79321	-4.46767	1.69483
H	-7.76604	-5.19267	-8.76974	H	-7.71225	-2.17000	3.82025
H	-7.00672	10.13897	2.91174	H	-7.72178	-8.09306	2.49313
H	-3.22929	-5.36520	-10.60994	H	-7.87078	-9.76802	6.46365
H	-6.40267	-0.89872	7.57942	H	-8.76417	-0.78961	8.23251
H	-6.99489	-5.76810	2.21506	H	-11.36972	-4.06074	7.17896
H	-7.01236	-7.59076	7.31951	H	-7.29607	-5.78401	11.19203
H	-9.38954	-5.20159	6.17455	H	-4.97056	-6.55602	11.75380
H	-7.76979	-4.99706	8.88086	H	-4.96262	13.46023	-0.20021
H	-3.23344	-6.50556	9.95040	H	-8.22994	8.48287	6.69332
H	-1.75749	2.02940	6.67720	H	-7.28727	1.99753	-2.10674
H	1.41887	0.40339	4.27804	H	-8.61603	2.71585	-1.18658
H	-1.69632	-0.85535	8.46467	H	-8.18988	1.02015	-0.96332
H	-2.07432	-0.67972	4.18492	H	-4.40779	0.24808	2.61866
H	0.14018	7.47840	3.85927	H	-3.80570	1.74806	3.34965
H	-1.97012	3.97951	2.50347	H	-3.15870	1.15589	1.79916
H	-1.47466	4.21021	5.57806	H	-3.15772	-2.13681	0.10226
H	1.71335	2.58792	3.19074	H	-3.80847	-3.77330	-0.16522
H	-4.79131	3.70513	3.02142	H	-4.40596	-2.38626	-1.09592

H	-8.19012	0.32683	1.36191	H	4.49364	6.47996	4.76681
H	-7.28802	0.82772	2.78059	H	2.67638	10.05038	-0.39989
H	-8.61721	-0.32792	2.94173	F	-4.00554	0.00137	-0.00059
H	-8.23484	-10.03617	3.99312	H	7.36231	-5.09876	-3.18643
H	-11.05268	-1.81327	8.25437				

6 References

[1] Y. Yang, T. K. Ronson, J. Zheng, N. Mihara, J. R. Nitschke, *Chem* **2023**, *9*, 1972–1982.

[2] P. J. Stephens, F. J. Devlin, C. F. Chabalowski, M. J. Frisch, *J. Phys. Chem.* **1994**, *98*, 11623–11627.

[3] P. J. Hay, W. R. Wadt, *J. Chem. Phys.* **1985**, *82*, 270–283.

[4] R. Ditchfield, W. J. Hehre, J. A. Pople, *J. Chem. Phys.* **1971**, *54*, 724–728.

[5] G. A. Petersson, M. A. Al-Laham, *J. Chem. Phys.* **1991**, *94*, 6081–6090.

[6] M. J. Frisch, G. W. Trucks, H. B. Schlegel, G. E. Scuseria, M. A. Robb, J. R. Cheeseman, G. Scalmani, V. Barone, G. A. Petersson, H. Nakatsuji, X. Li, M. Caricato, A. V. Marenich, J. Bloino, B. G. Janesko, R. Gomperts, B. Mennucci, H. P. Hratchian, J. V. Ortiz, A. F. Izmaylov, J. L. Sonnenberg, D. Williams-Young, F. Ding, F. Lipparini, F. Egidi, J. Goings, B. Peng, A. Petrone, T. Henderson, D. Ranasinghe, V. G. Zakrzewski, J. Gao, N. Rega, G. Zheng, W. Liang, M. Hada, M. Ehara, K. Toyota, R. Fukuda, J. Hasegawa, M. Ishida, T. Nakajima, Y. Honda, O. Kitao, H. Nakai, T. Vreven, K. Throssell, J. A. , Jr. Montgomery, J. E. Peralta, F. Ogliaro, M. J. Bearpark, J. J. Heyd, E. N. Brothers, K. N. Kudin, V. N. Staroverov, T. A. Keith, R. Kobayashi, J. Normand, K. Raghavachari, A. P. Rendell, J. C. Burant, S. S. Iyengar, J. Tomasi, M. Cossi, J. M. Millam, M. Klene, C. Adamo, R. Cammi, J. W. Ochterski, R. L. Martin, K. Morokuma, O. Farkas, J. B. Foresman, D. J. Fox, **2016**.

[7] J. B. Maglic, R. Lavendomme, *J. Appl. Crystallogr.* **2022**, *55*, 1033–1044.

[8] J. A. Davies, T. K. Ronson, J. R. Nitschke, *J. Am. Chem. Soc.* **2024**, *146*, 5215–5223.

[9] D. R. Allan, H. Nowell, S. A. Barnett, M. R. Warren, A. Wilcox, J. Christensen, L. K. Saunders, A. Peach, M. T. Hooper, L. Zaja, S. Patel, L. Cahill, R. Marshall, S. Trimnell, A. J. Foster, T. Bates, S. Lay, M. A. Williams, P. V Hathaway, G. Winter, M. Gerstel, R. W. Wooley, *Crystals* **2017**, *7*, 336.

[10] P. Evans, in *Acta Crystallogr D Biol Crystallogr*, International Union Of Crystallography, **2006**, *62*, 72–82.

[11] G. Winter, *J. Appl. Crystallogr.* **2010**, *43*, 186–190.

[12] G. Winter, D. G. Waterman, J. M. Parkhurst, A. S. Brewster, R. J. Gildea, M. Gerstel, L. Fuentes-Montero, M. Vollmar, T. Michels-Clark, I. D. Young, N. K. Sauter, G. Evans, *Acta Crystallogr. D Struct. Biol.* **2018**, *74*, 85–97.

[13] L. J. Farrugia, *J. Appl. Crystallogr.* **2012**, *45*, 849–854.

[14] P. R. Evans, G. N. Murshudov, *Acta Crystallogr. D Biol. Crystallogr.* **2013**, *69*, 1204–1214.

[15] M. D. Winn, C. C. Ballard, K. D. Cowtan, E. J. Dodson, P. Emsley, P. R. Evans, R. M. Keegan, E. B. Krissinel, A. G. W. Leslie, A. McCoy, S. J. McNicholas, G. N. Murshudov, N. S. Pannu, E. A. Potterton, H. R. Powell, R. J. Read, A. Vagin, K. S. Wilson, *Acta Crystallogr. D Biol. Crystallogr.* **2011**, *67*, 235–242.

[16] G. M. Sheldrick, *Acta Crystallogr. A* **2015**, *71*, 3–8.

[17] G. M. Sheldrick, *Acta Crystallogr. C Struct. Chem.* **2015**, *71*, 3–8.

[18] P. Van Der Sluis, A. L. Spek, *Acta Crystallogr. A* **1990**, *46*, 194–201.

[19] A. L. Spek, PLATON: A Multipurpose Crystallographic Tool. Utrecht University: Utrecht, The Netherlands.; **2008**.
